# Delphi-driven consensus definition for mesenchymal stromal cells and clinical reporting guidelines for mesenchymal stromal cell-based therapeutics

**DOI:** 10.1016/j.jcyt.2024.10.008

**Published:** 2024-10-29

**Authors:** Laurent Renesme, Kelly D. Cobey, Manoj M. Lalu, Tania Bubela, Raghavan Chinnadurai, John De Vos, Rod Dunbar, Dean Fergusson, Daniel Freund, Jacques Galipeau, Edwin Horwitz, Maxime Lê, Michael Matthay, David Moher, Jan Nolta, Graham Parker, Donald G. Phinney, Mahendra Rao, John E.J. Rasko, Patricia R.M. Rocco, Fabio Rossi, Michael Rosu Myles, Sowmya Viswanathan, Bernard Thébaud

**Affiliations:** 1 Sinclair Center for Regenerative Medicine, Ottawa Hospital Research Institute, Ottawa, Canada; 2 Neonatology, Department of Pediatrics, Children’s Hospital of Eastern Ontario, Ottawa, Canada; 3 University of Ottawa Heart Institute, Ottawa, Canada; 4 Department of Anesthesiology and Pain Medicine, The Ottawa Hospital, Ottawa, Canada; 5 Clinical Epidemiology Program and Regenerative Medicine Program, Ottawa Hospital Research Institute, Ottawa, Canada; 6 Department of Cellular and Molecular Medicine, University of Ottawa, Ottawa, Canada; 7 Faculty of Health Sciences, Simon Fraser University, Burnaby, Canada; 8 Department of Biomedical Sciences, Mercer University School of Medicine, Savannah, Georgia, USA; 9 Institute for Regenerative Medicine and Biotherapy, Montpellier University, Montpellier, France; 10 School of Biological Sciences and Maurice Wilkins Centre, University of Auckland, Auckland, New Zealand; 11 Department of Medicine, University of Ottawa, Ottawa, Canada; 12 Ottawa Hospital Research Institute, Ottawa, Canada; 13 Department of Neonatology and Pediatric Critical Care Medicine, Dresden University of Technology, Dresden, Germany; 14 Center for Regenerative Therapies Dresden, Dresden University of Technology, Dresden, Germany; 15 University of Wisconsin School of Medicine and Public Health, University of Wisconsin, Madison, Wisconsin, USA; 16 Winship Cancer Institute, Emory University School of Medicine, Atlanta, Georgia, USA; 17 Patient Partner, Ottawa Hospital Research Institute, Ottawa, Canada; 18 Cardiovascular Research Institute, Departments of Medicine and Anesthesia, University of California, San Francisco, California, USA; 19 Ottawa Methods Centre, Ottawa Hospital Research Institute, Ottawa, Canada; 20 Institute for Regenerative Cures, University of California Davis Health, Sacramento, California, USA; 21 Department of Pediatrics, Wayne State University School of Medicine, Detroit, Michigan, USA; 22 Department of Molecular Medicine, The Herbert Wertheim UF Scripps Institute for Biomedical Innovation and Technology, Jupiter, Florida, USA; 23 Vita Therapeutics, Baltimore, Maryland, USA; 24 Department of Cell and Molecular Therapies, Royal Prince Alfred Hospital, Sydney, Australia; 25 Faculty of Medicine and Health, University of Sydney, Sydney, Australia; 26 Laboratory of Pulmonary Investigation, Carlos Chagas Filho Institute of Biophysics, Federal University of Rio de Janeiro, Rio de Janeiro, Brazil; 27 National Institute of Science and Technology for Regenerative Medicine, Rio de Janeiro, Brazil; 28 Biomedical Research Centre, University of British Columbia, Vancouver, Canada; 29 Biologic and Radiopharmaceutical Drugs Directorate, Health Products and Food Branch, Health Canada, Ottawa, Canada; 30 Schroeder Arthritis Institute, University Health Network, Toronto, Canada; 31 Krembil Research Institute, University Health Network, Toronto, Canada; 32 Institute of Biomedical Engineering, University of Toronto, Toronto, Canada; 33 Department of Medicine, University of Toronto, Toronto, Canada; 34 Children’s Hospital of Eastern Ontario Research Institute, Ottawa, Canada

**Keywords:** consensus definition, Delphi method, mesenchymal stromal cells, reporting guidelines

## Abstract

**Background aims:**

Despite promising results in pre-clinical studies, mesenchymal stromal cells (MSCs) face significant challenges in clinical translation. A scoping review by our group highlighted two key issues contributing to this gap: (i) lack of a clear and consensus definition for MSCs and (ii) under-reporting of critical parameters in MSC clinical studies. To address these issues, we conducted a modified Delphi study to establish and implement a consensus definition for MSCs and develop reporting guidelines for MSC clinical studies.

**Methods:**

A steering committee of 22 international experts, including stakeholders from different MSC research fields, participated in the three Delphi rounds. For the first round, to obtain a broad perspective, additional investigators recommended by the steering committee were invited to participate. The first two rounds consisted of online surveys, whereas the third round took the form of a virtual meeting. Participants were asked to rate a series of potential defining characteristics of MSCs and items for reporting guidelines. Consensus was defined as at least 80% of the participants rating the item in the same category of importance.

**Results:**

Eighty-seven international participants participated in the first round survey (spring 2023), 17 participants participated in the second online survey (fall 2023) and 15 participants participated in the final virtual consensus meeting (January 2024). For the MSC definition, 20 items were considered and nine reached consensus. Items included terminology (one item), cell marker expression (five items), tissue origin (one item), stemness (one item) and description of critical quality attributes (one item). For the reporting guidelines, with the 28 initial items and the additional items suggested during round 1, a total of 33 items to report were included. This included items on MSC intervention group and control (e.g., MSC product, dose and administration), MSC characteristics (e.g., MSC provenance, “fitness,” viability and immune compatibility) and MSC culture conditions (e.g., oxygen environment, culture medium and use of serum).

**Conclusions:**

By applying a Delphi method to establish a consensus definition for MSCs and reporting guide-lines for MSC-based clinical trials, this work represents a significant advance in improving transparency and reproducibility in the conduct and reporting of MSC research.

## Introduction

Since mesenchymal stromal cells (MSCs) were first tested as a therapeutic agent in 1995 [1], over 1000 MSC clinical trials have been registered on ClinicalTrials.gov [2]. Despite promising results for MSCs in different pre-clinical disease models, clinical trials using MSCs in various medical conditions have provided less encouraging results. Several factors have been suggested to explain the challenges in the clinical translation of MSC-based products, one of the most critical being that pre-clinical and clinical studies using MSCs exhibit many disparities with respect to MSC characteristics (e.g., characterization, immune compatibility, cell viability and dose) [3].

The International Society for Cell & Gene Therapy first provided minimal criteria to define MSCs in 2006 [4]. These criteria were determined through an informal consensus of a small number of leading experts. Multiple critiques of this definition have been raised since its publication, including differentiation assays that are prone to misinterpretation, limitations in functionally defining stromal cells and the fact that the definition does not account for developmental origins of tissue sources. In addition, the frequent use of the denomination mesenchymal “stem cells” creates confusion about these cells’ function, and some authors advocated to “clear up this stem cell mess” [5]. Consequently, the definition was updated in 2019. However, the International Society for Cell & Gene Therapy MSC committee adopted an informal method, and consensus could not be reached on many items discussed [6]. Thus, the MSC research community still lacks an updated definition that addresses the criticisms raised.

In a recent scoping review describing how MSCs were defined and characterized in pre-clinical and clinical studies, some of us (LR, KDC, MML and BT) highlighted the impact caused by the absence of an updated consensus definition. Among 318 original studies, the analysis revealed major inconsistences in defining MSCs and in reporting MSC characteristics and culture conditions. In addition, for clinical studies using MSCs, relevant information on MSC characterization and cell manufacturing process was incompletely reported [7]. This scoping review highlighted broad variations in criteria used to define MSCs, tissue sources, cell characteristics and culture conditions, hindering the ability to reproduce or compare these studies.

To improve clinical translation, reproducibility and transparency in the field of MSC research, the scientific community needs to reach a consensus on how to define MSCs. The aim of this project was to use a formal consensus process—namely, the Delphi method—to develop a consensus-based definition of MSCs and reporting guidelines for clinical trials using MSC therapy. This approach addresses many pitfalls of previous attempts to develop a consensus definition of MSCs that may not have optimally engaged the research community in the process or structured engagement and dialogue effectively. The Delphi method is a structured communication method that relies on a panel of experts and several rounds of questionnaires to reach a consensus [8]. The Delphi method addresses limitations of less formal approaches to group decision-making (e.g., difficulties bringing people together; adequately accounting for divergent opinions, peer pressure and influence in the decision-making process; group influences on individual performance) that may explain why previous attempts to define MSCs, which relied on group decision-making, failed to reach a consensus. This approach is routinely used to build consensus around contentious issues [9] and reporting guidelines [10]. This project was implemented using an “integrated knowledge approach” whereby we engaged a large international group of stakeholders. Contributors provided expertise in the different fields of MSC research to support the development, dissemination and implementation of both the consensus definition and the reporting guidelines (“integrated knowledge translation approach”).

## Methods

### Ethics statement

This study received ethical approval from the Ottawa Health Science Network Research Ethics Board (research ethics board protocol identifier 20210187–01K). Participants received a recruitment e-mail before viewing round 1 of the Delphi; their completion of the survey was considered implied consent.

### Transparency statement

Protocols for the overall program of research (scoping review and Delphi study) were posted on the Open Science Framework [11]. All related study materials and data are available at https://osf.io/3dsqx/. In addition, the study protocol was published in an open access peer-reviewed journal [12].

To establish a consensus definition for MSCs and the reporting guidelines for clinical studies using MSCs, we conducted an international three-round modified Delphi survey. This report followed the Accurate Consensus Reporting Document guidelines (see supplementary Appendix 1) [13]. The initial Delphi questionnaire, the modified questionnaire for the second round and the material used for the virtual meeting are available as supplementary material (see supplementary Appendixes 2–5).

### Participants

For the first online round, invitations were sent to the steering committee (co-authors of this report; LR, KDC, MML, TB, RC, JDV, RD, DFe, DFr, JG, EH, ML, MM, DM, JN, GP, DGP, MR, JEJR, PRMR, FR, MRM, SV, BT) and the corresponding authors of pre-clinical and clinical studies using MSCs identified through our scoping review [7]. In addition, because of the low participation from the participants identified through the scoping review, we asked each steering committee member to suggest five potential participants with expertise in MSCs. For the second online round and the third round (virtual consensus meeting), only the steering committee received an invitation to participate. This approach was taken to maximize the initial reach of our survey while maintaining a manageable number of individuals to facilitate our round three virtual consensus meeting.

We invited a group of experts to join the steering committee of our Delphi survey. The steering committee was recruited using an “integrated knowledge translation approach.” This approach relies on identifying and integrating representative knowledge users (e.g., scientists, trialists, manufacturers, journal editors) into the project from its inception so that those with the authority to implement the future recommendations have their needs and preferences considered during the consensus-building process. This approach helps to ensure that the methods used to generate the recommendations resonate with the stakeholders and support high levels of commitment from inception to dissemination [14]. The steering committee comprised international key stakeholders from different fields of MSC research, including developmental biology, pre-clinical and clinical research, research methods, regulatory practices, scholarly journal editing and industry. The steering committee roles were to (i) review the initial Delphi survey, (ii) recommend additional participants for the first online round, (iii) participate in the three rounds and (iv) support dissemination and implementation of the results (“integrated knowledge translation approach”). With their invitation, steering committee members received the scoping review and study protocol and letter to the editors’ publications [7,12,15].

### Delphi approach

To establish a consensus definition for MSCs, we conducted a three-round modified Delphi survey. To ensure participants’ anonymity, the first two rounds (spring and fall 2023) were completed online using Surveylet (Calibrum, Saint George, UT, USA), a dedicated platform for Delphi surveys [16]. Invitations were sent by e-mail with a reminder at 7 days and 14 days. The last round consisted of two half-day virtual consensus meetings (January 2024) hosted on Zoom (Zoom Video Communications, San Jose, CA, USA) [17].

The initial questionnaire was developed using items identified from a scoping review aiming to describe how MSCs are defined and characterized in published pre-clinical and clinical studies [7]. The initial survey had three sections: (i) participant demographics, (ii) items to define MSCs and (iii) items for reporting guidelines. The MSC definition section included 20 potential characteristics to define MSCs, with items on terminology (two items), plastic adherence (one item), cell marker expression (five items), differentiation assays (four items), tissue origin (two items), evidence of stemness *in vitro* (two items), *in vitro* functional assays (one item) and MSC licensing (three items). The reporting guidelines section included 28 items divided into three parts: description of MSC intervention and control groups (nine items), MSC characteristics (11 items) and MSC culture conditions (eight items). MSC intervention and control groups included items on MSC product, dose, administration route and control group used. MSC characteristics included items on MSC provenance, immune compatibility, “fitness” and viability. MSC culture conditions included items on oxygen environment, cell confluence, culture medium and use of serum or human platelet lysate.

The initial questionnaire was reviewed by the steering committee to ensure item clarity and that it captured all important themes relevant to consider for defining MSCs (see supplementary Appendix 2). For the two online rounds, participants were asked to rate each survey item’s importance on a scale of 1 (unessential) to 9 (essential). For each item, a text box was provided to allow participants to provide rationale for their rating. In addition, during the first and second rounds, at the end of the survey, participants had the opportunity to suggest any new item they felt was missing and essential to define MSCs or to be added to the reporting guidelines. Consensus was reached when at least 80% of the participants rated the item in the upper or lower third of the scale (1–3, unessential, or 7–9, essential). In subsequent rounds, participants were informed of included and excluded items but were not asked to vote on those items. Items that did not achieve consensus and new items suggested by participants were used to generate a new survey for the subsequent Delphi round. The modified questionnaire used for round 2 is presented in supplementary Appendix 3.

The third round was a virtual consensus meeting. Prior to the virtual meeting, the steering committee members received a consensus meeting handbook (see supplementary Appendix 4), which provided context in terms of how the two half-day virtual meetings would be facilitated, and a list of items that had reached consensus within rounds 1 and 2. They were also given a list of items that had not reached consensus, including feedback from round 2 participants, which needed to be revoted on (see supplementary Appendix 5). During the meeting, each item was discussed between participants, and a forced-choice vote was conducted using the categories “include,” “exclude” and “abstain.” Voting was anonymous and conducted using the polling function on Zoom [17].

### Statistical analysis

After each round, data were anonymously analyzed using Excel (Microsoft, Redmond, WA, USA). For each item, the percentage of voting participants in each scale category (1–3, 4–6 and 7–9) was calculated. When 80% or more of the voting participants selected the upper third of the scale (7–9, essential), the item was included in the MSC definition or the reporting guidelines. When 80% or more of the voting participants selected the lower third of the scale (1–3, unessential), the item was excluded. During subsequent rounds, participants were informed of included and excluded items during previous rounds. Items that did not reach consensus were integrated into the new questionnaire to be voted on during the next round. Voting results and participants’ comments from the previous round were presented to the participants. Participants’ comments were reviewed and categorized according to participants’ votes into one of three categories: pro, neutral or con.

## Results

**Fig. 1.**
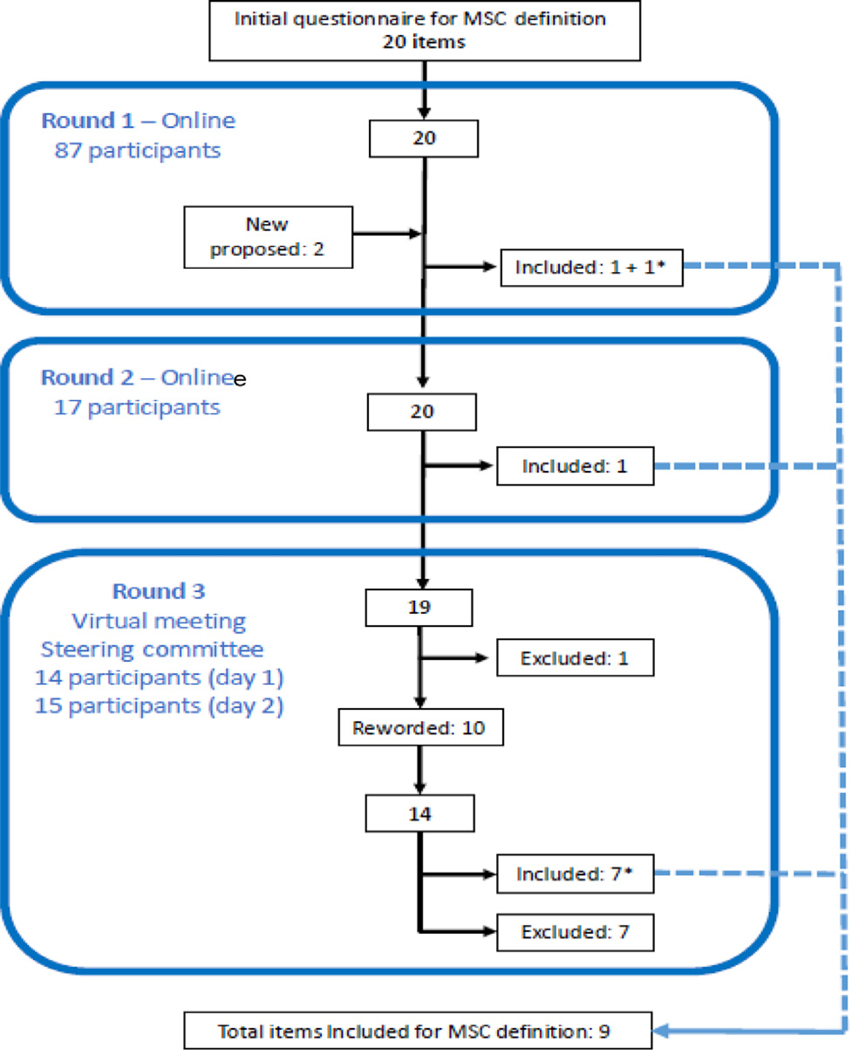
Flowchart of items for MSC definition and characterization. *One of the items included in round 1 was reworded and revoted on. (Color version of figure is available online.)

**Fig. 2.**
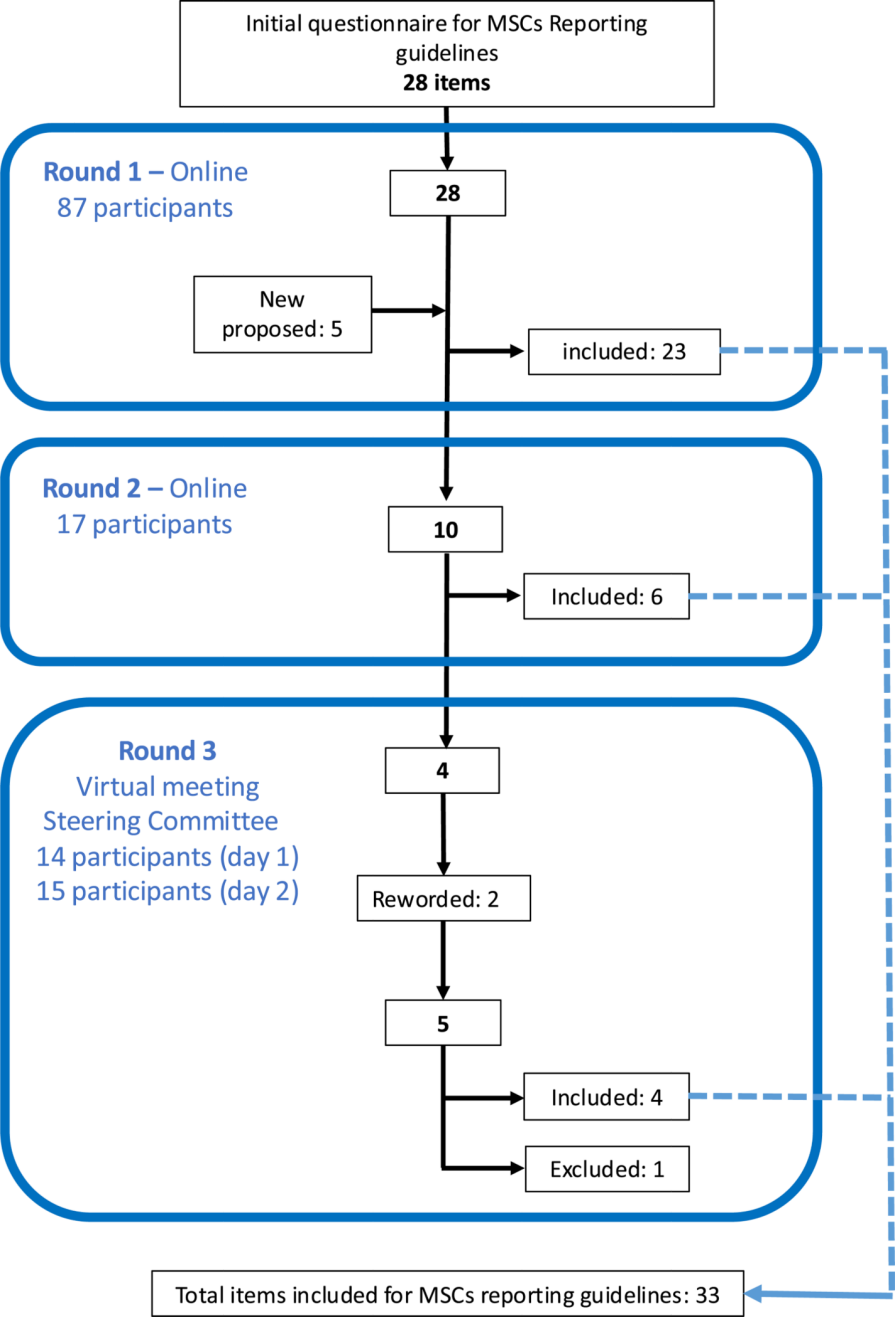
Flowchart of items for reporting guidelines for clinical studies using MSCs. (Color version of figure is available online.)

The item flowcharts are presented in Figure 1 (items for MSC definition) and Figure 2 (items for reporting guidelines). The Delphi participant flowchart is presented in supplementary Figure 1.

### Round 1 results

#### Participants

A total of 87 participants (male, n = 55 [63%]) from 22 countries completed round 1. Eighty-two participants (94%) were actively conducting MSC research at the time of the survey. Most of the participants reported conducting clinical (n = 39 [45%]), pre-clinical (n = 59 [68%]) and basic (n = 56 [64%]) research. The top three research areas reported were cell therapy (n = 51 [59%]), blood and immune system (n = 31 [36%]) and musculoskeletal system (n = 24 [28%]). Full demographics of participants are described in Table 1.

### Voting

#### MSC definition

Of the initial 20 items to define MSCs, two reached consensus in round 1. Participants agreed that “a description of MSCs’ positive and negative cell surface markers is essential to define them.” Among the positive and negative cell surface markers proposed in the survey, participants agreed that CD73+, CD90+, CD105+ and CD45- were essential to define MSCs. The second item agreed to was “a description of where the MSCs were sourced from is essential to characterize them,” and participants included the following tissues as a source of MSCs: bone marrow, umbilical cord (Wharton jelly), adipose tissue and placenta/amnion.

Participants suggested two additional items to define MSCs in round 1. The first new item pertained to alternative terms for MSCs and included the following: vascular maintenance cell, multipotent stromal cell, fibroblastic stromal cell, tissue-derived stromal cell, cultured stromal cell (followed by tissue of origin), mesenchymal signaling cell and mesenchymal stromal-derived cell. The second new item for “additional characteristics that are essential to define or characterize MSCs” included the following propositions: transcriptome analysis (e.g., single-cell RNA sequencing or bulk RNA sequencing), secretome profile, exosomes (exosome signature, quantitative measurement), immunomodulatory and mixed lymphocyte reaction assays, angiogenic assay, transcription factor expression and DNA methylation profile. Along with these new items, participants suggested additional positive cell markers (CD10+, CD140+, CD142+, CD271+, CD276+, HLA-I+, SSEA3+ (stage-specific embryonic antigen 3), SSEA4+ (stage-specific embryonic antigen 4), nestin+) as well as additional novel tissue sources (dental follicle, menstrual blood, “induced pluripotent stem cell and fetal tissue”, “most tissues [excluding or including central nervous system]”, amniotic fluid and “virtually all tissues”). First round results for the MSC definition are presented in Table 2.

#### Reporting guidelines

Of the initial 28 items for the reporting guidelines, 23 reached consensus. For the section describing MSC intervention and control groups, participants reached consensus to include the following reporting items: MSC administration route (e.g., intravenous, intra-articular), MSC dose (i.e., number of cells per kilogram of body weight), MSC product concentration (i.e., number of cells per milliliter of vehicle), vehicle used to deliver MSCs to the patient, MSC infusion rate (if intravenous administration route) and adjuvant used during MSC preparation. For studies using a control group, control group characteristics and type of control used should be reported. For the MSC characteristics section, nine items were included. Three items were related to MSC provenance (MSC provenance [e.g., patient, donor, company], MSC tissue source and extraction procedure to obtain MSCs from tissue); one item was related to MSC immune compatibility (e.g., autologous, unmatched allogeneic, matched allogeneic); two items were related to MSC “fitness” (MSC status prior to administration [fresh versus cryopreserved] and, for studies using cryopreserved MSCs, MSC culture recovery status prior to administration); and three items were related to MSC viability (viability assessment performed prior to MSC administration, description of viability assay used and viability assay results). Finally, for the MSC culture conditions section, six items reached consensus for inclusion in the reporting guidelines. These items included the level of oxygen used for MSC culture, the use (or not) of serum for the culture (and if serum was used, the type and concentration should be reported) and the use of human platelet lysate for the culture (and the amount used).

Participants suggested five new items for the reporting guidelines during round 1. One item was about MSCs’ provenance in the MSC characteristics section (“MSC donor characteristics [e.g., age, sex, body mass index] should be extensively described”), two items were about MSC “fitness” (“any functional assay performed on the cell product should be reported” and “describing whether the MSCs used in the clinical trial are derived from the same batch or different batches is important to report”) and the last two suggested items were about MSC culture conditions (“the method used to culture the MSCs [two-dimensional {2D} versus three-dimensional {3D} culture] should be reported” and “the medium and reagent catalog numbers should be reported in the methods section”). First round results for the reporting guidelines are presented in Table 3.

### Round 2 results

#### Participants

Of the 22 members of the steering committee invited, 17 (77%) from six countries participated in round 2. Sixteen participants (94%) were conducting research at the time of the survey. Most of the participants reported more than 15 years of research (n = 12 [75%]) in clinical (n = 10 [59%]), pre-clinical (n = 11 [65%]) and basic (n = 9 [53%]) research. The top three research areas reported were cell therapy (n = 9 [53%]), blood and immune system (n = 6 [35%]) and respiratory system (n = 5 [29%]). Full demographics of participants for round 2 are described in Table 1.

### Voting

#### MSC definition

Of the 20 items presented, only one reached consensus during round 2. For MSC terminology, 13 participants (87%) agreed that “‘mesenchymal stromal cell’ is an appropriate term to maintain.” Round 2 results for the MSC definition are presented in Table 2. For the item “tissue origin,” participants agreed that dental follicle is also another source of MSCs.

#### Reporting guidelines

Of the 10 items presented, six reached consensus during round 2. Participants agreed that the following items should be part of the reporting guidelines for MSC characteristics: MSC donor characteristics, any functional assay performed on the MSC product and whether MSCs are derived from the same batch or different batches. For MSC culture conditions, the method used to culture the MSCs (2D versus 3D), the level of cell confluence used to harvest the cells prior to administration to the patient and the culture medium used to culture MSCs were deemed essential items to report. Round 2 results are presented in Table 3.

### Round 3 results

#### Participants

Of the 22 members of the steering committee, 14 (64%) participated in day 1 and 15 (68%) participated in day 2 of the consensus meeting. Full demographics of participants are described in Table 1.

### Voting

#### MSC definition

Of the 19 items that did not reach consensus during round 2, after discussion among participants, some were reworded, merged into one new item or expanded into two items. For example, for evidence of stemness, the initial questionnaire included the following two items: “a description of self-renewal and multilineage differentiation capacities is essential to define MSCs” and “the description of specific method used to assess MSCs’ stemness *in vitro* is essential to define MSCs.” After discussion with the group, these two items were summarized as one new item: “if authors use the term ‘stem’ (i.e., mesenchymal stem cell), a description of the methods used to demonstrate stemness must be provided.”

As the item “‘mesenchymal stromal cell’ is an appropriate term to maintain” reached consensus during round 2, the item “alternative terms” for MSCs was removed prior to voting. Participants ended up voting on 14 items (five items on day 1, nine items on day 2) (see supplementary Appendix 5). Third round results for the MSC definition items are presented in Table 2 and the items included and excluded from the MSC definition during the different rounds are presented in Table 4.

#### Reporting guidelines

Of the four items that did not reach consensus during round 2, after discussion among participants, some were reworded or expanded into two items. As a result, participants voted on five items (see supplementary Appendix 5). Of the five items, four reached consensus for inclusion as part of the reporting guidelines. One item was about MSC intervention and control groups (“the MSC dose should be reported as a dose normalized to weight [number of cells per kilogram of body weight]; if this is not provided, please explain the rationale”), two items were about MSC characteristics (“for studies using cryopreserved MSCs, the number of months the cells were frozen prior to patient administration should be described; the range, mean and median number of months should be reported” and “for studies using cryopreserved MSCs, the temperature at which the cells were frozen prior to patient administration should be described; the range, mean and median temperature should be reported”) and one item was about MSC culture conditions (“the medium and reagent catalog numbers should be reported in the methods section”).

Finally, one item did not reach consensus for inclusion and was therefore removed from the reporting guidelines (“the population doubling time of the MSCs used in the intervention group should be reported”). Results from the third round for the reporting guidelines items are presented in Table 3. The items included and excluded from the reporting guidelines during the different rounds are presented in Table 5.

#### Consensus on minimal criteria to define and characterize MSCs

A total of nine items were ultimately included in the MSC consensus definition and are presented in Table 6.

#### Terminology

“‘Mesenchymal stromal cell’ is an appropriate term to maintain.”

#### Rationale.

Participants agreed to maintain the denomination of MSCs for the following reasons:

- MSCs include a heterogeneous population of cells isolated from a range of different tissues. “Mesenchymal stromal cell” should be used as an umbrella term with further clear description of species, tissue origin and any other relevant attribute to characterize them.
- MSCs’ main therapeutic effects *in vivo* are linked to their secretory actions rather than stemness properties.
- The body of research built on the acronym “MSC” is considerable. To any lay audience or newcomer, that continuity as well as name recognition of “mesenchymal stromal cell” is key.

#### Cell surface markers

- A description of MSCs’ positive and negative markers is essential to define them.
- For MSC marker expression, the flow cytometry cutoff (% of cells) to consider a cell marker a positive or negative marker should be detailed in the methods section.
- For MSC marker expression, the flow cytometry results with the % of positive cells should be described for each positive and negative marker.
- Positive cell markers used to define MSCs should be reported. Reliable examples include CD73+, CD90+ and CD105+; additional markers used should also be reported.
- Negative cell markers used to define MSCs should be reported (CD45- should always be reported); additional tissue-relevant markers used should also be reported.

#### Rationale.

Participants agreed that even though there is currently no specific marker for MSCs, a combination of positive and negative phenotypic cell surface markers can be used to define cell populations. Reporting positive and negative cell markers for a studied MSC population is critical to characterize the population and ensure its purity, which are two key determinants of reproducibility and transparency in MSC research. It was recognized that although it is important to have defined cell markers, there is a need for flexibility because MSC markers vary depending on tissue origin and also change during the *in vitro* culture expansion. During the third round, participants strongly recommended the use of CD45- as a negative cell surface marker to validate the absence of contamination with hematopoietic lineage populations. Reporting flow cytometry cutoff was described as a standard requirement for all markers.

#### Tissue origin

“A description of where the MSCs were sourced from is essential to characterize them. Any tissue can be noted as the source.”

#### Rationale.

All participants agreed on the importance of reporting the tissue source, as it influences MSCs’ phenotype and functions. Regarding the tissues that could be a potential source for MSCs, it was acknowledged that with the development of new omics approaches at a single-cell resolution, researchers might be able to characterize MSCs from any kind of tissue, including the brain. As the MSC definition is a moving definition that will be challenged by the development of new methods for cell characterization, it was decided to avoid being prescriptive of a specific tissue type; this will also support the definition’s sustainability. Regarding the clinical use of MSCs as therapeutic, it was stressed that thus far only a few tissue sources have been tested in clinical trials, and these should obviously be cited when reported.

#### Evidence of stemness

“If the author uses the term ‘stem’ (i.e., mesenchymal stem cell), a description of the methods used to demonstrate stemness must be provided.”

#### Rationale.

Participants discussed that the use of the term “mesenchymal stem cell” in pre-clinical and clinical studies is more often related to misuse or confusion around the terminology of MSCs rather than a claim of stemness properties. As noted earlier, consensus was reached to keep “mesenchymal stromal cell” and exclude “mesenchymal stromal cells and mesenchymal stem cells are interchangeable terms.” Therefore, participants agreed that if authors use the term “stem” and claim stemness properties of the MSCs they are using, they must provide evidence of stemness. However, no consensus was reached on the specific experimental approach or data that constitute “evidence of stemness.”

#### MSCs’ potency and properties

“A description of critical quality attributes to assess MSCs’ potency and properties is essential to characterize MSCs for clinical use.”

#### Rationale.

Participants discussed that even though defining the characteristics and mechanism of action of MSCs is emerging, a comprehensive analysis of cell biology quality attributes to assess function and biologically plausible potency is needed to ensure transparency and reproducibility in MSC clinical research.

#### Consensus on reporting guidelines for MSC clinical studies

A total of 33 items were included in the reporting guidelines and are presented in Table 7. Two common themes emerged around the rationale to include the following items in the reporting guidelines. Participants insisted on the importance of detailed and comprehensive information about the MSC product used in clinical trials to (i) ensure participants’ safety and (ii) allow comparison between studies and generate additional knowledge using systematic reviews with meta-analyses.

#### MSC intervention and control groups

In MSC clinical trials, the following details should be reported for the MSC intervention and control groups:

- MSC administration route.
- MSC dose.
- The MSC dose as a dose normalized to weight (number of cells per kilogram of body weight). If this is not being provided, authors should explain the rationale.
- MSC product concentration (i.e., concentration [number of cells per milliliter of vehicle] of the cell product administered to the patient).
- The vehicle in which MSCs are delivered to the patient.
- For MSC clinical studies using intravenous route for MSC administration, the MSC suspension infusion rate.
- The use of adjuvants during the preparation or processing of MSCs (e.g., use of dimethyl sulfoxide for MSC preparation).
- When the study design involves a control group, the characteristics of the control group.
- The type of control used (e.g., vehicle, normal saline).

#### Rationale

A comprehensive description of the intervention and control groups is key to ensuring transparency, reproducibility and interpretation. This information is also critical for comparison between studies and standardization. Details on administration route, dose and product concentration and composition (including vehicle and adjuvants) are basic requirements for any pharmacological study (including cell-based therapy). These parameters will influence the pharmacodynamics and pharmacokinetics of a therapy and are crucial to define the therapeutic safety and efficacy profiles. Vehicle and adjuvants can influence the viability and function of a cell product. Reporting vehicle and adjuvants used in a study will help to understand the interaction between these and the cells and to optimize the safety and efficacy of the MSC product. In addition, both vehicle and adjuvants may have potential side effects for the patient; therefore, the same vehicle and adjuvants should be used in the control group.

#### MSC characteristics

In MSC clinical trials, the following details should be reported for the MSC characteristics:

- MSC provenance (e.g., MSC provenance can be from patient, donor or cell from stem cell company).
- MSC donor characteristics (e.g., age, sex, body mass index, health status, medication, smoking).
- The tissue source of the MSCs (e.g., bone marrow, adipose tissue).
- The extraction procedure used to obtain MSCs from the tissue source (e.g., enzymatic digestion, mechanical).
- The immune compatibility between the MSC product and participant (e.g., autologous, unmatched allogeneic, matched allogeneic).
- MSC state prior to administration (e.g., fresh versus cryopreserved).
- For studies using cryopreserved MSCs, the number of months the cells were frozen prior to patient administration should be described. The range, mean and median number of months should be reported.
- For studies using cryopreserved MSCs, the temperature at which the cells were stored prior to participant administration should be described. The range, mean and median temperature should be reported.
- When a clinical study uses cryopreserved MSCs, MSC conditioning prior to administration (e.g., frozen/thawed/administration or frozen/thawed/cultured/administration) should be described.
- Any functional assay performed on the cell product and its results should be reported.
- Describing whether the MSCs used in the clinical trial are derived from the same batch or different batches is important to report.
- MSC viability assessment prior to administration.
- The type of viability assay used.
- The viability assay results.

#### Rationale

A detailed description of MSC provenance (e.g., tissue of origin, donor characteristics) is important, as it influences MSC characteristics and *in vivo* functionality. Immune compatibility between MSC product and study participants should be extensively documented because the results of autologous and allogeneic clinical trials are conflicting. This reinforces the need for major compatibility assessment between infused MSCs and participant recipient with appropriate laboratory methodologies, which will improve safety and efficacy. In addition, substantial human data are needed to resolve these issues. Each step of cell processing (e.g., extraction, conservation) poses a unique risk profile and can influence cell functional capacity and viability; therefore, providing information on these steps is critical to improve knowledge and develop optimized and safer process workflows. Finally, viability testing is an important step in the quality control process, as cell viability can influence a clinical study’s results (negative results or side effects related to dead cells).

#### MSC culture conditions

In MSC clinical trials, the following details should be reported for the MSC culture conditions:

- The method used to culture the MSCs (e.g., 2D versus 3D culture).
- The concentration of oxygen used for MSC culture (e.g., 5% versus 21%).
- MSC clinical studies using fresh MSCs or cryopreserved MSCs with culture prior to administration should report the level of cell confluence (in %) used to harvest the cells for administration to the patient.
- The culture medium used for MSC culture (e.g., Dulbecco’s Modified Eagle’s Medium, alpha Minimum Essential Medium).
- The medium and reagent catalog numbers.
- The use (or not) of serum for MSC culture.
- The type of serum used.
- The amount (in % of the total amount of culture medium) of serum used.
- The use (or not) of human platelet lysate for MSC culture.
- The amount (in % of the total amount of culture medium) of human platelet lysate used.

#### Rationale

As the process defines the product, a description of the entire manufacturing process (including culture conditions) should be reported. Each parameter of the MSC culture (e.g., oxygen concentration, medium, serum) can impact MSCs’ potency, functional properties and viability. Therefore, these parameters are important to report to allow comparison between studies. In addition, biological adjuvants such as serum or human platelet lysate may have effects on immune compatibility and host response with potential consequences for participants’ safety. Extensively reporting these biological adjuvants and the adverse clinical effects is crucial to develop safer MSC products for clinical use.

## Discussion

To improve transparency, reproducibility and ultimately clinical translation in MSC research, we performed a modified Delphi study to re-establish minimal criteria to define and characterize MSCs and to develop reporting guidelines for clinical studies using MSCs. A total of nine items were selected for the minimal criteria for MSC definition and 33 items were included for the reporting guidelines.

To enhance and build on previous attempts to define MSCs [4,6], we conducted an international collaborative study, including a formal consensus process (Delphi method). This was carried out in association with an “integrated knowledge translation approach” (identification and involvement of end users to support result dissemination and implementation). We adopted this approach when we recruited our steering committee, as extra efforts were made to identify and integrate representative knowledge users (e.g., scientists, trialists, manufacturers, journal editors) into the project from its inception. Thus, the needs and considerations of those with the ability to implement the definition and reporting guidelines were considered during the consensus-building process. This approach helps ensure that the methods used to generate the recommendations resonate with the stakeholders and support high levels of commitment from inception to dissemination [14].

We actively addressed limitations during the course of our consensus process. For instance, we encountered an initially low number of participants in round 1 and recognized that the diversity of our steering committee was limited (North America was well represented, whereas Asia and Africa were under-represented). To address this issue, we asked each steering committee member to suggest five potential participants with expertise in MSCs. We also suggested that these participants should be as diverse as possible (sex, career stage, country, etc.), and we did not review or restrict any recommended participants. Through this effort, we successfully increased the number of participants and improved the diversity of perspectives, ultimately improving the inclusiveness and representativeness of our consensus process. For the future review and update of the MSC definition and reporting guidelines, we plan to expand our global representation by actively recruiting more diverse participants from under-represented regions, such as Asia, Africa and South America. An additional limitation of this study is that we defined MSCs as a clinical entity and not as a specific cell type or mixture of cell types. For example, we did not reach a consensus on the frequency of positive cells for a given marker or on the functional assays required to define MSCs. This in turn prevented us from integrating into the definition our growing understanding of the role of these cells *in vivo*.

Knowledge translation is a critical component of our project given the limited dissemination and implementation achieved by previous attempts to define MSCs, as identified by our scoping review [7]. The steering committee was recruited using an “integrated knowledge translation” approach. Further collaboration with regulatory agencies will support incorporation of the consensus definitions and reporting guidelines into national and international standards for MSC research and clinical applications. This will help to streamline regulatory approvals and ensure consistent quality in MSC products. In addition, involving patients and public representatives in the development and refinement of the MSC guidelines to have their perspectives on MSC safety, efficacy and ethical considerations is crucial for shaping the future of MSC research, including research directions and clinical practices. Finally, there is a need to investigate the ethical, legal and social implications of MSC research and therapy to ensure that the definition and guidelines address these issues comprehensively, promoting responsible and equitable use of MSCs.

During the virtual consensus meeting, dissemination and implementation strategies were discussed with the steering committee. The core set of minimal criteria to define and characterize MSCs and items for reporting guidelines will be accompanied by an Explanation and Elaboration (E&E) document. In the E&E document, for each item, we will provide a detailed rationale for why that item was deemed important to define MSCs. This E&E document will be developed in collaboration with the steering committee (each member will be solicited based on their field of expertise to help draft and review the document). The reporting guidelines and the E&E document will be uploaded to the EQUATOR Network database [18]. Development of training programs and educational material (e.g., workshops, webinars, online courses) will be used to promote adoption of the MSC definition and reporting guidelines.

This work provides the community with a current consensus definition for MSCs and items to report for clinical studies using MSCs. The Delphi method provided a framework to structure our conversation in order to arrive at a consensus definition of MSCs and reporting guidelines. The nature of this consensus-building method was that through anonymous voting, all participants had an equal say in the outcome. Some participants felt strongly that other items should be included in the definition and reporting guidelines (e.g., description of MSCs’ *in vitro* differentiation capacity, population doubling time), but community consensus was not reached in these instances. We anticipate that the research community will treat this as a living definition that will need to be reviewed and revised over time as our use and understanding of MSCs evolve (especially with the rapid development of omics approaches at a single-cell level). Thus, the steering committee agreed during the virtual meeting that we should avoid being too prescriptive and that the definition and reporting guidelines should be treated as minimal criteria. We acknowledge that additional characteristics may be valuable to report over time as the community’s discussion in this area matures with regard to our foundational definition and reporting guidelines. If effectively implemented and widely adopted, we believe the definition and reporting guidelines will improve our baseline understanding of MSCs and will be critical to reproducibility and innovation.

## Conclusions

This international modified Delphi study combined with an integrated knowledge approach aimed to establish minimal criteria to define MSCs and to develop reporting guidelines for MSC clinical studies. Our steering committee, comprising 22 stakeholders from various fields of MSC research, along with 64 additional stakeholders in the first round participated in the Delphi process. Through this collaborative effort, we reached a consensus on nine items to define and characterize MSCs as well as 33 items for the reporting guidelines for MSC clinical studies. Dissemination and implementation of the definition and reporting guidelines within the broader MSC research community will be critical to improve transparency and reproducibility in MSC research.

## Supplementary Material

supp material 6

supp material 5

supp material 3

supp material 2

supp material 1

supp material 4

Supplementary material associated with this article can be found in the online version at doi:10.1016/j.jcyt.2024.10.008.

## Figures and Tables

**Table 1:** Delphi participant demographics.

Participant characteristics	Round 1, n (%) N = 87	Round 2, n (%) N = 17	Invited for round 3, n (%) N = 22	Round 3, day 1, n (%) N = 14^a^	Round 3, day 2, n (%) N = 15^a^

Origin	-				
Africa	2 (2)	-	-	-	-
Asia	7 (8)	-	-	-	-
Australia/Oceania	6 (7)	1 (6)	2 (9)	-	2 (14)
Europe	21 (24)	2 (12)	2 (9)	1 (7)	1 (7)
North America	43 (49)	13 (76)	17 (77)	12 (86)	11 (72)
South America	8 (9)	1 (6)	1 (5)	1 (7)	1 (7)
Sex					
Male	55 (63)	13 (76)	16 (73)	10 (71)	12 (80)
Female	32 (37)	4 (24)	6 (27)	4 (29)	3 (20)
Age					
≤34	4 (5)	-	-	-	-
35–44	22 (25)	1 (6)	3 (14)	3 (24)	1 (7)
45–54	32 (37)	7 (41)	7 (32)	5 (38)	5 (36)
55–64	24 (28)	8 (47)	11 (50)	5 (38)	8 (57)
≥65	5 (6)	1 (6)	1 (5)	-	-
Career stage^b^					
Trainee	6 (7)	-	-	-	-
<5 years	4 (5)	-	1 (5)	1 (8)	1 (8)
5–15 years	30 (36)	4 (25)	5 (24)	4 (33)	2 (15)
>15 years	43 (52)	12 (75)	15 (71)	7 (59)	10 (77)
Are you currently conducting research?					
Yes	82 (94)	16 (94)	21 (95)	12 (92)	13 (93)
Type of research^c^					
Basic	56 (64)	9 (53)	12 (55)	6 (46)	8 (57)
Pre-clinical	59 (68)	11 (65)	14 (64)	8 (62)	10 (71)
Clinical	39 (45)	10 (59)	12 (55)	7 (54)	9 (64)
Methodology	4 (5)	1 (6)	3 (14)	3 (23)	1 (7)
Social science	4 (5)	1 (6)	2 (9)	1 (8)	-
Regulatory science	8 (9)	2 (12)	3 (14)	2 (15)	3 (21)
Bioethics	1 (1)	-	-	-	-
Process development	3 (3)	-	-	-	-
Bioengineering	4 (5)	-	-	-	-
Research area^c^					
Adipose tissue-derived MSCs	5 (6)	-	1 (5)	-	-
Bone marrow-derived MSCs	2 (2)	-	-	-	-
Blood, immune system	31 (36)	6 (35)	9 (41)	4 (31)	7 (50)
Cancer	22 (25)	4 (23)	6 (27)	4 (31)	7 (50)
Cardiovascular system	14 (16)	3 (18)	5 (23)	4 (31)	3 (21)
Cell-based delivery	21 (24)	-	5 (23)	2 (15)	3 (21)
Cell therapy	51 (59)	9 (53)	12 (55)	7 (54)	8 (57)
Digestive system	3 (3)	1 (6)	2 (9)	1 (8)	2 (14)
Ear, nose and throat	4 (5)	1 (6)	2 (9)	1 (8)	2 (14)
Endocrinology	5 (6)	1 (6)	1 (5)	-	1 (7)
MSC-derived extracellular vesicle therapy	10 (11)	-	-	-	-
Gene editing	8 (9)	-	-	-	-
Gene therapy	13 (15)	-	4 (18)	3 (23)	4 (29)
Medical mycology	1 (1)	-	-	-	-
Meta-science	4 (5)	-	3 (14)	2 (15)	-
Musculoskeletal system and connective tissue	24 (28)	4 (23)	4 (18)	3 (23)	2 (14)
Nervous system	13 (15)	1 (6)	2 (9)	1 (8)	2 (14)
Regulatory	9 (19)	2 (12)	3 (14)	2 (15)	1 (7)
Respiratory system	17 (19)	5 (29)	5 (23)	3 (23)	3 (21)
Skin	9 (10)	1 (6)	1 (5)	-	1 (7)
Transplantation	22 (25)	-	2 (9)	1 (8)	2 (14)
Work sector^c^					
University	66 (76)	12 (71)	17 (77)	10 (77)	11 (79)
Academic institution	22 (25)	4 (23)	5 (23)	2 (23)	4 (29)
Hospital	34 (39)	8 (47)	12 (55)	8 (62)	7 (50)
Regulatory agency	4 (5)	2 (12)	2 (9)	1 (8)	1 (7)
Private company	12 (14)	-	1 (5)	-	-
Publishing	1 (1)	1 (6)	1 (5)	1 (8)	1 (7)
Non-profit organization	6 (7)	1 (6)	1 (5)	1 (8)	1 (7)
Involvement with a private company					
Yes, employee	5 (6)	1 (6)	1 (4.5)	-	-
Yes, funding	4 (5)	-	1 (4.5)	-	1 (7)
Yes, other	10 (11)	-	-	-	-
No	68 (78)	16 (94)	20 (91)	13 (100)	13 (93)

a For round 3, one participant reported only country and sex; for the other variables, % calculated for 13 participants for day 1 and 14 participants for day 2.

b For “career stage,” four participants did not respond in round 1, and % calculated for 83 participants; for round 3, two participants did not respond, and % calculated for 12 (day 1) and 13 (day 2) participants.

c For “type of research,” “research area” and “work sector,” participants had the option to select multiple choices and add items; % calculated for 87 participants for round 1, 17 participants for round 2 and 13 (day 1) and 14 (day 2) participants for round 3.

**Table 2:** MSC definition: Delphi voting results by round.

		Round 1		Round 2		Round 3	
				
Number	Items for MSC definition	Scale	n(%)^a^	Scale	n(%)^a^	Scale	n(%)^a^

Terminology							
1	“Mesenchymal stromal cell (MSC)” is an appropriate term to maintain	1–3	5 (7)	1–3	2 (13)		
		4–6	14 (19)	4–6	0 (0)		
		7–9	55 (74)	7–9	**13 (87)**		
2	“Mesenchymal stromal cell” and “mesenchymal stem cell” are interchangeable terms	1–3	40 (50)	1–3	8 (57)	Include	1 (8)
		4–6	18 (22)	4–6	1 (7)	Exclude	**12 (92)**
		7–9	22 (28)	7–9	5 (36)	Abstain	0 (0)
3	Alternative denomination						
	Alternative denomination: vascular maintenance cell^b^			1–3	**16 (94)**		
				4–6	1 (6)		
				7–9	0 (0)		
	Alternative denomination: multipotent stromal cell^b^			1–3	7 (41)	*As item 1 reached consensus for inclusion on round 2, no vote on alternative denominations suggested during round 1*	
				4–6	4 (35)		
				7–9	6 (24)		
	Alternative denomination: fibroblastic stromal cell^b^			1–3	12 (71)		
				4–6	4 (23)		
				7–9	1 (6)		
	Alternative denomination: tissue-derived stromal cell^b^			1–3	11 (65)		
				4–6	4 (23)		
				7–9	2 (12)		
	Alternative denomination: cultured stromal cell (followed by tissue of origin)^b^			1–3	4 (24)		
				4–6	6 (35)		
				7–9	7 (41)		
	Alternative denomination: mesenchymal signaling cell^b^			1–3	13 (76)		
				4–6	2 (12)		
				7–9	2 (12)		
	Alternative denomination: mesenchymal stromal-derived cell^b^			1–3	7 (41)		
				4–6	4 (24)		
				7–9	6 (35)		
Plastic adherence							
4	A description of MSCs’ capacity to adhere to a plastic surface when maintained in standard culture conditions is essential to define them	1–3	12 (17)	1–3	9 (70)	Include	1 (8)
		4–6	15 (22)	4–6	2 (15)	Exclude	8 (61)
		7–9	42 (61)	7–9	2 (15)	Abstain	4 (31)
Cell marker expression							
5	A description of MSCs’ positive and negative markers is essential to define them	1–3	3 (4)				
		4–6	10 (14)				
		7–9	**56 (82)**				
6	For MSC marker expression, the flow cytometry cutoff (% of cells) to consider a cell marker a positive or negative marker should be detailed in the methods section	1–3	5 (8)	1–3	2 (14)	Include	**11 (92)**
		4–6	12 (18)	4–6	4 (29)	Exclude	0 (0)
		7–9	49 (74)	7–9	8 (57)	Abstain	1 (8)
7	For MSC marker expression, the flow cytometry results with the % of positive cells should be described for each positive and negative marker in the results section	1–3	9 (13)	1–3	5 (33)	*Item modified for voting (see 8)*	
		4–6	11 (17)	4–6	4 (27)		
		7–9	46 (70)	7–9	6 (40)		
8^c^	For MSC marker expression, the flow cytometry results with the % of positive cells should be described for each positive and negative marker					Include	**11 (85)**
						Exclude	0 (0)
						Abstain	2 (15)
9	The following positive cell markers are essential to define MSCs						
	CD29+	1–3	13 (26)	1–3	1 (8)	*Item modified for voting (see 10)*	
		4–6	21 (42)	4–6	8 (67)		
		7–9	16 (32)	7–9	3 (25)		
	CD44+	1–3	10 (18)	1–3	2 (17)		
		4–6	17 (32)	4–6	7 (58)		
		7–9	27 (50)	7–9	3 (25)		
	CD73+	1–3	1 (2)				
		4–6	6 (10)				
		7–9	**51 (88)**				
	CD90+	1–3	2 (3)				
		4–6	3 (5)				
		7–9	**54 (92)**				
	CD105+	1–3	3 (5)				
		4–6	7 (13)				
		7–9	**46 (82)**				
	CD166+	1–3	15 (34)	1–3	3 (27)		
		4–6	20 (45)	4–6	7 (64)		
		7–9	9 (21)	7–9	1 (9)		
	CD299+	1–3	17 (50)	1–3	4 (36)		
		4–6	16 (47)	4–6	7 (64)		
		7–9	1 (3)	7–9	0 (0)		
	CD10+^b^			1–3	4 (40)		
				4–6	6 (60)		
				7–9	0 (0)		
	CD140+^b^			1–3	4 (40)		
				4–6	4 (40)		
				7–9	2 (20)		
	CD142+^b^			1–3	5 (50)		
				4–6	4 (40)		
				7–9	1 (10)		
	CD271+^b^			1–3	5 (50)		
				4–6	4 (40)		
				7–9	1 (10)		
	CD276+^b^			1–3	7 (70)		
				4–6	3 (30)		
				7–9	0 (0)		
	HLA-I+^b^			1–3	6 (60)		
				4–6	4 (40)		
				7–9	2 (20)		
	SSEA-3+^b^			1–3	7 (70)		
				4–6	3 (30)		
				7–9	0 (0)		
	SSEA-4+^b^			1–3	**8 (80)**		
				4–6	2 (20)		
				7–9	0 (0)		
	Nestin+^b^			1–3	**9 (82)**		
				4–6	1 (9)		
				7–9	1 (9)		
10^c^	Positive cell markers used to define MSCs should be reported; reliable examples include CD73+, CD90+, CD105+; additional markers used should also be reported					Include	**11 (92)**
						Exclude	0 (0)
						Abstain	1 (8)
11	The following negative cell markers are essential to define MSCs						
	CD3−	1–3	20 (39)	1–3	5 (42)	*Item modified for voting (see 12)*	
		4–6	11 (22)	4–6	3 (25)		
		7–9	20 (39)	7–9	4 (33)		
	CD11−	1–3	13 (26)	1–3	2 (15)		
		4–6	12 (24)	4–6	2 (15)		
		7–9	25 (50)	7–9	9 (70)		
	CD14−	1–3	10 (19)	1–3	2 (15)		
		4–6	8 (15)	4–6	3 (23)		
		7–9	35 (66)	7–9	8 (62)		
	CD19−	1–3	17 (32)	1–3	3 (25)		
		4–6	11 (21)	4–6	2 (17)		
		7–9	25 (47)	7–9	7 (58)		
	CD31−	1–3	12 (24)	1–3	3 (23)		
		4–6	8 (16)	7–9	30 (60)		
		4–6	3 (23)	7–9	7 (54)		
	CD34−	1–3	14 (24)	1–3	3 (22)		
		4–6	7 (12)	4–6	1 (7)		
		7–9	38 (64)	7–9	10 (71)		
	CD45−	1–3	3 (5)				
		4–6	3 (5)				
		7–9	52 (90)				
	HLA DR−	1–3	13 (25)	1–3	3 (21)		
		4–6	7 (14)	4–6	3 (21)		
		7–9	31 (61)	7–9	8 (58)		
	CD11b−	1–3	4 (25)	1–3	0 (0)		
		4–6	5 (31)	4–6	3 (27)		
		7–9	7 (44)	7–9	8 (73)		
12^c^	Negative cell markers used to define MSCs should be reported (CD45- should always be reported); additional tissue-relevant markers used should also be reported					Include	**14 (93)**
						Exclude	0 (0)
						Abstain	1 (7)
Differentiation							
13	A description of MSCs’ in vitro differentiation capacity (e.g., differentiation into adipocytes, chondrocytes) is essential to define them	1–3	21 (31)	1–3	4 (31)	Include	3 (21)
		4–6	13 (19)	4–6	6 (46)	Exclude	9 (65)
		7–9	34 (50)	7–9	3 (23)	Abstain	2 (14)
14	The following differentiation assays are important to define MSCs					*As item 13 was excluded during round 3, no vote on items 14,15 and 16*	
	Trilineage differentiation Adipocyte Osteoblast Chondroblast None of these	41 (50) 8 (10) 6 (7) 4 (5) 23 (28)		6 (38) 4 (25) 1 (6) 0 (0) 5 (31)			
15	MSCs’ in vitro differentiation capacity should be qualitative	1–3	17 (26)	1–3	4 (27)		
		4–6	21 (32)	4–6	3 (20)		
		7–9	28 (42)	7–9	8 (53)		
16	MSCs’ in vitro differentiation capacity should be quantitative	1–3	22 (34)	1–3	8 (53)		
		4–6	19 (29)	4–6	4 (27)		
		7–9	24 (37)	7–9	3 (20)		
Tissue origin							
17	A description of where the MSCs were sourced from is essential to characterize them	1–3	6 (8)			*Item modified for voting (see 19)*	
		4–6	4 (6)				
		7–9	**61 (86)**				
18	The following tissues are a source of MSCs						
	Bone marrow	1–3	1 (1)			*Item modified for voting (see 19)*	
		4–6	0 (0)				
		7–9	**71 (99)**				
	Umbilical cord (Wharton jelly)	1–3	1 (1)				
		4–6	0 (0)				
		7–9	**66 (99)**				
	Adipose tissue	1–3	1 (1)				
		4–6	3 (4)				
		7–9	**68 (95)**				
	Placenta/amnion	1–3	5 (9)				
		4–6	6 (10)				
		7–9	**46 (81)**				
	Umbilical cord blood	1–3	14 (22)	1–3	4 (29)		
		4–6	12 (18)	4–6	1 (7)		
		7–9	39 (60)	7–9	9 (64)		
	Synovial tissue	1–3	8 (17)	1–3	3 (28)		
		4–6	12 (26)	4–6	4 (36)		
		7–9	26 (57)	7–9	4 (36)		
	Peripheral blood	1–3	32 (54)	1–3	11 (74)		
		4–6	12 (20)	4–6	2 (13)		
		7–9	15 (26)	7–9	2 (13)		
	Dental follicle^b^			1–3	1 (10)		
				4–6	1 (10)		
				7–9	**8 (80)**		
	Menstrual blood^b^			1–3	4 (36)		
				4–6	2 (18)		
				7–9	5 (46)		
	iPSC and fetal tissue^b^			1–3	0 (0)		
				4–6	3 (25)		
				7–9	9 (75)		
	Most tissues (excluding central nervous system)^b^			1–3	5 (50)		
				4–6	2 (20)		
				7–9	3 (30)		
	Most tissues (including central nervous system)^b^			1–3	7 (70)		
				4–6	3 (30)		
				7–9	0 (0)		
	Amniotic fluid^b^			1–3	4 (36)		
				4–6	3 (28)		
				7–9	4 (36)		
	Virtually all tissues^b^			1–3	9 (64)		
				4–6	4 (29)		
				7–9	1 (7)		
19^c^	A description of where the MSCs were sourced from is essential to characterize them; any tissue can be noted as the source; no further discussion of tissue source is required					Include	**15 (100)**
						Exclude	0 (0)
						Abstain	0 (0)
Evidence of stemness *in vitro*							
20	A description of self-renewal and multilineage differentiation capacities is essential to define MSCs	1–3	23 (33)	1–3	5 (33)	*Item modified for voting (see 22)*	
		4–6	12 (17)	4–6	8 (54)		
		7–9	35 (50)	7–9	2 (13)		
21	A description of the specific method used to assess MSC stemness *in vitro* is essential to define MSCs	1–3	15 (21)	1–3	4 (25)		
		4–6	16 (23)	4–6	6 (37.5)		
		7–9	39 (56)	7–9	6 (37.5)		
22^c^	If authors use the term “stem” (i.e., mesenchymal stem cell), a description of the methods used to demonstrate stemness must be provided					Include	**13 (93)**
						Exclude	0 (0)
						Abstain	1 (7)
*In vitro* functional assays							
23	A description of *in vitro* functional assays (using quantitative RNA analysis of selected genes, protein analysis of MSC secretome, etc.) to assess MSCs’ potency and properties (e.g., trophic factor secretion, immunomodulation) is essential to characterize MSCs	1–3	10 (15)	1–3	4 (25)	*Item modified for voting (see 24 and 25)*	
		4–6	17 (25)	4–6	2 (12)		
		7–9	41 (60)	7–9	10 (63)		
24^c^	A description of critical quality attributes to assess MSCs’ potency and properties is essential to characterize MSCs for clinical use					Include	**12 (80)**
						Exclude	0 (0)
						Abstain	3 (20)
25^c^	A description of critical quality attributes to assess MSCs’ potency and properties is essential to characterize MSCs for investigative use					Include	7 (54)
						Exclude	4 (31)
						Abstain	2 (15)
MSC licensing							
26	MSC licensing (i.e., pre-conditioned *in vitro* by pro-inflammatory cytokine exposure to mimic *in vivo* inflammatory environment) is essential to characterize MSCs	1–3	25 (42)	1–3	8 (53)	*Item modified for voting (see 27 and 28)*	
		4–6	18 (31)	4–6	5 (33)		
		7–9	16 (27)	7–9	2 (14)		
27^c^	MSC licensing (pre-conditioning) is essential to characterize MSCs					Include	2 (13)
						Exclude	**12 (80)**
						Abstain	1 (7)
28^c^	A statement regarding licensing (pre-conditioning) of MSCs should be included					Include	5 (33)
						Exclude	8 (54)
						Abstain	2 (13)
29	Molecules used for licensing should be described when defining MSCs	1–3	8 (14)	1–3	2 (13)	*As item 27 was excluded during round 3, no vote on items 29 and 30*	
		4–6	11 (18)	4–6	3 (20)		
		7–9	41 (68)	7–9	10 (67)		
30	Resting (non-licensed) MSCs should be used as an internal control when defining MSCs	1–3	11 (20)	1–3	1 (7)		
		4–6	11 (20)	4–6	3 (21)		
		7–9	34 (60)	7–9	10 (72)		
31	Additional characteristics that are essential to define or characterize MSCs						
	Transcriptome analysis (e.g., single-cell RNA sequencing)			1–3	6 (37)	*Item modified for voting (see 32)*	
				4–6	7 (44)		
				7–9	3 (19)		
	Secretome profile			1–3	6 (40)		
				4–6	6 (40)		
				7–9	3 (20)		
	Exosomes (exosome signature, quantitative measurement)			1–3	9 (64)		
				4–6	4 (29)		
				7–9	1 (7)		
	Immunomodulatory and MLR assays			1–3	6 (40)		
				4–6	7 (47)		
				7–9	2 (13)		
	Angiogenic assays			1–3	8 (53)		
				4–6	5 (33)		
				7–9	2 (14)		
	Transcription factor expression (e.g., gene expression analysis for OCT4, SOX2)			1–3	9 (56)		
				4–6	5 (31)		
				7–9	2 (13)		
	DNA methylation profile			1–3	8 (50)		
				4–6	8 (50)		
				7–9	0 (0)		
32^c^	These additional characteristics are not essential to define MSCs					Yes	**14 (93)**
						No	1 (7)
						Abstain	0 (0)

iPSC, induced pluripotent stem cell; MLR, mixed lymphocyte reaction.

a Bold numbers indicate consensus.

b Item introduced during round 1.

C Item introduced during round 3.

**Table 3:** Reporting guidelines: Delphi voting results by round.

		Round 1		Round 2		Round 3	
				
Number	Items for reporting guidelines-Round 1-Round 2-Round 3	Scale	n(%)^a^	Scale	n(%)^a^	Scale	n(%)^a^

MSC intervention and control groups							
	*MSC administration route*						
1	MSC administration route (e.g., intravenous, intra-articular) should be reported	1–3	3 (4)				
		4–6	0 (0)				
		7–9	**68 (96)**				
	*MSC dose*						
2	MSC dose in the intervention group should be reported	1–3	3 (4)				
		4–6	0 (0)				
		7–9	**68 (96)**				
3	The MSC dose should be reported as a dose normalized to weight (number of cells per kilogram of body weight)	1–3	10 (14)	1–3	3 (19)	*Item modified for voting (see 4)*	
		4–6	11 (16)	4–6	4 (25)		
		7–9	50 (70)	7–9	9 (56)		
4^b^	The MSC dose should be reported as a dose normalized to weight (number of cells per kilogram of body weight); if this is not being provided, please explain the rationale					Include	**13 (100)**
						Exclude	0 (0)
						Abstain	0 (0)
	*MSC product*						
5	MSC product concentration (i.e., concentration [number of cells per milliliter of vehicle] of the cell product administered to the patient) should be reported	1–3	2 (3)				
		4–6	7 (10)				
		7–9	**60 (87)**				
6	The vehicle in which MSCs are delivered to the patient should be reported	1–3	5 (7)				
		4–6	0 (0)				
		7–9	**64 (93)**				
	*MSC infusion rate*						
7	MSC clinical studies using intravenous route for MSC administration should report the MSC solution infusion rate	1–3	4 (6)				
		4–6	7 (11)				
		7–9	**54 (83)**				
	*Use of adjuvants for MSC preparation*						
8	The use of adjuvants during the preparation or processing of MSCs (e.g., use of DMSO for MSC preparation) should be reported	1–3	1 (1)				
		4–6	2 (3)				
		7–9	**67 (96)**				
	*Control group*						
9	When the study design involves a control group, the characteristics of the control group should be reported	1–3	1 (1)				
		4–6	0 (0)				
		7–9	**69 (99)**				
10	The type of control used should be reported	1–3	3 (4)				
		4–6	0 (0)				
		7–9	**67 (96)**				
MSC characteristics							
	*MSC provenance*						
11	MSC provenance (e.g., MSC provenance can be from patient, donor or cell from stem cell company) should be reported	1–3	3 (4)				
		4–6	0 (0)				
		7–9	**67 (96)**				
12	MSC donor characteristics (e.g., age, sex, BMI, health status, medication, smoking) should be extensively described^c^			1–3	1 (6)		
				4–6	2 (13)		
				7–9	**13 (81)**		
13	The tissue source of the MSCs (e.g., bone marrow, adipose tissue) should be reported	1–3	4 (6)				
		4–6	0 (0)				
		7–9	**67 (94)**				
14	The extraction procedure used to obtain MSCs from the tissue source (e.g., enzymatic digestion, mechanical) should be reported and described	1–3	6 (9)				
		4–6	3 (4)				
		7–9	**59 (87)**				
	*MSC immune compatibility*					
15	The immune compatibility between MSCs and the patient (e.g., autologous, unmatched allogeneic, matched allogeneic) should be reported	1–3	5 (7)				
		4–6	4 (6)				
		7–9	**60 (87)**				
	*MSC “fitness*”						
16	MSC state prior to administration (e.g., fresh versus cryopreserved)	1–3	3 (4)				
		4–6	3 (4)				
		7–9	**65 (92)**				
17	For studies using cryopreserved MSCs, the number of months the cells were frozen prior to patient administration should be described	1–3	14 (20)	1–3	5 (33)	*Item modified for voting (see 18and 19)*	
		4–6	21 (30)	4–6	3 (20)		
		7–9	35 (50)	7–9	7 (47)		
18^b^	For studies using cryopreserved MSCs, the number of months the cells were frozen prior to patient administration should be described; the range, mean and median number of months should be reported					Include	**13 (93)**
						Exclude	0 (0)
						Abstain	1 (7)
19^b^	For studies using cryopreserved MSCs, the temperature at which the cells were frozen prior to patient administration should be described; the range, mean and median temperature should be reported					Include	**14 (100)**
						Exclude	0 (0)
						Abstain	0 (0)
20	When a clinical study uses cryopreserved MSCs, MSC conditioning prior to administration (e.g., frozen/thawed/administration or frozen/thawed/cultured/administration) should be described	1–3	2 (3)				
		4–6	5 (7)				
		7–9	**62 (90)**				
21	The population doubling time of the MSCs used in the intervention group should be reported	1–3	8 (12)	1–3	5 (33)	Include	6 (43)
		4–6	18 (27)	4–6	3 (20)	Exclude	5 (36)
		7–9	40 (61)	7–9	7 (47)	Abstain	3 (21)
22	Any functional assay performed on the cell product and its results should be reported^c^			1–3	0 (0)		
				4–6	2 (13)		
				7–9	**13 (87)**		
23	Describing whether the MSCs used in the clinical trial are derived from the same batch or different batches is important to report^c^			1–3	2 (12)		
				4–6	0 (0)		
				7–9	**14 (88)**		
	*MSC viability*						
24	MSC viability assessment prior to administration should be reported	1–3	4 (6)				
		4–6	2 (3)				
		7–9	**63 (91)**				
25	The type of viability assay used should be reported	1–3	3 (4)				
		4–6	7 (10)				
		7–9	**59 (86)**				
26	The viability assay results should be reported	1–3	5 (7)				
		4–6	5 (7)				
		7–9	**59 (86)**				
MSC culture conditions							
	*Method of culture*						
27	The method used to culture the MSCs (2D versus 3D culture) should be reported^c^			1–3	2 (12)		
				4–6	0 (0)		
				7–9	**14 (88)**		
	*Oxygen environment*						
28	The level of oxygen used for MSC culture (e.g., 5% versus 21%) should be reported	1–3	4 (6)				
		4–6	7 (11)				
		7–9	**55 (83)**				
	*Cell confluence*						
29	MSC clinical studies using fresh MSCs or cryopreserved MSCs with culture prior to administration should report the level of cell confluence (in %) used to harvest the cells for administration to the patient	1–3	9 (14)	1–3	2 (12)		
		4–6	12 (18)	4–6	1 (8)		
		7–9	44 (68)	7–9	**12 (80)**		
	*Culture medium*						
30	The culture medium used for MSC culture (e.g., DMEM, alpha-MEM) should be reported	1–3	5 (7)	1–3	2 (12)		
		4–6	10 (14)	4–6	0 (0)		
		7–9	55 (79)	7–9	**14 (88)**		
31	The medium and reagent catalog numbers should be reported in the methods section^c^			1–3	2 (12)	Include	**11 (85)**
				4–6	3 (19)	Exclude	0 (0)
				7–9	11 (69)	Abstain	2 (15)
	*Use of serum*						
32	The use (or not) of serum for MSC culture should be reported	1–3	7 (10)				
		4–6	6 (9)				
		7–9	**57 (81)**				
33	The type of serum used should be reported	1–3	3 (4)				
		4–6	8 (11)				
		7–9	**60 (85)**				
34	The amount (in % of the total amount of culture medium) of serum used should be reported	1–3	2 (3)				
		4–6	7 (10)				
		7–9	**59 (87)**				
	*Use of human platelet lysate*						
35	The use (or not) of human platelet lysate for MSC culture should be reported	1–3	4 (6)				
		4–6	5 (8)				
		7–9	**57 (86)**				
36	The amount (in % of the total amount of culture medium) of human platelet lysate used should be reported	1–3	5 (8)				
		4–6	7 (11)				
		7–9	**53 (81)**				

alpha-MEM, alpha Minimum Essential Medium; BMI, body mass index; DMEM, Dulbecco’s Modified Eagle’s Medium; DMSO, dimethyl sulfoxide; 2D, two-dimensional; 3D, three-dimensional.

a Bold numbers indicate consensus.

b Item introduced during round 3.

c Item introduced during round 1.

**Table 4:** Included and excluded items for minimal criteria to define or characterize MSCs.

Number	Items for MSCs definition	Round		
		
		1	2	3

*Terminology*				
1	Mesenchymal Stromal Cell (MSCs) is an appropriate term to maintain		Included	-
2	Mesenchymal Stromal Cell and Mesenchymal Stem Cell are interchangeable terms			Excluded
3	Alternative denomination^a^			Excluded
	Alternative denomination: vascular maintenance cell Alternative denomination: Multipotent stromal cell Alternative denomination: Fibroblastic stromal cell Alternative denomination: Tissue derived stromal cell Alternative denomination: Cultured stromal cell (followed by its tissue of origin) Alternative denomination: Mesenchymal signaling cell Alternative denomination: Mesenchymal stromal derived cell		Excluded	
*Plastic adherence*				
4	A description of MSC capacity to adhere to a plastic surface when maintained in standard culture condition, is essential to define them.			Excluded
*Cell markers expression*				
5	A description of MSC positive and negative markers is essential to define them.	Included		
6	For MSC markers expression, the flow cytometry cut-off (% of cells) to consider a cell marker as a positive or a negative marker should be detailed in the Methods section.			Included
7	For MSC markers expression, the flow cytometry results with the % of positive cells should be described for each positive and negative marker in the Results section.			*Replaced by Item 8*
8	For MSC markers expression, the flow cytometry results with the % of positive cells should be described for each positive and negative marker. ^a^			Included
9	The following positive cell markers essential to define MSCs			
	CD29+			*Replaced by Item 10*
	CD44+			
	CD73+	Included		
	CD90+	Included		
	CD105+	Included		
	CD166+			
	CD299+			
	CD10+^a^			
	CD140+ ^a^			
	CD142+ ^a^			
	CD271+^a^			
	CD276+ ^a^			
	HLA-I+ ^a^			
	SSEA-3+ ^a^			
	SSEA-4+ ^a^		Excluded	
	Nestin+^a^		Excluded	
10	Positive cell markers used to define MSCs should be reported. Reliable examples include CD73+, CD90+, CD105+; additional markers used should also be reported.^b^			Included
11	The following negative cell markers essential to define MSCs			
	CD3−			*Replaced by Item 12*
	CD11−			
	CD14−			
	CD19−			
	CD31−			
	CD34−			
	CD45−	Included		
	HLA DR−			
	CD11b−			
12	Negative cell markers used to define MSCs should be reported (CD45- should always be reported); Additional tissue-relevant markers used should also be reported.^b^			Included
*Differentiation*				
13	A description of MSCs in-vitro differentiation capacity (e.g., differentiation in adipocytes, chondrocytes…etc.) is essential to define them.			Excluded
14	The following differentiation assays are important to define MSCs			Excluded
	Tri-lineage differentiation Adipocyte Osteoblast Chondroblast None of those assays			
15	The MSC in-vitro differentiation capacity should be qualitative.			Excluded
16	The MSC in-vitro differentiation capacity should be quantitative.			Excluded
*Tissue origin*				
17	A description of where the MSCs were sourced from is essential to characterize them	Included		*Replaced by Item 19*
18	The following tissues are a source of MSCs.			
	Bone marrow	Included		*Replaced by Item 19*
	Umbilical cord (Wharton jelly)	Included		
	Adipose tissue	Included		
	Placenta / amnion	Included		
	Umbilical cord blood	Included		
	Synovial			
	Peripheral blood			
	Dental follicle ^a^		Included	
	Menstrual blood ^a^			
	iPSC and fetal tissue ^a^			
	Most tissues (excluding central nervous system) ^a^			
	Most tissues (including central nervous system) ^a^			
	Amniotic fluid ^a^			
	Virtually all the tissues ^a^			Included
19	A description of where the MSCs were sourced from is essential to characterize them. Any tissue can be noted as the source. No further discussion of tissue source is required. ^b^			
*Evidence of stemness in-vitro*				
20	A description of self-renewal and multilineage differentiation capacities is essential to define MSCs.			*Replaced by Item 22*
21	The description of the specific method used to assess MSC stemness in-vitro is essential to define MSCs.			
22	If the author uses the term “stem” (i.e., Mesenchymal Stem Cells) a description of the methods used to demonstrate stemness must be provided.^b^			Included
*In-vitro functional assays*				
23	A description of in-vitro functional assays (using quantitative RNA analysis of selected genes, proteins analysis of MSC secretome…etc.) to assess MSCs’ potency and properties (e.g., trophic factors secretion, immunomodulation…etc.) is essential to characterize MSCs			*Replaced by items 24 & 25*
24	A description of critical quality attributes to assess MSCs’ potency and properties is essential to characterize MSCs for clinical use. ^b^			Included
25	A description of critical quality attributes to assess MSCs’ potency and properties is essential to characterize MSCs for investigative use. ^b^			Excluded
*MSC licensing*				
26	MSC licensing, i.e. preconditioned in-vitro by pro-inflammatory cytokines exposure to mimic in vivo inflammatory environment, is essential to characterize MSCs.			*Replaced by Items 27 & 28*
27	MSC licensing (preconditioning) is essential to characterize MSCs.^b^			Excluded
28	A statement regarding licensing (preconditioning) of MSCs should be included.^b^			Excluded
29	Molecules used for licensing should be described when defining MSCs.			Excluded
30	Resting (non-licensed) MSC should be used as an internal control when defining MSCs.			Excluded
31	Additional characteristics that are essential to define or characterize MSCs			
	Transcriptome analysis (e.g., single-cell RNA sequencing)			*Replaced by Item 32*
	Secretome profile Exosomes (exosome signature, quantitative measurement) Immunomodulatory and MLR assays Angiogenic assays Transcription factors expression (e.g., gene expression analysis for OCT4, SOX2, etc.) DNA methylation profile			
32	These additional characteristics are not essential to define MSCs. ^b^			Included

iPSC, induced pluripotent stem cell; MLR, mixed lymphocyte reaction.

a Item introduced during round 1 (participants’ suggestions).

b Item introduced during round 3 (discussion with steering committee members, rewording of items).

**Table 5:** Included and excluded items for the reporting guidelines for MSC clinical studies.

Number	Items for reporting guidelines	Round		
		
		1	2	3

MSC intervention and control groups *MSC administration route*				
1	MSC administration route (e.g., intravenous, intra-articular) should be reported	Included		
*MSC dose*				
2	MSC dose in the intervention group should be reported	Included		
3	The MSC dose should be reported as a dose normalized to weight (number of cells per kilogram of body weight)			*Replaced by item 4*
4^a^	The MSC dose should be reported as a dose normalized to weight (number of cells per kilogram of body weight); if this is not being provided, please explain the rationale			Included
*MSC product*				
5	MSC product concentration (i.e., concentration [number of cells per milliliter of vehicle] of the cell product administered to the patient) should be reported	Included		
6	The vehicle in which MSCs are delivered to the patient should be reported	Included		
*MSC infusion rate*				
7	MSC clinical studies using intravenous route for MSC administration should report the MSC solution infusion rate	Included		
*Use of adjuvants for MSC preparation*				
8	The use of adjuvants during the preparation or processing of MSCs (e.g., use of DMSO for MSC preparation) should be reported	Included		
*Control group*				
9	When the study design involves a control group, the characteristics of the control group should be reported	Included		
10	The type of control used should be reported MSC characteristics	Included		
*MSC provenance*				
11	MSC provenance (e.g., MSC provenance can be from patient, donor or cell from stem cell company) should be reported	Included		
12^b^	MSC donor characteristics (e.g., age, sex, BMI, health status, medication, smoking) should be extensively described	Included		
13	The tissue source of the MSCs (e.g., bone marrow, adipose tissue) should be reported	Included		
14	The extraction procedure used to obtain MSCs from the tissue source (e.g., enzymatic digestion, mechanical) should be reported and described	Included		
*MSC immune compatibility*				
15	The immune compatibility between MSCs and the patient (e.g., autologous, unmatched allogeneic, matched allogeneic) should be reported	Included		
*MSC “fitness”*				
16	MSC state prior to administration (e.g., fresh versus cryopreserved) should be reported	Included		
17	For studies using cryopreserved MSCs, the number of months the cells were frozen prior to patient administration should be described			*Replaced by items 18 and 19*
18^a^	For studies using cryopreserved MSCs, the number of months the cells were frozen prior to patient administration should be described; the range, mean and median number of months should be reported			Included
19^a^	For studies using cryopreserved MSCs, the temperature at which the cells were frozen prior to patient administration should be described; the range, mean and median temperature should be reported			Included
20	When a clinical study uses cryopreserved MSCs, MSC conditioning prior to administration (e.g., frozen/thawed/administration or frozen/thawed/cultured/administration) should be described	Included		
21	The population doubling time of the MSCs used in the intervention group should be reported			Excluded
22^b^	Any functional assay performed on the cell product and its results should be reported	Included		
23^b^	Describing whether the MSCs used in the clinical trial are derived from the same batch or different batches is important to report		Included	
*MSC viability*				
24	MSC viability assessment prior to administration should be reported	Included		
25	The type of viability assay used should be reported	Included		
26	The viability assay results should be reported	Included		
MSC culture conditions				
*Method of culture*				
27^b^	The method used to culture the MSCs (2D versus 3D culture) should be reported		Included	
*Oxygen environment*				
28	The level of oxygen used for MSC culture (e.g., 5% versus 21%) should be reported	Included		
*Cell confluence*				
29	MSC clinical studies using fresh MSCs or cryopreserved MSCs with culture prior to administration should report the level of cell confluence (in %) used to harvest the cells for administration to the patient		Included	
*Culture medium*				
30	The culture medium used for MSC culture (e.g., DMEM, alpha-MEM) should be reported		Included	
31^b^	The medium and reagent catalog numbers should be reported in the methods section			Included
*Use of serum*				
32	The use (or not) of serum for MSC culture should be reported	Included		
33	The type of serum used should be reported	Included		
34	The amount (in % of the total amount of culture medium) of serum used should be reported	Included		
*Use of human platelet lysate*				
35	The use (or not) of human platelet lysate for MSC culture should be reported	Included		
36	The amount (in % of the total amount of culture medium) of human platelet lysate used should be reported	Included		

alpha-MEM, alpha Minimum Essential Medium; BMI, body mass index; DMEM, Dulbecco’s Modified Eagle’s Medium; DMSO, dimethyl sulfoxide; 2D, two-dimensional; 3D, three-dimensional.

a Item introduced during round 3.

b Item introduced during round 1.

**Table 6:** Minimal criteria to define and characterize MSCs: items in consensus.

Number	Included items	Round of inclusion

Terminology		
1	“Mesenchymal stromal cell (MSC)” is the most appropriate term to be used	2
Cell marker expression		
2	A description of MSCs’ positive and negative markers is essential to define them	1
3	For MSC marker expression, the flow cytometry cutoff (% of cells) to consider a cell marker a positive or negative marker should be detailed in the methods section	3
4	For MSC marker expression, the flow cytometry results with the % of positive cells should be described for each positive and negative marker	3
5	Positive cell markers used to define MSCs should be reported; reliable examples include CD73+, CD90+, CD105+; additional markers used should also be reported	3
6	Negative cell markers used to define MSCs should be reported; a reliable example is CD45-; additional tissue-relevant markers used should also be reported	3
Tissue of origin		
7	A description of where the MSCs were sourced from is essential to characterize them; any tissue can be noted as the source	3
Evidence of stemness		
8	If authors use the term “stem” (i.e., mesenchymal stem cell), a description of the methods used to demonstrate stemness must be Provided	3
*In vitro* functional assays		
9	A description of critical quality attributes to assess MSCs’ potency and properties is essential to characterize MSCs for clinical use	3

**Table 7:** Items included in the reporting guidelines for MSC clinical studies.

Number	Items for reporting guidelines	Round of inclusion

**MSC intervention and control groups**		
*MSC administration route*		
1	MSC administration route (e.g., intravenous, intra-articular) should be reported	1
*MSC dose*		
2	MSC dose in the intervention group should be reported	1
3	The MSC dose should be reported as a dose normalized to weight (number of cells per kilogram of body weight); if this is not being provided, please explain the rationale	3
*MSC product*		
4	MSC product concentration (i.e., concentration [number of cells per milliliter of vehicle] of the cell product administered to the patient) should be reported	1
5	The vehicle in which MSCs are delivered to the patient should be reported	1
*MSC infusion rate*		
6	MSC clinical studies using intravenous route for MSC administration should report the MSC solution infusion rate	1
*Use of adjuvants for MSC preparation*		
7	The use of adjuvants during the preparation or processing of MSCs (e.g., use of DMSO for MSC preparation) should be reported	1
*Control group*		
8	When the study design involves a control group, the characteristics of the control group should be reported	1
9	The type of control used should be reported	1
**MSC characteristics**		
*MSC provenance*		
10	MSC provenance (e.g., MSC provenance can be from patient, donor or cell from stem cell company) should be reported	1
11	MSC donor characteristics (e.g., age, sex, BMI, health status, medication, smoking) should be extensively described	2
12	The tissue source of the MSCs (e.g., bone marrow, adipose tissue) should be reported	1
13	The extraction procedure used to obtain MSCs from the tissue source (e.g., enzymatic digestion, mechanical) should be reported and described	1
*MSC immune compatibility*		
14	The immune compatibility between MSCs and the patient (e.g., autologous, unmatched allogeneic, matched allogeneic) should be reported	1
*MSC “fitness”*		
15	MSC state prior to administration (e.g., fresh versus cryopreserved) should be reported	1
16	For studies using cryopreserved MSCs, the number of months the cells were frozen prior to patient administration should be described; the range, mean and median number of months should be reported	3
17	For studies using cryopreserved MSCs, the temperature at which the cells were frozen prior to patient administration should be described; the range, mean and median temperature should be reported	3
18	When a clinical study uses cryopreserved MSCs, MSC conditioning prior to administration (e.g., frozen/thawed/administration or frozen/thawed/cultured/administration) should be described	1
19	Any functional assay performed on the cell product and its results should be reported	2
20	Describing whether the MSCs used in the clinical trial are derived from the same batch or different batches is important to report	2
*MSC viability*		
21	MSC viability assessment prior to administration should be reported	1
22	The type of viability assay used should be reported	1
23	The viability assay results should be reported	1
**MSC culture conditions**		
*Method of culture*		
24	The method used to culture the MSCs (2D versus 3D culture) should be reported	2
*Oxygen environment*		
25	The level of oxygen used for MSC culture (e.g., 5% versus 21%) should be reported	1
*Cell confluence*		
26	MSC clinical studies using fresh MSCs or cryopreserved MSCs with culture prior to administration should report the level of cell confluence (in %) used to harvest the cells for administration to the patient	2
*Culture medium*		
27	The culture medium used for MSC culture (e.g., DMEM, alpha-MEM) should be reported	2
28	The medium and reagent catalog numbers should be reported in the methods section	3
*Use of serum*		
29	The use (or not) of serum for MSC culture should be reported	1
30	The type of serum used should be reported	1
31	The amount (in % of the total amount of culture medium) of serum used should be reported	1
*Use of human platelet lysate*		
32	The use (or not) of human platelet lysate for MSC culture should be reported	1
33	The amount (in % of the total amount of culture medium) of human platelet lysate used should be reported	1

alpha-MEM, alpha Minimum Essential Medium; BMI, body mass index; DMEM, Dulbecco’s Modified Eagle’s Medium; DMSO, dimethyl sulfoxide; 2D, two-dimensional; 3D, three-dimensional.
