# Supplementary figures and images for: Delphi-driven consensus definition for mesenchymal stromal cells and clinical reporting guidelines for mesenchymal stromal cell-based therapeutics
Source: Cytotherapy. Author manuscript; Available in PMC 2026 Feb 24. (PMC12931451; doi:10.1016/j.jcyt.2024.10.008)

**Supplemental Figure 1. Delphi participants flowchart**

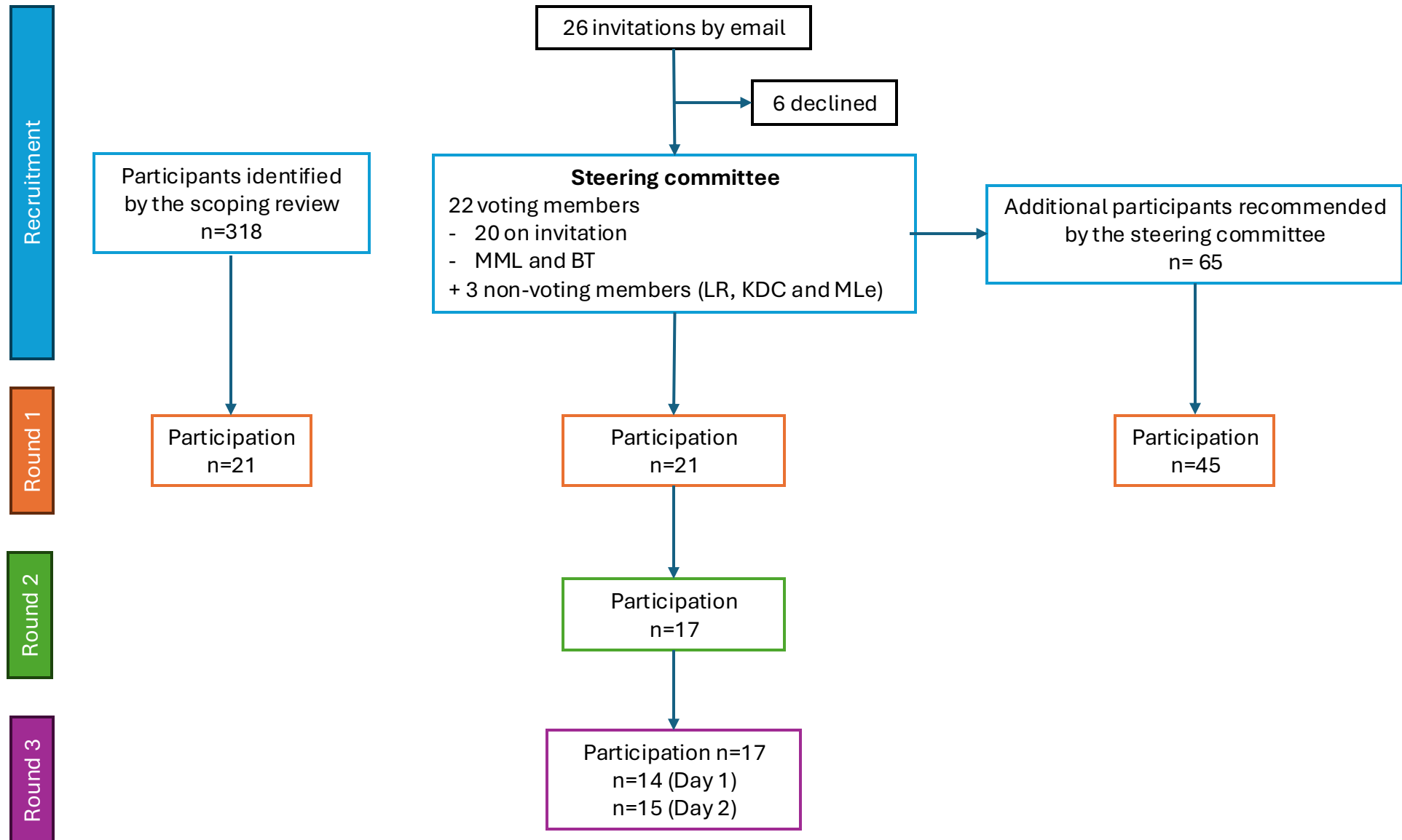

Supplement: supp material 6 [file NIHMS2053365-supplement-supp_material_6.pdf]
