# Supplementary material for: Delphi-driven consensus definition for mesenchymal stromal cells and clinical reporting guidelines for mesenchymal stromal cell-based therapeutics
Source: Cytotherapy. Author manuscript; Available in PMC 2026 Feb 24. (PMC12931451; doi:10.1016/j.jcyt.2024.10.008)
Supplement: supp material 5 [file NIHMS2053365-supplement-supp_material_5.pdf]

Mesenchymal Stromal Cells (MSC) Consensus Meeting

Reporting guidelines

January 09, 2024

The Ottawa Hospital Research Institute | L'Hôpital d'Ottawa Institut de recherche

Inspired by research. **Driven** by compassion. **Inspiré** par la recherche. **Guidé** par la compassion.

Affiliated with Affilié à uOttawa

Welcome

Dr. Bernard Thébaud

The Ottawa Hospital Research Institute | L'Hôpital d'Ottawa Institut de recherche

Inspired by research. **Driven** by compassion.

Inspiré par la recherche. **Guidé** par la compassion.

Affiliated with Affilié à uOttawa

Reporting Guidelines - Agenda

Time (EST)	Activity	Speaker(s)
14:00 – 14:05	Welcome	Bernard Thébaud
14:05 – 14: 10	Summary of Rounds 1 & 2	Laurent Renesme
14:10 – 14:30	Round 3 voting	Manoj Lalu
14:30 – 14:45	Implementation	Kelly Cobey
14:45 – 14:50	Summary Rounds 1 & 2 (definition)	Laurent Renesme
14:50 – 15:55	Round 3 voting (definition)	Manoj Lalu
15:55 – 16:00	Closing remarks	Bernard Thébaud

A modified Delphi study to establish a consensus definition and clinical reporting guidelines for clinical trials for mesenchymal stromal cell (MSC)

Welcome and thank you for taking the time, energy and focus to participate in this virtual event today and tomorrow!

A modified Delphi study to establish a consensus definition and clinical reporting guidelines for clinical trials for mesenchymal stromal cell (MSC)

- The goals of this research project are to
  1. Develop a consensus definition for MSC
  2. Establish reporting guidelines for clinical studies using MSC

- This meeting represents the final round of a 3-round Delphi study

A modified Delphi study to establish a consensus definition and clinical reporting guidelines for clinical trials for mesenchymal stromal cell (MSC)

- Meeting rules
  - We acknowledge this is a controversial topic
  - The goal is to move forward as a community to reach a consensus (each participant will not agree 100% with all items discussed)
  - As everyone voice is important, given the number of items to discuss and the limited time, each intervention must be short.

A modified Delphi study to establish a consensus definition and clinical reporting guidelines for clinical trials for mesenchymal stromal cell (MSC)

- Participants introduction

A modified Delphi study to establish a consensus definition and clinical reporting guidelines for clinical trials for mesenchymal stromal cell (MSC)

- For the reporting guidelines, of 33 items, **29 reached consensus.**
- Today, the focus is to **review 4 items** for the reporting guidelines that have not yet achieved consensus.
- We will have <u>5 minutes to discuss each item</u>
- Dr Kelly Cobey is going to be the time keeping and stepping in to ensure we are hearing from everyone
- Thank you once again for your participation in this important project!

Summary of Delphi Rounds 1 & 2

Dr. Laurent Renesme

The Ottawa Hospital Research Institute | L'Hôpital d'Ottawa Institut de recherche

Inspired by research. Driven by compassion. Inspiré par la recherche. Guidé par la compassion.

Affiliated with Affilié à uOttawa

Participants' demographics

Round #1: n = 87 participants
Round #2: n = 17 participants

Participants characteristics		Round 1 N (%)	Round 2 N (%)
Origin			
	Africa	2 (2)	-
	Asia	7 (8)	-
	Australia / Oceania	6 (7)	1 (6)
	Europe	21 (24)	2 (12)
	North America	43 (49)	13 (76)
	South America	8 (9)	1 (6)
Gender			
	Male	55 (63)	13 (76)
	Female	32 (37)	4 (24)
Age			
	< 34	4 (5)	-
	35 - 44	22 (25)	1 (6)
	45 - 54	32 (37)	7 (41)
	55 - 64	24 (28)	8 (47)
	> 65	5 (6)	1 (6)
Career stage			
	Trainee	6 (7)	-
	Less than 5 years	4 (5)	-
	5 to 15 years	30 (34)	4 (25)
	> 15 years	43 (49)	12 (75)

Participants characteristics		Round 1 N (%)	Round 2 N (%)
Are you currently conducting research?			
	Yes	82 (94)	16 (94)
Type of research			
	Basic research	56 (31)	9 (53)
	Preclinical	59 (33)	11 (65)
	Clinical	39 (22)	10 (59)
	Methodologist	4 (2)	1 (6)
	Social science	4 (2)	1 (6)
	Regulatory science	8 (4)	2 (12)
Research area			
	Blood – Immune system	31 (11)	6 (35)
	Cancer	22 (8)	4 (23)
	Cardiovascular system	14 (5)	3 (18)
	Cell therapy	51 (18)	9 (53)
	Digestive system	3 (1)	1 (6)
	Ear-Nose and Throat (ENT)	4 (1)	1 (6)
	Endocrinology	5 (2)	1 (6)
	Musculoskeletal system	24 (8)	4 (23)
	Nervous system	13 (4)	1 (6)
	Regulatory	9 (3)	2 (12)
	Respiratory system	17 (6)	5 (29)
	Skin	9 (3)	1 (6)

Reporting guidelines – Items in **consensus for inclusion**

**Mesenchymal Stromal cell (MSC) intervention group and control**	
**Item**	**Description**
1	MSC administration route
2	MSC dose in the intervention group
3	MSC product concentration (i.e., concentration of the cell product administered to the patient)
4	The vehicle in which MSC are delivered to the patient
5	MSC solution infusion rate for MSC clinical studies using intra-venous route for MSC administration
6	Use of adjuvants during the preparation or processing of MSC (e.g., use of dimethyl sulfoxide (DMSO) for MSC preparation)
7	Characteristics of the control group when the study design involves a control group
8	The type of control used

Consensus: ≥ 80% of the participants in agreement

Reporting guidelines – Items in **consensus for inclusion**

**MSC characteristics**			
**Item**	**Description**	**Item**	**Description**
9	MSC provenance	21	Method used to culture MSC (2D vs. 3D)
10	MSCs’ Donor characteristics	22	Level of oxygen used for MSC culture
11	The tissue source of the MSC	23	Level of cell confluence
12	The extraction procedure used to obtain MSC from the tissue source	24	Culture medium used
13	Immune compatibility between MSC and patient	25	Use of serum for MSC culture
14	MSC state prior to administration (e.g., Fresh vs. cryopreserved)	26	Type of serum used
15	MSC conditioning prior to administration (if using cryopreserved MSC)	27	Amount of serum used
16	Functional assay performed on MSC product	28	Use of Human platelet lysate
17	Same batch vs. different batches	29	Amount of Human platelet lysate
18	MSC viability assessment		
19	Type of viability assay		
20	Results of viability assay		

Consensus: ≥ 80% of the participants in agreement

Reporting guidelines – Items to continue **to vote on**

Item	Description	Responses	Round 1<br>N (%)	Round 2<br>N (%)
1	The MSC dose should be reported as a **dose normalized to weight** (number of cells per kilogram of bodyweight).	1-3 (Disagree)<br>4-6 (Neutral)<br>7-9 (Agree)	10 (14)<br>11 (16)<br>50 (70)	3 (19)<br>4 (25)<br>9 (56)
2	For studies using **cryopreserved** MSC, the number of months the cells were frozen prior to patient administration should be described.	1-3 (Disagree)<br>4-6 (Neutral)<br>7-9 (Agree)	14 (20)<br>21 (30)<br>35 (50)	5 (33)<br>3 (20)<br>7 (47)
3	The **population doubling time** (PDT) of the MSC used in the intervention group should be reported.	1-3 (Disagree)<br>4-6 (Neutral)<br>7-9 (Agree)	8 (12)<br>18 (27)<br>40 (61)	5 (33)<br>3 (20)<br>7 (47)
4	The media and reagents catalog numbers should be reported in the method section.	1-3 (Disagree)<br>4-6 (Neutral)<br>7-9 (Agree)	-	2 (12)<br>3 (19)<br>11 (69)

Round 3 - Voting

Dr. Manoj Lalu

The Ottawa Hospital Research Institute | L'Hôpital d'Ottawa Institut de recherche

Inspired by research. **Driven** by compassion. **Inspiré** par la recherche. **Guidé** par la compassion.

Affiliated with Affilié à uOttawa

Voting item 1: The MSC dose should be reported as a **dose normalized to weight** (number of cells per kilogram of bodyweight).

Response	Round 1<br>Consensus not achieved		Round 2<br>Consensus not achieved	
	Number	%	Number	%
Disagree (1-3)	10	14	3	19
Neutral (4-6)	11	16	4	25
Agree (7-9)	50	70	9	56
Total	71		16	

Comments Round 2:

- IV doses should be reported as per/Kg body weight . Others can be reported as appropriate.
- This is a standard measure for dosing that is important to allow comparison across studies.
- Dosing is a critical information to facilitate and increase the quality of systematic reviews

VOTING :

The MSC dose should be reported as a **dose normalized to weight** (number of cells per kilogram of bodyweight)

YES

NO

ABSTAIN

Voting item 2: For studies using **cryopreserved MSC**, the number of months the cells were frozen prior to patient administration should be described.

Response	Round 1 Consensus not achieved		Round 2 Consensus not achieved	
	Number	%	Number	%
Disagree (1-3)	14	20	5	33
Neutral (4-6)	21	30	3	20
Agree (7-9)	35	50	7	47
Total	70		15	

Comments Round 2:

- More important than months it is temperature stored / at -150°C cryopreservation is virtually unlimited
- For commercial products it may be difficult to obtain this information
- It is an important information. We do not have sufficient knowledge about this criteria

VOTING :

For studies using **cryopreserved MSC**, the number of months the cells were frozen prior to patient administration should be described.

YES

NO

ABSTAIN

Voting item 3: The **population doubling time** (PDT) of the MSC used in the intervention group should be reported.

Response	Round 1<br>Consensus not achieved		Round 2<br>Consensus not achieved	
	Number	%	Number	%
Disagree (1-3)	8	12	5	33
Neutral (4-6)	18	27	3	20
Agree (7-9)	40	61	7	47
Total	66		15	

Comments Round 2:

- For commercial products it may be difficult to obtain this information
- Specific to the culture conditions used but decreases in PDT over time within a specific culture condition can be an indication of decreases in function
- Critical info that may indicate good growth and correlate with better clinical outcome.

VOTING :

The **population doubling time** (PDT) of the MSC used in the intervention group should be reported.

YES

NO

ABSTAIN

Voting item 4: The media and reagents catalog numbers should be reported in the method section.

Response	Round 1<br>Consensus not achieved		Round 2<br>Consensus not achieved	
	Number	%	Number	%
Disagree (1-3)	-		2	12
Neutral (4-6)	-		3	19
Agree (7-9)	-		11	69
Total			16	

Comments Round 2:

- Developing an efficient methodology is critical and sharing such knowledge is optional and encouraged but not required or mandated.
- For commercial products it may be difficult to obtain this information.
- Materials and methods should be sufficiently detailed to allow replication of the study.

VOTING :

The media and reagents catalog numbers should be reported in the method section.

YES

NO

ABSTAIN

Implementation

Dr. Kelly Cobey

Implementation - Goals

- Dissemination and endorsement of the consensus definition by the research community
  - Fundamental
  - Preclinical
  - Clinical
- Endorsement of the reporting guidelines for MSC clinical research by scientific journals.

Implementation – Plan of action

- Consensus definition
- Reporting guidelines
- Publications
- Registration on Equator Network
- Scientific community needs to facilitate the implementation (Educational material?)
- Frequency for revising the reporting guidelines

Mesenchymal Stromal Cells (MSC) Consensus Meeting

MSC definition

January 10, 2024

The Ottawa Hospital Research Institute | L'Hôpital d'Ottawa Institut de recherche

Inspired by research. **Driven** by compassion. **Inspiré** par la recherche. **Guidé** par la compassion.

Affiliated with Affilié à uOttawa

Welcome

Dr. Bernard Thébaud

MSC Definition- Agenda

Time (EST)	Activity	Speaker(s)
14:00 – 14:05	Welcome	Bernard Thébaud
14:05 – 14: 10	Summary of Rounds 1 & 2	Laurent Renesme
14:10 – 15:45	Round 3 voting	Manoj Lalu
15:45 – 15:55	Implementation	Kelly Cobey
15:55 – 16:00	Closing remarks	Bernard Thébaud

A modified Delphi study to establish a consensus definition and clinical reporting guidelines for clinical trials for mesenchymal stromal cell (MSC)

- For the MSC definition, of 22 items, **6 items reached consensus.**
- Today, the focus is to **review 18 items** for the consensus definition of MSC that have not yet achieved consensus.
- Dr Kelly Cobey is going to be the time keeping and stepping in to ensure we are hearing from everyone
- Thank you once again for your participation in this important project!

Summary of Delphi Rounds 1 & 2

Dr. Laurent Renesme

The Ottawa Hospital Research Institute | L'Hôpital d'Ottawa Institut de recherche

Inspired by research. **Driven** by compassion. **Inspiré** par la recherche. **Guidé** par la compassion.

Affiliated with Affilié à uOttawa

Participants' demographics

Round #1: n = 87 participants
Round #2: n = 17 participants

Participants characteristics		Round 1 N (%)	Round 2 N (%)
Origin			
	Africa	2 (2)	-
	Asia	7 (8)	-
	Australia / Oceania	6 (7)	1 (6)
	Europe	21 (24)	2 (12)
	North America	43 (49)	13 (76)
	South America	8 (9)	1 (6)
Gender			
	Male	55 (63)	13 (76)
	Female	32 (37)	4 (24)
Age			
	< 34	4 (5)	-
	35 - 44	22 (25)	1 (6)
	45 - 54	32 (37)	7 (41)
	55 - 64	24 (28)	8 (47)
	> 65	5 (6)	1 (6)
Career stage			
	Trainee	6 (7)	-
	Less than 5 years	4 (5)	-
	5 to 15 years	30 (34)	4 (25)
	> 15 years	43 (49)	12 (75)

Participants characteristics		Round 1 N (%)	Round 2 N (%)
Are you currently conducting research?			
	Yes	82 (94)	16 (94)
Type of research			
	Basic research	56 (31)	9 (53)
	Preclinical	59 (33)	11 (65)
	Clinical	39 (22)	10 (59)
	Methodologist	4 (2)	1 (6)
	Social science	4 (2)	1 (6)
	Regulatory science	8 (4)	2 (12)
Research area			
	Blood – Immune system	31 (11)	6 (35)
	Cancer	22 (8)	4 (23)
	Cardiovascular system	14 (5)	3 (18)
	Cell therapy	51 (18)	9 (53)
	Digestive system	3 (1)	1 (6)
	Ear-Nose and Throat (ENT)	4 (1)	1 (6)
	Endocrinology	5 (2)	1 (6)
	Musculoskeletal system	24 (8)	4 (23)
	Nervous system	13 (4)	1 (6)
	Regulatory	9 (3)	2 (12)
	Respiratory system	17 (6)	5 (29)
	Skin	9 (3)	1 (6)

Terminology– Items in consensus for inclusion

Terminology	
**Item**	**Description**
1	Mesenchymal Stromal Cell (MSC) is an appropriate term to maintain

Responses	Round 1		Round 2	
	N	%	N	%
Disagree (1-3)	5	7	2	13
Neutral (4-6)	14	19	0	0
Agree (7-9)	55	74	13	87
Total	74		15	

Consensus: ≥ 80% of the participants in agreement

MSC Definition – Items in consensus for inclusion

MSC characteristics	
**Item**	**Description**
2	A description of MSC positive and negative markers is essential to define them.
3	Positive cell markers : CD73+, CD90+, CD105+
4	Negative cell marker: CD45-
5	A description of where the MSC cells were sourced from is essential to characterize them
6	The following tissues are sources of MSC: bone marrow, Umbilical cord, adipose tissue, placenta, Dental follicle

Consensus: ≥ 80% of the participants in agreement

MSC Definition – Items in consensus for exclusion

MSC characteristics	
**Item**	**Description**
1	Positive cell markers : SSEA-4+, Nestin+

Consensus: ≥ 80% of the participants in agreement

Terminology – Items to continue **to vote on**

Item	Description	Responses	Round 1<br>N (%)	Round 2<br>N (%)
1	Mesenchymal Stromal Cell and Mesenchymal Stem Cell are interchangeable terms	1-3 (Disagree)<br>4-6 (Neutral)<br>7-9 (Agree)	40 (50)<br>18 (22)<br>22 (28)	8 (57)<br>1 (7)<br>5 (36)

As the item "Mesenchymal Stromal Cell (MSC) is an appropriate term to maintain" reached consensus for inclusion (87%) , we will not vote on alternative denominations proposed during round 1.

MSC Definition– Items to continue **to vote on**

Item	Description	Responses	Round 1<br>N (%)	Round 2<br>N (%)
2	A description of MSC capacity to adhere to a plastic surface when maintained in standard culture condition, is essential to define them.	1-3 (Disagree)<br>4-6 (Neutral)<br>7-9 (Agree)	12 (17)<br>15 (22)<br>42 (61)	9 (70)<br>2 (15)<br>2 (15)
3	For MSC markers expression, the flow cytometry cut-off (% of cells) to consider a cell marker as a positive or a negative marker should be detailed in the Methods section.	1-3 (Disagree)<br>4-6 (Neutral)<br>7-9 (Agree)	5 (8)<br>12 (18)<br>49 (74)	2 (14)<br>4 (29)<br>8 (57)
4	For MSC markers expression, the flow cytometry results with the % of positive cells should be described for each positive and negative marker in the Results section.	1-3 (Disagree)<br>4-6 (Neutral)<br>7-9 (Agree)	9 (13)<br>11 (17)<br>46 (70)	5 (33)<br>4 (27)<br>6 (40)
5	The following positive cell markers essential to define MSC: CD29+, CD44+, CD166+, CD299+,CD10+, CD140+, CD142, CD271+, CD276+, HLA-I+, SSEA-3+			
6	The following negative cell markers essential to define MSC: CD3-, CD11-, CD14-, CD19-, CD31-, CD34-, HLA-DR-, CD11b-			
7	A description of MSC in-vitro differentiation capacity (e.g., differentiation in adipocytes, chondrocytes...etc.) is essential to define them.	1-3 (Disagree)<br>4-6 (Neutral)<br>7-9 (Agree)	21 (31)<br>13 (19)<br>34 (50)	4 (31)<br>6 (46)<br>3 (23)
8	The following differentiation assays are important to define MSC: Tri-lineage, adipocyte, osteoblast, chondroblast or none of those assays			
9	The MSC in-vitro differentiation capacity should be qualitative.	1-3 (Disagree)<br>4-6 (Neutral)<br>7-9 (Agree)	17 (26)<br>21 (32)<br>28 (42)	4 (27)<br>3 (20)<br>8 (53)
10	The MSC in-vitro differentiation capacity should be quantitative.	1-3 (Disagree)<br>4-6 (Neutral)<br>7-9 (Agree)	22 (34)<br>19 (29)<br>24 (37)	8 (53)<br>4 (27)<br>3 (20)

MSC Definition– Items to continue **to vote on**

Item	Description	Responses	Round 1 N (%)	Round 2 N (%)
11	The following tissues are a source of MSC: Umbilical cord blood, synovial, peripheral blood, menstrual blood, iPSC and fetal tissue, Most tissues, amniotic fluid, virtually all the tissues			
12	A description of self-renewal and multilineage differentiation capacities is essential to define MSC.	1-3 (Disagree)<br>4-6 (Neutral)<br>7-9 (Agree)	23 (33)<br>12 (17)<br>35 (50)	5 (33)<br>8 (54)<br>2 (13)
13	The description of the specific method used to assess MSC stemness in-vitro is essential to define MSC.	1-3 (Disagree)<br>4-6 (Neutral)<br>7-9 (Agree)	15 (21)<br>16 (23)<br>39 (56)	4 (25)<br>6 (37.5)<br>6 (37.5)
14	A description of in-vitro functional assays (using quantitative RNA analysis of selected genes, proteins analysis of MSC secretome...etc.) to assess MSCs' potency and properties (e.g., trophic factors secretion, immunomodulation...etc.) is essential to characterize MSC.	1-3 (Disagree)<br>4-6 (Neutral)<br>7-9 (Agree)	10 (15)<br>17 (25)<br>41 (60)	4 (25)<br>2 (12)<br>10 (63)
15	MSC licensing, i.e. preconditioned in-vitro by pro-inflammatory cytokines exposure to mimic in vivo inflammatory environment, is essential to characterize MSC.	1-3 (Disagree)<br>4-6 (Neutral)<br>7-9 (Agree)	25 (42)<br>18 (31)<br>16 (27)	8 (53)<br>5 (33)<br>2 (14)
16	Molecules used for licensing should be described when defining MSC.	1-3 (Disagree)<br>4-6 (Neutral)<br>7-9 (Agree)	8 (14)<br>11 (18)<br>41 (68)	2 (13)<br>3 (20)<br>10 (67)
17	Resting (non-licensed) MSC should be used as an internal control when defining MSC.	1-3 (Disagree)<br>4-6 (Neutral)<br>7-9 (Agree)	11 (20)<br>11 (20)<br>34 (60)	1 (7)<br>3 (21)<br>10 (72)
18	Additional characteristics that are essential to define or characterize MSC: transcriptome analysis, secretome analysis, exosomes, immunomodulatory and MLR assays, angiogenic assays, transcription factors expression, DNA methylation profile.			

Round 3 - Voting

Dr. Manoj Lalu

The Ottawa Hospital | L'Hôpital d'Ottawa
Research Institute | Institut de recherche

Inspired by research. **Driven** by compassion. **Inspiré** par la recherche. **Guidé** par la compassion.

Affiliated with Affilié à uOttawa

Voting item 1: Mesenchymal Stromal Cell and Mesenchymal Stem Cell are interchangeable terms

Response	Round 1<br>Consensus not achieved		Round 2<br>Consensus not achieved	
	Number	%	Number	%
Disagree (1-3)	40	50	8	57
Neutral (4-6)	18	22	1	7
Agree (7-9)	22	28	5	36
Total	80		14	

Comments Round 2:

- One is based on origin, one on properties
- Stromal cells do not demonstrate stemness properties
- M stem cells are only a fraction of M stromal cells
- Cells described in the literature as MSC, whether they are termed mesenchymal stromal cells, multipotent stromal cells, mesenchymal stem cells or any other combination of words are required to meet the basic definitions set out by ISCT
- Clarity matters, especially in this controversial field of MSC biology. Stem should only refer to cells with proved stemness properties (I.e., self-renewal and multipotentiality)

VOTING :

Mesenchymal Stromal Cell and Mesenchymal Stem Cell are interchangeable terms

YES

NO

ABSTAIN

Voting item 2: A description of MSC capacity to adhere to a plastic surface when maintained in standard culture condition, is essential to define them.

Response	Round 1 Consensus not achieved		Round 2 Consensus not achieved		
	Number	%	Number	%	Comments
Disagree (1-3)	12	17	9	70	• Substrate adherence is a feature of many cell types so is not essential. Since it was first used to enrich cells from bone marrow, it was originally described as a defining feature, but is no longer relevant.<br>• This is not true and strictly only works if you use serum containing medium and does not work well with anything other than bone marrow and even there, macrophages are an issue
Neutral (4-6)	15	22	2	15	• Relevant only for the isolation, since expansion can be done by very different ways<br>• Probably quite helpful as an initial criterion but should not be a single defining requirement.
Agree (7-9)	42	61	2	15	• A caveat is the observation that Both adherent and non-adherent MSCs have been shown to have therapeutic benefits. We may lose a population of putative therapeutic MSCs by disregarding non-adherent cells. Plastic adherence may serve as a factor for primary selection, but not required during further processing of the cells?<br>• Plastic adherence is critical to the purification process used to isolate MSC. New methods of purification and growth may be acceptable if the resulting cell population has been sufficiently demonstrated to behave identically to MSC that were isolated and propagated through plastic adherence.
Total	69		13		

VOTING :

A description of MSC capacity to adhere to a plastic surface when maintained in standard culture condition, is essential to define them

YES

NO

ABSTAIN

Voting item 3: For MSC markers expression, the flow cytometry cut-off (% of cells) to consider a cell marker as a positive or a negative marker should be detailed in the Methods section.

Response	Round 1<br>Consensus not achieved		Round 2<br>Consensus not achieved		
	Number	%	Number	%	Comments
Disagree (1-3)	5	8	2	14	• Setting a threshold would only be useful if the markers used were specific to a cell type or functional status, and none of the currently used markers are.
Neutral (4-6)	12	18	4	29	• A rigorous method of reporting should include gating strategies and representative dot plots, which if provided should provide enough information to access purity and quality of the cell population.
Agree (7-9)	49	74	8	57	• The field has obviously not advanced enough to define MSC by specific markers. However, it may be better to keep these markers for now and acknowledge that these will be finetuned over time as we gain more knowledge<br>• As a standard rule, the methods used for all research should be sufficiently described to allow repetition of the work.<br>• The suggestion is NOT to set a threshold - -it is simply to report the threshold used!
Total	66		14		

VOTING :

For MSC markers expression, the flow cytometry cut-off (% of cells) to consider a cell marker as a positive or a negative marker should be detailed in the Methods section.

YES

NO

ABSTAIN

Voting item 4: For MSC markers expression, the flow cytometry results with the % of positive cells should be described for each positive and negative marker in the Results section.

Response	Round 1 Consensus not achieved		Round 2 Consensus not achieved		
	Number	%	Number	%	Comments
Disagree (1-3)	9	13	5	33	• The description in the Methods section is sufficient.
Neutral (4-6)	11	17	4	27	• I'm neutral on this. These days with high-dimensional spectral flow cytometry being simple to execute robustly, we should he moving to full flow cytometric characterisation of any product and reporting the molecular phenotypes of all populations present. The approach of expressing % positive or negative for single markers relates back to a time when flow cytometry applied to MSC was relatively primitive - not appropriate now.
Agree (7-9)	46	70	6	40	• For most people MSC are a mixed population (non-homogeneous) so percentage expression I think is important
Total	66		15		

VOTING :

For MSC markers expression, the flow cytometry results with the % of positive cells should be described for each positive and negative marker in the Results section.

YES

NO

ABSTAIN

Voting item 5:

The following positive cell markers essential to define MSC.

The following positive cell markers essential to define MSC	Round 1		Round 2	
	Scale	N (%)	Scale	N (%)
CD29+	1 – 3<br>4 – 6<br>7 – 9	13 (26)<br>21 (42)<br>16 (32)	1 – 3<br>4 – 6<br>7 – 9	1 (8)<br>8 (67)<br>3 (25)
CD44+	1 – 3<br>4 – 6<br>7 – 9	10 (18)<br>17 (32)<br>27 (50)	1 – 3<br>4 – 6<br>7 – 9	2 (17)<br>7 (58)<br>3 (25)
CD166+	1 – 3<br>4 – 6<br>7 – 9	15 (34)<br>20 (45)<br>9 (21)	1 – 3<br>4 – 6<br>7 – 9	3 (27)<br>7 (64)<br>1 (9)
CD299+	1 – 3<br>4 – 6<br>7 – 9	17 (50)<br>16 (47)<br>1 (3)	1 – 3<br>4 – 6<br>7 – 9	4 (36)<br>7 (64)<br>0
CD10+[b]			1 – 3<br>4 – 6<br>7 – 9	4 (40)<br>6 (60)<br>0
CD140+[b]			1 – 3<br>4 – 6<br>7 – 9	4 (40)<br>4 (40)<br>2 (20)
CD142+[b]			1 – 3<br>4 – 6<br>7 – 9	5 (50)<br>4 (40)<br>1 (10)
CD271+[b]			1 – 3<br>4 – 6<br>7 – 9	5 (50)<br>4 (40)<br>1 (10)
CD276+[b]			1 – 3<br>4 – 6<br>7 – 9	7 (70)<br>3 (30)<br>0
HLA-I+[b]			1 – 3<br>4 – 6<br>7 – 9	6 (60)<br>4 (40)<br>2 (20)
SSEA-3+[b]			1 – 3<br>4 – 6<br>7 – 9	7 (70)<br>3 (30)<br>0

Positive Cell markers that reached consensus:

- CD73+
- CD90+
- CD105+

VOTING :

For each following positive cell markers, vote if they are essential to define MSC.

YES

NO

ABSTAIN

Voting item 6:

The following negative cell markers essential to define MSC.

Negative Cell markers that reached consensus:

- CD45-

The following negative cell markers essential to define MSC	Round 1		Round 2	
	Scale	N (%)	Scale	N (%)
CD3-	1 – 3<br>4 – 6<br>7 – 9	20 (39)<br>11 (22)<br>20 (39)	1 – 3<br>4 – 6<br>7 – 9	5 (42)<br>3 (25)<br>4 (33)
CD11-	1 – 3<br>4 – 6<br>7 – 9	13 (26)<br>12 (24)<br>25 (50)	1 – 3<br>4 – 6<br>7 – 9	2 (15)<br>2 (15)<br>9 (70)
CD14-	1 – 3<br>4 – 6<br>7 – 9	10 (19)<br>8 (15)<br>35 (66)	1 – 3<br>4 – 6<br>7 – 9	2 (15)<br>3 (23)<br>8 (62)
CD19-	1 – 3<br>4 – 6<br>7 – 9	17 (32)<br>11 (21)<br>25 (47)	1 – 3<br>4 – 6<br>7 – 9	3 (25)<br>2 (17)<br>7 (58)
CD31-	1 – 3<br>4 – 6<br>7 – 9	12 (24)<br>8 (16)<br>30 (60)	1 – 3<br>4 – 6<br>7 – 9	3 (23)<br>3 (23)<br>7 (54)
CD34-	1 – 3<br>4 – 6<br>7 – 9	14 (24)<br>7 (12)<br>38 (64)	1 – 3<br>4 – 6<br>7 – 9	3 (22)<br>1 (7)<br>10 (71)
HLA DR-	1 – 3<br>4 – 6<br>7 – 9	13 (25)<br>7 (14)<br>31 (61)	1 – 3<br>4 – 6<br>7 – 9	3 (21)<br>3 (21)<br>8 (58)
CD11b-	1 – 3<br>4 – 6<br>7 – 9	4 (25)<br>5 (31)<br>7 (44)	1 – 3<br>4 – 6<br>7 – 9	0<br>3 (27)<br>8 (73)

VOTING :

For each following negative cell markers, vote if they are essential to define MSC.

YES

NO

ABSTAIN

Voting item 7: A description of MSC *in-vitro* differentiation capacity (e.g., differentiation in adipocytes, chondrocytes...etc.) is essential to define them.

Response	Round 1 Consensus not achieved		Round 2 Consensus not achieved		
	Number	%	Number	%	Comments
Disagree (1-3)	21	31	4	31	• It is only useful if people want to compare two cell types. It does not establish any functional identity or an obligatory critical aspect of their biology. Cell division is a more important criteria for example.
Neutral (4-6)	13	19	6	46	• Differentiation capacity might be of interest in a given indication. However, in most applications its rather immunomodulation than direct regeneration.<br>• This provides a measure of overall cell fitness and stem/progenitor activity and thus, for now, best marker of therapeutic potential.
Agree (7-9)	34	50	3	23	• The idea that multi-potency is not a main feature of MSCs is unsubstantiated since MSCs were distinguished from tissue fibroblasts by their tri-lineage potential, and all MSC populations described to date exhibit some capacity for connective tissue lineage differentiation.<br>• Yes, differentiation potential is important to define cells with different properties from fibroblasts. But while adipogenic potential is easy to routinely assay using several different methods that correlate well, current assays for chondrogenic and osteogenic assays are not technically robust.<br>• Unless we are changing the ISCT definition, tri-lineage differentiation must remain as a defining element. Adipocyte, chondrocyte and osteocyte differentiation assays have been in place and successfully used for decades and cannot be considered immature.
Total	68		13		

VOTING :

A description of MSC *in-vitro* differentiation capacity (e.g., differentiation in adipocytes, chondrocytes...etc.) is essential to define them.

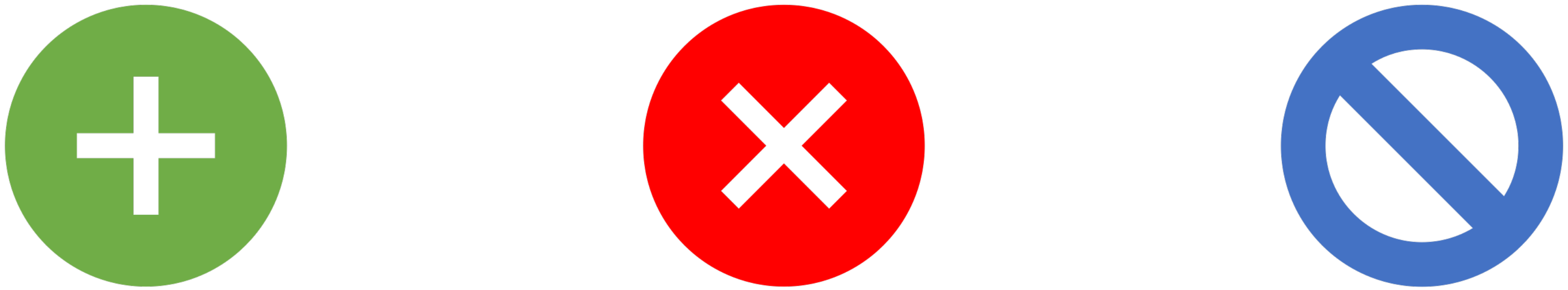

YES **NO** **ABSTAIN**

Voting item 8: The following differentiation assays are important to define MSC.

Differentiation assays	Round 1		Round 2	
	N	%	N	%
Tri-lineage differentiation	41	50	6	38
Adipocyte	8	10	4	25
Osteoblast	6	7	1	6
Chondroblast	4	5	0	0
None of those assays	23	28	5	31
	Total = 82		Total = 16	

VOTING :

The following differentiation assays are important to define MSC: Tri-lineage / Adipocyte / Osteoblast / Chondroblast

YES

NO

ABSTAIN

Voting item 9: The MSC *in-vitro* differentiation capacity should be qualitative.

Response	Round 1 Consensus not achieved		Round 2 Consensus not achieved		
	Number	%	Number	%	Comments
Disagree (1-3)	17	26	4	27	• Qualitative assays are low stringency and provide little useful information for assessing overall cell fitness. Differences in tri-lineage capacity is an important distinguishing characteristic for MSCs derived from different sources and also reflects differences in proliferative capacity, etc.
Neutral (4-6)	21	32	3	20	
Agree (7-9)	28	42	8	53	• MSCs from different sources and/or produced by different processes do not necessarily differentiate to the same extent into each lineage, but they should differentiate into each lineage to some extent. Consequently, a qualitative rather than quantitate assay is appropriate.
Total	66		15		

VOTING :

The MSC *in-vitro* differentiation capacity should be qualitative.

YES

NO

ABSTAIN

Voting item 10: The MSC *in-vitro* differentiation capacity should be quantitative.

Response	Round 1 Consensus not achieved		Round 2 Consensus not achieved		
	Number	%	Number	%	Comments
Disagree (1-3)	22	34	8	53	• quantification is quite difficult and will be very hard to standardize
Neutral (4-6)	19	29	4	27	
Agree (7-9)	24	37	3	20	
Total	65		15		

VOTING :

The MSC *in-vitro* differentiation capacity should be quantitative.

YES

NO

ABSTAIN

Voting item 11:
The following tissues are a source of MSC.

The following tissues are a source of MSC.	Round 1		Round 2	
	Scale	N (%) [a]	Scale	N (%) [a]
Umbilical cord blood	1 – 3<br>4 – 6<br>7 – 9	14 (22)<br>12 (18)<br>39 (60)	1 – 3<br>4 – 6<br>7 – 9	4 (29)<br>1 (7)<br>9 (64)
Synovial	1 – 3<br>4 – 6<br>7 – 9	8 (17)<br>12 (26)<br>26 (57)	1 – 3<br>4 – 6<br>7 – 9	3 (28)<br>4 (36)<br>4 (36)
Peripheral blood	1 – 3<br>4 – 6<br>7 – 9	32 (54)<br>12 (20)<br>15 (26)	1 – 3<br>4 – 6<br>7 – 9	11 (74)<br>2 (13)<br>2 (13)
Menstrual blood [b]			1 – 3<br>4 – 6<br>7 – 9	4 (36)<br>2 (18)<br>5 (46)
iPSC and fetal tissue [b]			1 – 3<br>4 – 6<br>7 – 9	0<br>3 (25)<br>9 (75)
Most tissues (excluding central nervous system) [b]			1 – 3<br>4 – 6<br>7 – 9	5 (50)<br>2 (20)<br>3 (30)
Most tissues (including central nervous system) [b]			1 – 3<br>4 – 6<br>7 – 9	7 (70)<br>3 (30)<br>0
Amniotic fluid [b]			1 – 3<br>4 – 6<br>7 – 9	4 (36)<br>3 (28)<br>4 (36)
Virtually all the tissues [b]			1 – 3<br>4 – 6<br>7 – 9	9 (64)<br>4 (29)<br>1 (7)

Tissue sources that reached consensus:

- Bone marrow
- Umbilical cord
- Adipose tissue
- Placenta
- Dental follicle

Items suggested by participants during 1st round

VOTING :

The following tissues are a source of MSC: Umbilical cord blood, synovial, peripheral blood, menstrual blood, iPSC and fetal tissue, Most tissues (CNS included or excluded), amniotic fluid, virtually all the tissues

YES | **NO** | **ABSTAIN**

Voting item 12: A description of self-renewal and multilineage differentiation capacities is essential to define MSC.

Response	Round 1<br>Consensus not achieved		Round 2<br>Consensus not achieved		
	Number	%	Number	%	Comments
Disagree (1-3)	23	33	5	33	• Unnecessary if we consider that MSC are stromal cells (I.e., no stemness properties).<br>• Self-renewal capacity is not necessary to characterize unless claims are being made on the stemness of the cells; however, proliferation is important to evaluate especially when characterizing new culture conditions as declining proliferation rates can be an indication of functional changes.
Neutral (4-6)	12	17	8	54	• This is the nub of the problem: we do need to distinguish between cells with these properties - stem cells - and other cells that don't have these properties. The stem cell nomenclature and confusion about what it means, is driving most of the inappropriate use of MSC preparations.<br>• Can be useful to assess the cell function (If you want to claim they have stem/progenitor properties) but not to define them
Agree (7-9)	35	50	2	13	• Agree if trying to show stemness properties of mesenchymal stem cells.<br>• Stromal vs. stem is an arbitrary distinction. The best possible definition of MSCs is to validate as many characteristics as possible.
Total	70		15		

VOTING :

A description of self-renewal and multilineage differentiation capacities is essential to define MSC.

YES

NO

ABSTAIN

Voting item 13: The description of the specific method used to assess MSC stemness *in-vitro* is essential to define MSC.

Response	Round 1 Consensus not achieved		Round 2 Consensus not achieved		
	Number	%	Number	%	Comments
Disagree (1-3)	15	21	4	25	• Not essential if not claiming stemness!
Neutral (4-6)	16	23	6	37.5	• Again, depends on definition of S - hence scored neutral. Any claims of stem cell properties do need to be described in a way that enables replication.
Agree (7-9)	39	56	6	37.5	• If claiming stem cell properties, this would be essential; if not, no methodology is needed. Should be part of the definition with this additional explanation.
Total	70		16		

VOTING :

The description of the specific method used to assess MSC stemness *in-vitro* is essential to define MSC.

YES

NO

ABSTAIN

Voting item 14: A description of in-vitro functional assays (using quantitative RNA analysis of selected genes, proteins analysis of MSC secretome...etc.) to assess MSCs' potency and properties (e.g., trophic factors secretion, immunomodulation...etc.) is essential to characterize MSC.

Response	Round 1 Consensus not achieved		Round 2 Consensus not achieved		
	Number	%	Number	%	Comments
Disagree (1-3)	10	15	4	25	• Nice to have but currently we don't know exactly what transcriptome, gene expression profile etc. should be seen in a definitive population.<br>• The only function currently used to define MSC is tri-lineage differentiation. These assays should be accurately described. Other types of assays could be important for characterization but are not yet functional characteristics that need to be applied across all MSC as a definition criteria.
Neutral (4-6)	17	25	2	12	• Just as for molecular phenotype, characterising functional properties of the (usually mixed) populations present in a preparation is useful in comparing potential utility in medicine, and should be encouraged.<br>• MSC stromal properties are highly regulated processes, and many secreted factors can be induced/repressed by biologics, culture conditions, etc. Therefore, stipulating these as defining criteria is unwise without strictly defining the assay parameters under which a given factor should be measured.
Agree (7-9)	41	60	10	63	• At least some potency must be demonstrated (differentiation, immunomodulation, etc.).<br>• Not required to characterize them but essential when you use them for a particular function and to allow one to compare data between labs or confirm a hypothesis.
Total	68		16		

VOTING :

A description of in-vitro functional assays (using quantitative RNA analysis of selected genes, proteins analysis of MSC secretome...etc.) to assess MSCs' potency and properties (e.g., trophic factors secretion, immunomodulation...etc.) is essential to characterize MSC.

YES

NO

ABSTAIN

Voting item 15: MSC licensing, i.e. preconditioned in-vitro by pro-inflammatory cytokines exposure to mimic in vivo inflammatory environment, is essential to characterize MSC.

Response	Round 1<br>Consensus not achieved		Round 2<br>Consensus not achieved		
	Number	%	Number	%	Comments
Disagree (1-3)	25	42	8	53	• Like all others for a cell if that is the function and mechanism of action you propose then you need to show it. If that is not then you don't.
Neutral (4-6)	18	31	5	33	• No use to define MSC but can be of interest to characterize MSC for applications where the proposed mechanism of action relates to inflammatory responses.
Agree (7-9)	16	27	2	14	
Total	59		15		

VOTING :

MSC licensing, i.e. preconditioned in-vitro by pro-inflammatory cytokines exposure to mimic in vivo inflammatory environment, is essential to characterize MSC.

YES

NO

ABSTAIN

Voting item 16: Molecules used for licensing should be described when defining MSC.

Response	Round 1<br>Consensus not achieved		Round 2<br>Consensus not achieved		
	Number	%	Number	%	Comments
Disagree (1-3)	8	14	2	13	
Neutral (4-6)	11	18	3	20	• Not when defining MSC but as reasonable details in describing a reproducible experiment.<br>• Culture conditions need to be described in detail. So, if authors add licensing factors it needs to be reported.
Agree (7-9)	41	68	10	67	
Total	60		15		

VOTING :

Molecules used for licensing should be described when defining MSC.

YES

NO

ABSTAIN

Voting item 17: Resting (non-licensed) MSC should be used as an internal control when defining MSC.

Response	Round 1<br>Consensus not achieved		Round 2<br>Consensus not achieved		
	Number	%	Number	%	Comments
Disagree (1-3)	11	20	1	7	
Neutral (4-6)	11	20	3	21	
Agree (7-9)	34	60	10	72	• MSC is an evolving definition, so the basic MSC is required to make progress towards a better definition (including licensed and other methods to improve MSCs).<br>• the principle of using an internal control as a comparator to characterize newly developed MSC is essential to demonstrate comparability across cell lines.
Total	56		14		

VOTING :

Resting (non-licensed) MSC should be used as an internal control when defining MSC.

YES

NO

ABSTAIN

Voting item 18: Additional characteristics that are essential to define or characterize MSC.

Items suggested by participants during 1st round

Additional characteristics that are essential to define or characterize MSC	Round 1		Round 2	
	Scale	N (%) [a]	Scale	N (%) [a]
Transcriptome analysis (e.g., single-cell RNA sequencing)			1 – 3<br>4 – 6<br>7 – 9	6 (37)<br>7 (44)<br>3 (19)
Secretome profile			1 – 3<br>4 – 6<br>7 – 9	6 (40)<br>6 (40)<br>3 (20)
Exosomes (exosome signature, quantitative measurement)			1 – 3<br>4 – 6<br>7 – 9	9 (64)<br>4 (29)<br>1 (7)
Immunomodulatory and MLR assays			1 – 3<br>4 – 6<br>7 – 9	6 (40)<br>7 (47)<br>2 (13)
Angiogenic assays			1 – 3<br>4 – 6<br>7 – 9	8 (53)<br>5 (33)<br>2 (14)
Transcription factors expression (e.g., gene expression analysis for OCT4, SOX2, etc.)			1 – 3<br>4 – 6<br>7 – 9	9 (56)<br>5 (31)<br>2 (13)
DNA methylation profile			1 – 3<br>4 – 6<br>7 – 9	8 (50)<br>8 (50)<br>0

VOTING :

Additional characteristics that are essential to define or characterize MSC:

- Transcriptome analysis
- Secretome analysis
- Exosomes
- Immunomodulatory and MLR assays
- Angiogenic assays
- Transcription factors expression
- DNA methylation profile

YES **NO** **ABSTAIN**

Further feedback?

Implementation

Dr. Kelly Cobey

The Ottawa Hospital Research Institute | L'Hôpital d'Ottawa Institut de recherche

Inspired by research. **Driven** by compassion. **Inspiré** par la recherche. **Guidé** par la compassion.

Affiliated with Affilié à uOttawa

Implementation - Goals

- Dissemination and endorsement of the consensus definition by the research community
  - Fundamental
  - Preclinical
  - Clinical
- Endorsement of the reporting guidelines for MSC clinical research by scientific journals.

Implementation – Plan of action

- Consensus definition
- Reporting guidelines
- Publications
- Registration on Equator Network
- Scientific community needs to facilitate the implementation (Educational material?)
- Frequency for revising MSC definition

Closing remarks

Dr Bernard Thébaud

The Ottawa Hospital Research Institute | L'Hôpital d'Ottawa Institut de recherche

Inspired by research. **Driven** by compassion.
Inspiré par la recherche. **Guidé** par la compassion.

Affiliated with Affilié à uOttawa
