# Supplementary material for: Delphi-driven consensus definition for mesenchymal stromal cells and clinical reporting guidelines for mesenchymal stromal cell-based therapeutics
Source: Cytotherapy. Author manuscript; Available in PMC 2026 Feb 24. (PMC12931451; doi:10.1016/j.jcyt.2024.10.008)
Supplement: supp material 3 [file NIHMS2053365-supplement-supp_material_3.pdf]

Appendix 3. Revised Delphi questionnaire for 2nd online round.

Delphi questionnaire Mesenchymal Stromal Cells.

Dear colleague,

Thank you for participating in our international Delphi. This study aims to:

1) develop a **consensus definition** for Mesenchymal Stromal Cells (MSCs), and

2) to establish **reporting guidelines** for clinical studies using MSCs

The Delphi method is a structured communication method, which relies on a panel of experts contributing their views through a series of iterative surveys to reach a consensus.

As a participant in this round of the Delphi, you are asked to answer each item presented from your own perspective (i.e., rating relative importance of each item and providing rationale for your choices as appropriate). Your responses to each item will be aggregated with other participants and summarized for the next round of the Delphi. All responses will be presented anonymously.

For this second round of the Delphi, you will see questions that you voted on in the first round which did not reach consensus among participants. You will also be provided with the mean scores from round 1 participants for each item as well as any comments to explain their responses. Please review the question, the mean score, and the comments available before re-voting on the item. You will also be presented with new items that were suggested by participants for inclusion; you will have the chance to vote on whether you agree that these items should be included or not.

1. **Demographics.**

1.1. In what country do you currently reside?

- drop-down menu with country list

1.2. How would you describe your gender?

- Woman.
- Man.
- Other, please specify:
- Prefer not to say

1.3. What is your age category?

- 18 to 24 years old
- 25 to 34 years old
- 35 to 44 years old
- 45 to 54 years old
- 55 to 64 years old
- 65 years old or older

1.4. Are you currently conducting research? Yes/No

If Yes to Q1.4:

1.4.1. What is your research area? Please select all that apply.

- Blood and immune system
- Cancer
- Cardiovascular
- Digestive system
- Endocrine system
- Eye, ear, throat
- Genitourinary system
- Musculoskeletal system and connective tissue
- Nervous system
- Periodontology
- Respiratory system
- Skin and subcutaneous tissue

- Other, please specify:

1.4.2. What type of research do you do? Please select all that apply.

- Basic research
- Preclinical research (assessment of therapeutic effects in animal models)
- Clinical research
- Methodologist
- None of the above, please specify:

Based on the participant's selection to this question, the participant will answer to part 2 only, part 3 only or both parts 2 and 3 of the questionnaire

1.4.3. What is your career stage?

Describe the number of years since your first independent academic or research appointment (When we say 'independent' we refer to roles where you are no longer in a trainee position and are able to seek and hold your own funding for research):

- I am a trainee: BSc, MSc, PhD, Post-doc
- Less than 5 years.
- 5 to 15 years.
- More than 15 years.
- *Based on the participant's selection to this question, the participant will answer to part 2 only, part 3 only or both parts 2 and 3 of the questionnaire.*

1.5 Do you have any current involvement with a private stem cell company?

- Yes - I am employed at a private stem cell company
- Yes - I receive funding from a private stem cell company
- Yes - other, please specify
- No.

1.6. In which sector are you currently working? Select all that apply.

- University.
- Hospital.
- Regulatory Agency.
- Private company.
- Publishing sector.
- Non-profits organization.

- None of the above, please specify:

2. Items for Mesenchymal Stromal Cells definition and characterization.

Ten items from round 1 of this Delphi reached consensus for inclusion or exclusion in the definition and characterization section. Below you will be asked to re-vote on 17 items from round 1 as well as 5 newly suggested items from participants.

2.1 Mesenchymal Stromal Cell (MSC) is an appropriate term to maintain.

One concern expressed is whether the term MSC is appropriate to maintain considering that what we are defining as 'MSC' is often heterogeneous and describes unrelated cell types. Please indicate below whether you think the term MSC is appropriate to maintain. Consider that if the term is changed, a new more representative term would need to be identified.

'MSC' is an appropriate term to maintain, rate from 1 (strongly disagree) to 9 (strongly agree).

First round results.

Item mean score (/9): 7.3

<u>Selected comments.</u>

Mesenchymal stromal cell **is** an appropriate term to maintain for the following reasons:

- It includes an heterogenous population of cells isolated from a range of different tissues. It can be used as an umbrella term with further clear description of species, tissue origin and any other special attribute.
- Its major therapeutic effect in vivo is linked to secretory actions rather than stemness properties.
- The body of research built, most specifically on the acronym MSC is considerable. To any lay audience or newcomer, that continuity and name recognition is key to understanding the bigger differences in all the different flavors of cell therapies.

Mesenchymal stromal cell **is NOT** an appropriate term to maintain for the following reasons:

- These cells do not have a common embryonic origin and are not a lineage. There is an unrecognized battle between developmental biologists who may prefer the term endogenous mesodermal progenitors and the cell therapists who would want to maintain the term MSC for pragmatic reasons; in that case, it should be acknowledged that these are exogenous, culture adapted cells specifically for therapeutic purposes.
- This term is scientifically confusing and induces misrepresentation in both academia and commerce and exposes the public to exaggerated claims.

Add the option “Would like to discuss at the in-person meeting” with checkbox under the scale.

Please provide a rationale for why you responded above as you did: Free text answer.

2.2 During the first round, the following alternative denominations were proposed by participants.

For each denomination, rate from 1 (strongly disagree) to 9 (strongly agree).

- Vascular maintenance cell
- Multipotent stromal cell
- Fibroblastic stromal cell
- Tissue derived stromal cell
- Cultured stromal cell (followed by its tissue of origin)
- Mesenchymal signaling cell
- Mesenchymal stromal derived cell

Add the option “Would like to discuss at the in-person meeting” with checkbox under the scale.

Please provide a rationale for why you responded above as you did: Free text answer.

2.3. Are Mesenchymal Stromal Cell and Mesenchymal Stem Cells interchangeable terms?

Previous definitions use the term Mesenchymal Stromal Cell but in literature many authors use both **Mesenchymal Stromal Cell** and **Mesenchymal Stem Cell** to describe these cells.

Mesenchymal Stromal Cell and Mesenchymal Stem Cells are interchangeable terms. Rate from 1 (strongly disagree) to 9 (strongly agree).

First round results.

Item mean score (/9): 4.3

Selected comments:

Mesenchymal stromal cells and mesenchymal stem cells ***are*** *interchangeable terms:*

- *The body of research built, most specifically on the acronym MSC is considerable. To any lay audience or newcomer, that continuity and name recognition is key to understanding the bigger differences in all the different flavors of cell therapies.*
- *Some mesenchymal stromal cells have stemness properties, therefore it is acceptable to use both designations.*

*Mesenchymal stromal cells and mesenchymal stem cells **are NOT** interchangeable terms:*

- *"stem" should only refer to cells with proved stemness properties (I.e., self-renewal and multipotentiality).*
- *Vast majority of MSCs are just fibroblastic without stem cell properties.*
- *The vast majority of current MSC work is not using MSCs for their stem cell properties, but for other properties such as immune modulation.*
- *Using those terms interchangeably is scientifically confusing and induces misrepresentation in both academia and commerce and exposes the public to exaggerated claims.*

Add the option "Would like to discuss at the in-person meeting" with checkbox under the scale.

Please provide a rationale for why you responded above as you did: Free text answer.

2.4. For each item, indicate if you think the <u>item should be used to define and characterize MSC</u>.

2.4.1. Plastic adherence.

2.4.1. A description of MSC capacity to adhere to a plastic surface when maintained in standard culture condition, is essential to define them. Rate from 1 (strongly disagree) to 9 (strongly agree).

First round results.

Item mean score (/9): 6.5

<u>Selected comments</u>

PROS

- *Adherence to plastic is a standard method by which the cells can be isolated, and it is a feature of the cells in contrast to other nonadherent cells found in bone marrow and blood.*
- *This is the basic defining principle of MSC. Changing this could potentially create confusion regarding the applicability of new research to the historical body of evidence.*
- *Plastic adherence and typical fibroblast-like MSC appearance are a very simple but effective criteria to describe MSCs in addition to other criteria.*

CONS

- *Plastic adherence is not unique to MSC.*
- *MSC can be cultured in 3D spheroids or in suspension.*
- *Both adherent and non-adherent MSCs have been shown to have therapeutic benefits. If we want to encompass both types in the term MSC then adherence to plastic is not essential.*

Neutral

- *This is how they were defined originally. Might be inadequate as a marker but probably still part of the overall definition?*
- *Probably quite helpful as an initial criterion but should not be a single defining requirement.*

Add the option “Would like to discuss at the in-person meeting” with checkbox under the scale.

Please provide a rationale for why you responded above as you did: Free text answer.

2.4.2. Cell markers expression.

*2.4.2.a. The item “A description of MSC positive and negative markers is essential to define them” reached the **consensus for inclusion** (81%) during the first round.*

2.4.2.b. For MSC markers expression, the flow cytometry cut-off (% of cells) to consider a cell marker as a positive or a negative marker should be detailed in the Method section. Rate from 1 (strongly disagree) to 9 (strongly agree).

First round results.

Item mean score (/9): 7.3

Selected comments

PROS

- *Useful to gauge the purity of isolated MSC.*
- *Standard requirement of any flow cytometry assay.*
- *Allows for inter study comparison (transparency).*

CONS

- *Setting a threshold would only be useful if the markers used were specific to a cell type or functional status, and none of the currently used markers are.*

Neutral

- *Flow cytometry needs to be well-controlled to be considered valid - but cut-off or % of cells are not the only parameters to consider, clear discrimination of different populations is more useful (i.e., the ability to separate genuine positive staining for a marker from background fluorescence).*

Add the option "Would like to discuss at the in-person meeting" with checkbox under the scale.

Please provide a rationale for why you responded above as you did: Free text answer.

2.4.2.c. For MSC markers expression, the flow cytometry results with the % of positive cells should be described for each positive and negative marker in the Results section. Rate from 1 (strongly disagree) to 9 (strongly agree).

First round results.

Item mean score (/9): 6.8

Selected comments

PROS

- *This kind of description is helpful - but the flow cytometry plots should also be shown for quality assurance / comparative purposes.*
- *This is standard practice and assists in making the assay more rigorous so that other investigators can reproduce the same phenomenology.*
- *Allows for inter study comparison (transparency).*

CONS

- *The description in the Methods section is sufficient.*

Neutral

- *The specific % results may not be essential - if it is confirmed that the results were above the stipulated cut-off for positive markers, or below the stipulated cut-off for negative markers.*

Add the option “Would like to discuss at the in-person meeting” with checkbox under the scale.

Please provide a rationale for why you responded above as you did: Free text answer.

2.4.2.d. Among the list below, are the following <u>positive</u> cell markers essential to define MSC? Rate from 1 (strongly disagree) to 9 (strongly agree) and provide a rationale for your choice. You also have the possibility to add cell marker by selecting “Other, please specify” answer.

First round results.

Item mean score (/9): CD29+ (5.1), CD44+ (5.9), CD166+ (4.5), CD299+ (3.3)

*The following positive cell markers have reached **consensus for inclusion** during the first round: CD73+, CD90+, CD105+.*

<u>Selected comments</u>

- *HLA-I and HLA-II are of key functional importance in allorecognition and immunomodulation.*
- *CD29+, CD44+, CD73+, CD90+, and CD105+ are part of the conventional ISCT panel or already very commonly used for years.*
- *None of these markers are specific.*
- *The combination of markers is most important.*
- *The markers depend on the tissue origin of MSC.*
- *All surface markers are not directly linked to the function of MSC and vary in detection level between labs and culture conditions.*

Add the option “Would like to discuss at the in-person meeting” with checkbox under the scale.

- CD29+
- CD44+
- CD166+
- CD299+

The following positive cell markers have been proposed during the first round. Rate from 1 (strongly disagree) to 9 (strongly agree) and provide a rationale for your choice.

- CD10+
- CD140b+
- CD142+

- CD146+
- CD271+
- CD276+
- HLA-I
- SSEA-3
- SSEA-4
- Nestin

Add the option “Would like to discuss at the in-person meeting” with checkbox under the scale.

Please provide a rationale for why you responded above as you did: Free text answer.

2.4.2.e. Among the list below, are the following <u>negative</u> cell markers essential to define MSC?

Rate from 1 (strongly disagree) to 9 (strongly agree) and provide a rationale for your choice. You also have the possibility to add cell marker by selecting “Other, please specify” answer.

First round results.

Item mean score (/9): CD3- (4.9), CD11- (5.9), CD14- (6.6), CD19- (5.5), CD31- (6.3), CD34- (6.4), HLA DR- (6.2).

*The following negative cell marker has reached the **consensus for inclusion** during the first round: CD45-.*

<u>Selected comments</u>

- *Common MSC contaminants of hematopoietic origin that may also adhere to the culture can be identified with CD34, CD45, CD14, and HLA-DR.*

Add the option “Would like to discuss at the in-person meeting” with checkbox under the scale.

- CD3-
- CD11-
- CD14-
- CD19-
- CD31-
- CD34-
- HLA DR-

The following negative cell markers have been proposed during the first round. Rate from 1 (strongly disagree) to 9 (strongly agree) and provide a rationale for your choice.

- CD11-
- CD133-

Add the option "Would like to discuss at the in-person meeting" with checkbox under the scale.

Please provide a rationale for why you responded above as you did: Free text answer.

2.4.3. Differentiation.

2.4.3.a. A description of MSC in-vitro differentiation capacity (e.g., differentiation in adipocytes, chondrocytes...etc.) is essential to define them.

Rate from 1 (strongly disagree) to 9 (strongly agree).

First round results.

Item mean score (/9): 5.6

Selected comments

PROS
- This provides a measure of overall cell fitness and stem/progenitor activity.
- Tri-lineage differentiation into chondrocytes, adipose and bone are all hallmarks of MSCs.

CONS
- More important from a therapeutic perspective rather than for its definition. Plasticity is not the main feature of MSC, but paracrine-mediated effects on other cell targets.
- demonstration of mesodermal plasticity does not inform on any cellular attribute or functionality essential to establish identity.

Neutral
- Adipogenesis is a useful, robust, and simple functional assay; chondrogenic and osteogenic differentiation assays are still technically immature.

Add the option "Would like to discuss at the in-person meeting" with checkbox under the scale.

Please provide a rationale for why you responded above as you did: Free text answer.

2.4.3.b. Select which following differentiation assays are important to define MSC.

- Tri-lineage differentiation (i.e., adipocyte, osteoblast and chondrocyte)
- Adipocyte.

- Osteoblast.
- Chondroblast.
- None of these assays are important to define MSC

Add the option "Would like to discuss at the in-person meeting" with checkbox under the scale.

Please provide a rationale for why you responded above as you did: Free text answer.

2.4.3.c. The MSC in-vitro differentiation capacity should be qualitative.

Rate from 1 (strongly disagree) to 9 (strongly agree).

First round results.

Item mean score (/9): 5.5

Selected comments

- For MSC definition, qualitative assessment is sufficient (no need for quantitative assessment, especially as it's not reliable).
- MSCs from different sources and/or produced by different processes do not necessarily differentiate to the same extent into each lineage, but they should differentiate into each lineage to some extent. Consequently, a qualitative rather than quantitate assay is appropriate.

Add the option "Would like to discuss at the in-person meeting" with checkbox under the scale.

Please provide a rationale for why you responded above as you did: Free text answer.

2.4.3.d. The MSC in-vitro differentiation capacity should be quantitative.

Rate from 1 (strongly disagree) to 9 (strongly agree).

First round results.

Item mean score (/9): 5.1

Selected comments

- Significant variability in terms of differentiation efficiency exists between MSC isolates, therefore quantitative assays are recommended.
- Too difficult / time consuming to quantify.

Add the option "Would like to discuss at the in-person meeting" with checkbox under the scale.

Please provide a rationale for why you responded above as you did: Free text answer.

2.4.4. Tissue origin.

*2.4.4.a. The item "A description of where the MSC cells were sourced from is essential to characterize them" reached the **consensus for inclusion** (86%) during the first round.*

2.4.4.b. Among the list below, indicate if the following tissues are a source of MSC.

Rate from 1 (strongly disagree) to 9 (strongly agree) and provide a rationale for your choice. You also have the possibility to add cell marker by selecting "Other, please specify" answer.

First round results.

Item mean score (/9): Umbilical cord blood (6.3), Synovial (6.4), peripheral blood (3.9)

*The following tissues have reached the **consensus for inclusion** during the first round: bone marrow, adipose tissue, umbilical cord (Wharton jelly), placenta/amnion.*

Add the option "Would like to discuss at the in-person meeting" with checkbox under the scale.

- Umbilical cord blood.
- Synovial.
- Peripheral blood.
- Other, please specify:

The following tissue sources have been proposed during the first round. Rate from 1 (strongly disagree) to 9 (strongly agree) and provide a rationale for your choice.

- Dental follicle
- Menstrual blood
- iPSC and fetal tissue
- Most tissues (excluding central nervous system)
- Most tissues (including central nervous system)
- Amniotic fluid

Add the option "Would like to discuss at the in-person meeting" with checkbox under the scale.

Please provide a rationale for why you responded above as you did: Free text answer.

2.4.5. Evidence of stemness in-vitro.

2.4.5.a. A description of self-renewal and multilineage differentiation capacities is essential to define MSC.

Rate from 1 (strongly disagree) to 9 (strongly agree).

First round results.

Item mean score (/9): 5.6

Selected comments

NB. Comments for this item differed depending on if the participant considered the 'S' in MSC as Stem or Stromal

- *Unnecessary if we consider that MSC are stromal cells (I.e., no stemness properties).*
- *Can be useful to assess the cell function (If you want to claim they have stem/progenitor properties) but not to define them.*

Add the option "Would like to discuss at the in-person meeting" with checkbox under the scale.

Please provide a rationale for why you responded above as you did: Free text answer.

2.4.5.b. The description of the specific method used to assess MSC stemness in-vitro is essential to define MSC.

Rate from 1 (strongly disagree) to 9 (strongly agree).

First round results.

Item mean score (/9): 6.3

Selected comments

- *If claiming stem cell properties, this would be essential; if not, no methodology is needed.*

Add the option "Would like to discuss at the in-person meeting" with checkbox under the scale.

Please provide a rationale for why you responded above as you did: Free text answer.

2.4.6. In-vitro functional assays.

2.4.6. A description of in-vitro functional assays (using quantitative RNA analysis of selected genes, proteins analysis of MSC secretome...etc.) to assess MSCs' potency and properties (e.g., trophic factors secretion, immunomodulation...etc.) is essential to characterize MSC.

Rate from 1 (strongly disagree) to 9 (strongly agree).

First round results.

Item mean score (/9): 6.6

Selected comments

PROS

- *Critical if MSCs are used for clinical purposes.*
- *Since no true phenotypic identity is established, and phenotypic markers are insufficient to describe MSCs, a complementary set of functional assays is essential to better describe MSC potency and properties.*

CONS

- *Not necessary to define MSC but important to characterize them and their therapeutic potential.*
- *Access and affordability of these assays should be considered if we decided these are standard to define MSC.*
- *Using them to define a population as MSC presupposes that we know exactly what transcriptome, gene expression profile etc. should be seen in a definitive population.*

Add the option "Would like to discuss at the in-person meeting" with checkbox under the scale.

Please provide a rationale for why you responded above as you did: Free text answer.

2.5. MSC licensing.

2.5.a. MSC licensing, i.e. preconditioned in-vitro by pro-inflammatory cytokines exposure to mimic in vivo inflammatory environment, is essential to characterize MSC.

Rate from 1 (strongly disagree) to 9 (strongly agree).

First round results.

Item mean score (/9): 4.5

Selected comments

- *No use to define MSC but can be of interest to characterize MSC for applications where the proposed mechanism of action relates to inflammatory responses.*

Add the option "Would like to discuss at the in-person meeting" with checkbox under the scale.

Please provide a rationale for why you responded above as you did: Free text answer.

2.5.b. Molecules used for licensing should be described when defining MSC.

Rate from 1 (strongly disagree) to 9 (strongly agree).

First round results.

Item mean score (/9): 7.1

Selected comments

- *A full characterization of molecules that are used to elicit a specific function from MSC is an important aspect of understanding the mechanisms behind the effect.*
- *Independent of the real impact of licensing. Culture conditions need to be described in detail. So, if authors add licensing factors it needs to be reported.*

Add the option "Would like to discuss at the in-person meeting" with checkbox under the scale.

Please provide a rationale for why you responded above as you did: Free text answer.

2.5.c. Resting (non-licensed) MSC should be used as an internal control when defining MSC.
Rate from 1 (strongly disagree) to 9 (strongly agree).

First round results.

Item mean score (/9): 6.6

Add the option “Would like to discuss at the in-person meeting” with checkbox under the scale.

Please provide a rationale for why you responded above as you did: Free text answer.

2.6. Are there any characteristics of MSC that are not in the table above that you think should be considered essential to define or characterize MSC?

The following answers were proposed by participants during the first round. Rate from 1 (strongly disagree) to 9 (strongly agree).

- Transcriptome analysis (e.g., single-cell RNA sequencing)
- Secretome profile
- Exosomes (exosome signature, quantitative measurement)
- Immunomodulatory and MLR assays
- Angiogenic assays
- Transcription factors expression (e.g., gene expression analysis for OCT4, SOX2, etc.)
- DNA methylation profile

Add the option "Would like to discuss at the in-person meeting" with checkbox under the scale.

Please provide a rationale for why you responded above as you did: Free text answer.

2.7. What have we missed?

Please add any additional item you find relevant for MSC definition or characterization and provide a rationale to support your answer (free text box).

3. Items for reporting guidelines for clinical trials using MSC.

The aim of this Delphi questionnaire is to identify consensus items that should be reported in clinical studies using MSC; these items will be used to develop reporting guidelines for MSC clinical research. These reporting guidelines will provide international standards to be adopted and implemented by journals as well as regulatory and funding agencies to improve quality, transparency and reproducibility of MSC clinical research. Twenty-three items from round 1 of this Delphi reached consensus for inclusion in the reporting guideline. Below you will be asked to re-vote on 5 items from round 1 as well as 5 newly suggested items from participants.

3.1. MSC intervention group and control.

For each item below, describe if the item should be reported in MSC clinical studies and elaborate your rating.

3.1.1. MSC administration route.

3.1.1. MSC clinical studies should report the MSC administration route (e.g., Intra-venous, intra-articular, etc.). This item reached ***consensus for inclusion*** *(96%) during the first round.*

3.1.2. MSCs dose.

3.1.2.a. MSC clinical studies should report the MSC dose in the intervention group. This item reached ***consensus for inclusion*** *(96%) during the first round.*

3.1.2.b. The MSC dose should be reported as a dose normalized to weight (number of cells per kilogram of bodyweight). Rate from 1 (strongly disagree) to 9 (strongly agree).

First round results.

Item mean score (/9): 7.0

Selected comments

PROS

- *Similar to drugs, having the dosing reported in this manner would be extremely helpful in terms of standardization.*

CONS

- *This is only essential for intravenous infusion. Many other localized deliveries are used.*
- *Patients' bodyweight does not influence MSC distribution or performance much.*

Add the option "Would like to discuss at the in-person meeting" with checkbox under the scale.

Please provide a rationale for why you responded above as you did: Free text answer.

3.1.3. MSC product.

3.1.3.a. MSC clinical studies should report the MSC product concentration (i.e., concentration (number of cells per milliliter of vehicle) of the cell product administered to the patient).

*This item reached **consensus for inclusion** (87%) during the first round.*

3.1.3.b. MSC clinical studies should report the vehicle in which MSC are delivered to the patient.

*This item reached **consensus for inclusion** (93%) during the first round.*

3.1.4. MSC infusion rate.

3.1.4. MSC clinical studies using intra-venous route for MSC administration, should report the MSC solution infusion rate.

*This item reached **consensus for inclusion** (83%) during the first round.*

3.1.5. Use of adjuvants for MSC preparation.

3.1.5. MSC clinical studies should report if they used adjuvants during the preparation or processing of MSCs (e.g., use of dimethyl sulfoxide (DMSO) for MSC preparation should be reported).

*This item reached **consensus for inclusion** (96%) during the first round.*

3.1.6. Control group.

3.1.6.a. When the study design involves a control group, MSC clinical studies should report the characteristics of the control group.

*This item reached **consensus for inclusion** (99%) during the first round.*

3.1.6.b. MSC clinical studies should describe the type of control used.

*This item reached **consensus for inclusion** (96%) during the first round.*

3.1.7 What have we missed?

Please add any additional item you find relevant to report to describe MSC intervention group and control in clinical trials using MSC and provide a rationale to support your answer (free text box).

3.2. MSC characteristics.

For each item below, describe if the item should be reported in MSC clinical studies and elaborate your rating.

3.2.1. MSC provenance.

3.2.1.a. MSC clinical studies should report MSC provenance (e.g., MSC provenance can be from patient, donor or cell from stem cell company).

*This item reached **consensus for inclusion** (96%) during the first round.*

3.2.1.b. MSCs' Donor characteristics (e.g., age, sex, BMI, health status, medication, smoking) should be extensively described (new item).

Rate from 1 (strongly disagree) to 9 (strongly agree).

Add the option "Would like to discuss at the in-person meeting" with checkbox under the scale.

Please provide a rationale for why you responded above as you did: Free text answer.

3.2.1.c. MSC clinical studies should report the tissue source of the MSC (e.g., Bone marrow, adipose tissue...etc.).

*This item reached **consensus for inclusion** (94%) during the first round.*

3.2.1.d. MSC clinical studies should report and describe the extraction procedure used to obtain MSC from the tissue source (e.g., enzymatic digestion, mechanical...etc.).

*This item reached **consensus for inclusion** (87%) during the first round.*

3.2.2. MSC immune compatibility

3.2.2. MSC clinical studies should report the immune compatibility between MSC and patient (e.g., Autologous, unmatched allogenic, matched allogenic).

*This item reached **consensus for inclusion** (87%) during the first round.*

3.2.3. MSC "fitness”

3.2.3.a. MSC clinical studies should report the MSC state prior to administration (e.g., Fresh vs. cryopreserved).

*This item reached **consensus for inclusion** (91%) during the first round.*

3.2.3.b For studies using cryopreserved MSC, the number of months the cells were frozen prior to patient administration should be described.

Rate from 1 (strongly disagree) to 9 (strongly agree).

First round results.

Item mean score (/9): 6.1

Selected comments

- *If cells are frozen properly and maintained appropriately, the length of time they are frozen is not important.*
- *Fitness of the MSCs (fresh or thawed) at the time of administration is the most important metric, irrespective of how long the cells were cryopreserved.*

Add the option “Would like to discuss at the in-person meeting” with checkbox under the scale.

Please provide a rationale for why you responded above as you did: Free text answer.

3.23.c. When a clinical study used cryopreserved MSC, MSC conditioning prior to administration (e.g., frozen/thawed/administration or frozen/thawed/cultured/administration) should be described.

*This item reached **consensus for inclusion** (90%) during the first round.*

3.2.3.d. MSC clinical studies should report the population doubling time (PDT) of the MSC used in the intervention group.

Rate from 1 (strongly disagree) to 9 (strongly agree).

First round results.

Item mean score (/9): 6.8

Selected comments

- *Too much variability to make this a useful metric.*
- *Very important info: some recent studies found that a good/preferable PDT or good growth was correlated with better clinical outcome.*

Add the option "Would like to discuss at the in-person meeting" with checkbox under the scale.

Please provide a rationale for why you responded above as you did: Free text answer.

3.2.3.e. Any functional assay performed on the cell product and its results should be reported (new item).

Rate from 1 (strongly disagree) to 9 (strongly agree).

Add the option "Would like to discuss at the in-person meeting" with checkbox under the scale.

Please provide a rationale for why you responded above as you did: Free text answer.

3.2.3.f. Describing if the MSC used in the clinical trial is derived from the same batch or different batches is important to report.

Rate from 1 (strongly disagree) to 9 (strongly agree).

Add the option "Would like to discuss at the in-person meeting" with checkbox under the scale.

Please provide a rationale for why you responded above as you did: Free text answer.

3.2.4. MSC viability

3.2.4.a. MSC clinical studies should report MSC viability assessment prior to administration.

*This item reached **consensus for inclusion** (91%) during the first round.*

3.2.4.b. MSC clinical studies should report the type of viability assay used.

*This item reached **consensus for inclusion** (85%) during the first round.*

3.2.4.c. MSC clinical studies should report the viability assay results.

*This item reached **consensus for inclusion** (85%) during the first round.*

3.2.5 What have we missed?

Please add any additional item you find relevant to report to describe MSC characteristics in clinical trials using MSC and provide a rationale to support your answer (free text box).

3.3. MSC culture condition.

For each item below, describe if the item should be reported in MSC clinical trial and elaborate your rating.

3.3.1. Method of culture

3.3.1 MSC clinical studies should report the method used to culture the MSC (2D vs. 3D culture) (new item).
Rate from 1 (strongly disagree) to 9 (strongly agree).

Add the option "Would like to discuss at the in-person meeting" with checkbox under the scale.

Please provide a rationale for why you responded above as you did: Free text answer.

3.3.2. Oxygen environment

3.3.2 MSC clinical studies should report the level of oxygen used for MSC culture (e.g., 5% vs. 21% of Oxygen).

*This item reached **consensus for inclusion** (83%) during the first round.*

3.3.3. Cell confluence.

3.3.3 MSC clinical studies using fresh MSC or cryopreserved MSC with culture prior to administration should report the level of cells confluence (in %) used to harvest the cells for administration to the patient.

Rate from 1 (strongly disagree) to 9 (strongly agree).

First round results.

Item mean score (/9): 6.8

Selected comments

- Confluence assessment is highly subjective.
- Important cell culture parameter with high impact on MSC proliferation and functions.

Add the option "Would like to discuss at the in-person meeting" with checkbox under the scale.

Please provide a rationale for why you responded above as you did: Free text answer.

3.3.4. Culture medium.

3.3.4.a. MSC clinical studies should report the culture medium used for MSC culture (e.g., DMEM, alpha MEM...etc.).

Rate from 1 (strongly disagree) to 9 (strongly agree).

First round results.

Item mean score (/9): 7.7

Selected comments

- Base media is less important than supplements added to it.
- Essential for standardization and reproducibility.

Add the option “Would like to discuss at the in-person meeting” with checkbox under the scale.

Please provide a rationale for why you responded above as you did: Free text answer.

3.3.4.b. MSC clinical studies should report media and reagents catalog numbers in the method section.

Rate from 1 (strongly disagree) to 9 (strongly agree).

Add the option “Would like to discuss at the in-person meeting” with checkbox under the scale.

Please provide a rationale for why you responded above as you did: Free text answer.

3.3.5. Use of serum

3.3.5.a. MSC clinical studies should report the use or not of serum for culture.

*This item reached **consensus for inclusion** (81%) during the first round.*

3.3.5.b. MSC clinical studies should report the type of serum used.

*This item reached **consensus for inclusion** (84%) during the first round.*

3.3.5.c. MSC clinical studies should report the amount (in % of the total amount of culture medium) of serum used.

*This item reached **consensus for inclusion** (87%) during the first round.*

3.3.6. Use of Human platelet lysate

3.3.6.a. MSC clinical studies should report the use or not of Human platelet lysate for culture.

*This item reached **consensus for inclusion** (86%) during the first round.*

3.3.6.b. MSC clinical studies should report the amount (in % of the total amount of culture medium) of Human platelet lysate used.

*This item reached **consensus for inclusion** (81%) during the first round.*

3.3.7. What have we missed?

Please add any additional item you find relevant to report to describe MSC culture condition in clinical trials using MSC and provide a rationale to support your answer (free text box).
