# Supplementary material for: Delphi-driven consensus definition for mesenchymal stromal cells and clinical reporting guidelines for mesenchymal stromal cell-based therapeutics
Source: Cytotherapy. Author manuscript; Available in PMC 2026 Feb 24. (PMC12931451; doi:10.1016/j.jcyt.2024.10.008)
Supplement: supp material 2 [file NIHMS2053365-supplement-supp_material_2.pdf]

Appendix 2. Initial Delphi Questionnaire (First online round)

Delphi questionnaire Mesenchymal Stromal Cells.

Dear colleague,

Thank you for participating in our international Delphi. This study aims to:

1) develop a **consensus definition** for Mesenchymal Stromal Cells (MSCs), and

2) to establish **reporting guidelines** for clinical studies using MSCs

The Delphi method is a structured communication method, which relies on a panel of experts contributing their views through a series of iterative surveys to reach a consensus.

As a participant in this round of the Delphi, you are asked to answer each item presented from your own perspective (i.e., rating relative importance of each item and providing rationale for your choices as appropriate). Your responses to each item will be aggregated with other participants and summarized for the next round of the Delphi. All responses will be presented anonymously.

1. **Demographics.**

1.1. In what country do you currently reside?

- drop-down menu with country list

1.2. How would you describe your gender?

- Woman.
- Man.
- Other, please specify:
- Prefer not to say

1.3. What is your age category?

- 18 to 24 years old
- 25 to 34 years old
- 35 to 44 years old
- 45 to 54 years old
- 55 to 64 years old
- 65 years old or older

1.4. Are you currently conducting research? Yes/No

If Yes to Q1.4:

1.4.1. What is your research area? Please select all that apply.

- Blood and immune system
- Cancer
- Cardiovascular
- Digestive system
- Endocrine system
- Eye, ear, throat
- Genitourinary system
- Musculoskeletal system and connective tissue
- Nervous system
- Periodontology
- Respiratory system
- Skin and subcutaneous tissue
- Other, please specify:

1.4.2. What type of research do you do? Please select all that apply.

- Basic research
- Preclinical research (assessment of therapeutic effects in animal models)
- Clinical research
- Methodologist
- None of the above, please specify:

Based on the participant's selection to this question, the participant will answer to part 2 only, part 3 only or both parts 2 and 3 of the questionnaire

1.4.3. What is your career stage?

Describe the number of years since your first independent academic or research appointment (When we say 'independent' we refer to roles where you are no longer in a trainee position and are able to seek and hold your own funding for research):

- I am a trainee: BSc, MSc, PhD, Post-doc
- Less than 5 years.
- 5 to 15 years.
- More than 15 years.
- *Based on the participant's selection to this question, the participant will answer to part 2 only, part 3 only or both parts 2 and 3 of the questionnaire.*

1.5 Do you have any current involvement with a private stem cell company?

- Yes - I am employed at a private stem cell company
- Yes - I receive funding from a private stem cell company
- Yes - other, please specify
- No.

1.6. In which sector are you currently working? Select all that apply.

- University.
- Hospital.
- Regulatory Agency.
- Private company.
- Publishing sector.
- Non-profits organization.
- None of the above, please specify:

2. Items for Mesenchymal Stromal Cells definition and characterization.

2.1 Mesenchymal Stromal Cell.

One concern expressed is whether the term MSC is appropriate to maintain considering that what we are defining as 'MSC' is often heterogeneous and describes unrelated cell types. Please indicate below whether you think the term MSC is appropriate to maintain. Consider that if the term is changed, a new more representative term would need to be identified.

'MSC' is an appropriate term to maintain, rate from 1 (strongly disagree) to 9 (strongly agree).

Please provide a rationale for why you responded above as you did: Free text answer.

If you disagree, what term would you proposed as an alternative? Free text

2.2. Are Mesenchymal S<u>tromal</u> Cell and Mesenchymal S<u>tem</u> Cells interchangeable terms?

Previous definitions use the term of Mesenchymal Stromal Cell but in the literature many authors use both **Mesenchymal Stromal Cell** and **Mesenchymal Stem Cell** to describe these cells.

Mesenchymal Stromal Cell and Mesenchymal Stem Cells are interchangeable terms. Rate from 1 (strongly disagree) to 9 (strongly agree).

Please provide a rationale for why you responded above as you did: Free text answer.

2.3. For each item, indicate if you think the item should be used to define and characterize MSC.

2.3.1. Plastic adherence.

A description of MSC capacity to adhere to a plastic surface when maintained in standard culture condition, is essential to define them. Rate from 1 (strongly disagree) to 9 (strongly agree).

Please provide a rationale for why you responded above as you did: Free text answer.

2.3.2. Cell markers expression.

A description of MSC positive and negative markers is essential to define them. Rate from 1 (strongly disagree) to 9 (strongly agree).

Please provide a rationale for why you responded above as you did: Free text answer.

For MSC markers expression, the flow cytometry cut-off (% of cells) to consider a cell marker as a positive or a negative marker should be detailed in the Method section. Rate from 1 (strongly disagree) to 9 (strongly agree).

Please provide a rationale for why you responded above as you did: Free text answer.

For MSC markers expression, the flow cytometry results with the % of positive cells should be described for each positive and negative marker in the Results section. Rate from 1 (strongly disagree) to 9 (strongly agree).

Please provide a rationale for why you responded above as you did: Free text answer.

Among the list below, are the following <u>positive</u> cell markers essential to define MSC? Rate from 1 (strongly disagree) to 9 (strongly agree), and provide a rationale for your choice. You also have the possibility to add cell marker by selecting "Other, please specify" answer.

- CD29+
- CD44+
- CD73+
- CD90+
- CD105+
- CD166+
- CD299+
- Other, please specify:

Among the list below, are the following <u>negative</u> cell markers essential to define MSC? Rate from 1 (strongly disagree) to 9 (strongly agree), and provide a rationale for your choice. You also have the possibility to add cell marker by selecting "Other, please specify" answer.

- CD3-
- CD11-
- CD14-
- CD19-
- CD31-
- CD34-
- CD45-
- HLA DR-
- Other, please specify:

2.3.3. Differentiation.

A description of MSC in-vitro differentiation capacity (e.g., differentiation in adipocytes, chondrocytes...etc.) is essential to define them. Rate from 1 (strongly disagree) to 9 (strongly agree).

Please provide a rationale for why you responded above as you did: Free text answer.

Select which following differentiation assays are important to define MSC.

- Tri-lineage differentiation (i.e., adipocyte, osteoblast and chondrocyte)
- Adipocyte.
- Osteoblast.
- Chondroblast.
- None of these assays are important to define MSC

The MSC in-vitro differentiation capacity should be qualitative. Rate from 1 (strongly disagree) to 9 (strongly agree).

Please provide a rationale for why you responded above as you did: Free text answer.

The MSC in-vitro differentiation capacity should be quantitative. Rate from 1 (strongly disagree) to 9 (strongly agree).

Please provide a rationale for why you responded above as you did: Free text answer.

2.3.4. Tissue origin.

A description of where the MSC cells were sourced from is essential to characterize them. Rate from 1 (strongly disagree) to 9 (strongly agree).

Please provide a rationale for why you responded above as you did: Free text answer.

Among the list below, indicate if the following tissues are a source of MSC. Rate from 1 (strongly disagree) to 9 (strongly agree), and provide a rationale for your choice. You also have the possibility to add cell marker by selecting "Other, please specify" answer.

- Bone marrow.
- Adipose tissue.
- Umbilical cord (Wharton jelly).
- Umbilical cord blood.
- Synovial.
- Placenta/amnion.
- Peripheral blood.
- Other, please specify:

2.3.5. Evidence of stemness in-vitro.

A description of self-renewal and multilineage differentiation capacities is essential defining MSC. Rate from 1 (strongly disagree) to 9 (strongly agree).

Please provide a rationale for why you responded above as you did: Free text answer.

The description of the specific method used to assess MSC stemness in-vitro is essential. Rate from 1 (strongly disagree) to 9 (strongly agree).

Please provide a rationale for why you responded above as you did: Free text answer.

2.3.6. In-vitro functional assays.

A description of in-vitro functional assays (using quantitative RNA analysis of selected genes, proteins analysis of MSC secretome...etc.) to assess MSCs' potency and properties (e.g., trophic factors secretion, immunomodulation...etc.) is essential to characterize MSC. Rate from 1 (strongly disagree) to 9 (strongly agree).

Please provide a rationale for why you responded above as you did: Free text answer.

Relevant functional assays for MSC characterization depend on research context. Among the list below, please indicate which functional assay could be important to assess MSCs' potency. Rate from 1 (strongly disagree) to 9 (strongly agree), and provide a rationale for your choice. You also have the possibility to add functional assay by selecting "Other, please specify" answer.

- Mixed lymphocyte reaction
- Migration assay (scratch assay etc.)
- Other, please specify:

2.4. MSC licensing.

MSC licensing, i.e. preconditioned in-vitro by pro-inflammatory cytokines exposure in order to mimic in vivo inflammatory environment, is essential to characterize MSC. Rate from 1 (strongly disagree) to 9 (strongly agree).

Please provide a rationale for why you responded above as you did: Free text answer.

Molecules used for licensing should be described. Rate from 1 (strongly disagree) to 9 (strongly agree).

Please provide a rationale for why you responded above as you did: Free text answer.

Resting (non-licensed) MSC should be used as an internal control. Rate from 1 (strongly disagree) to 9 (strongly agree).

Please provide a rationale for why you responded above as you did: Free text answer.

2.5. Are there any characteristics of MSC that are not in the table above that you think should be considered essential to define or characterize MSC?

- Free-text box.

2.6. What have we missed?

Please add any additional item you find relevant for MSC definition or characterization and provide a rationale to support your answer (free text box).

3. Items for reporting guidelines for clinical trials using MSC.

The aim of this Delphi questionnaire is to identify consensus items that should be reported in clinical studies using MSC; these items will be used to develop reporting guidelines for MSC clinical research. These reporting guidelines will provide international standard to be adopted and implemented by journals as well as regulatory and funding agencies to improve quality, transparency and reproducibility of MSC clinical research.

3.1. MSC intervention group and control.

For each item below, describe if the item should be reported in MSC clinical studies and elaborate your rating.

3.1.1. MSC administration route.

MSC clinical studies should report the MSC administration route (e.g., Intra-venous, intra-articular, etc.). Rate from 1 (strongly disagree) to 9 (strongly agree).

Please provide a rationale for why you responded above as you did: Free text answer.

3.1.2. MSCs dose.

MSC clinical studies should report the MSC dose in the intervention group. Rate from 1 (strongly disagree) to 9 (strongly agree).

Please provide a rationale for why you responded above as you did: Free text answer.

The MSC dose should be reported as a dose normalized to weight (number of cells per kilogram of bodyweight). Rate from 1 (strongly disagree) to 9 (strongly agree).

Please provide a rationale for why you responded above as you did: Free text answer.

3.1.3. MSC product.

MSC clinical studies should report the MSC product concentration (i.e., concentration (number of cells per milliliter of vehicle) of the cell product administered to the patient). Rate from 1 (strongly disagree) to 9 (strongly agree).

Please provide a rationale for why you responded above as you did: Free text answer.

MSC clinical studies should report the vehicle in which MSC are delivered to the patient. Rate from 1 (strongly disagree) to 9 (strongly agree).

Please provide a rationale for why you responded above as you did: Free text answer.

3.1.4. MSC infusion rate.

MSC clinical studies using intra-venous route for MSC administration, should report the MSC solution infusion rate. Rate from 1 (strongly disagree) to 9 (strongly agree).

Please provide a rationale for why you responded above as you did: Free text answer.

3.1.5. Use of adjuvants for MSC preparation.

MSC clinical studies should report if they used adjuvants during the preparation or processing of MSCs (e.g., use of dimethyl sulfoxide (DMSO) for MSC preparation should be reported). Rate from 1 (strongly disagree) to 9 (strongly agree).

Please provide a rationale for why you responded above as you did: Free text answer.

3.1.6. Control group.

When the study design involves a control group, MSC clinical studies should report this control group. Rate from 1 (strongly disagree) to 9 (strongly agree).

Please provide a rationale for why you responded above as you did: Free text answer.

MSC clinical studies should describe the type of control used. Rate from 1 (strongly disagree) to 9 (strongly agree).

Please provide a rationale for why you responded above as you did: Free text answer.

3.1.7 What have we missed?

Please add any additional item you find relevant to report to describe MSC intervention group and control in clinical trials using MSC, and provide a rationale to support your answer (free text box).

3.2. MSC characteristics.

For each item below, describe if the item should be reported in MSC clinical studies and elaborate your rating.

3.2.1. MSC provenance.

MSC clinical studies should report MSC provenance (e.g., MSC provenance can be from patient, donor or cell from stem cell company). Rate from 1 (strongly disagree) to 9 (strongly agree).

Please provide a rationale for why you responded above as you did: Free text answer.

MSC clinical studies should report the tissue source of the MSC (e.g., Bone marrow, adipose tissue...etc.). Rate from 1 (strongly disagree) to 9 (strongly agree).

Please provide a rationale for why you responded above as you did: Free text answer.

MSC clinical studies should report and describe the extraction procedure used to obtain MSC from the tissue source (e.g., enzymatic digestion, mechanical...etc.). Rate from 1 (strongly disagree) to 9 (strongly agree).

Please provide a rationale for why you responded above as you did: Free text answer.

3.2.2. MSC immune compatibility

MSC clinical studies should report the immune compatibility between MSC and patient (e.g., Autologous, unmatched allogenic, matched allogenic). Rate from 1 (strongly disagree) to 9 (strongly agree).

Please provide a rationale for why you responded above as you did: Free text answer.

3.2.3. MSC "fitness”

MSC clinical studies should report the MSC state prior to administration (e.g., Fresh vs. cryopreserved). Rate from 1 (strongly disagree) to 9 (strongly agree).

Please provide a rationale for why you responded above as you did: Free text answer.

For studies using cryopreserved MSC, the number of months the cells were frozen prior to patient administration should be described. Rate from 1 (strongly disagree) to 9 (strongly agree).

Please provide a rationale for why you responded above as you did: Free text answer.

When a clinical study used cryopreserved MSC, MSC conditioning prior to administration (e.g., frozen/thawed/administration or frozen/thawed/cultured/administration) should be described. Rate from 1 (strongly disagree) to 9 (strongly agree).

Please provide a rationale for why you responded above as you did: Free text answer.

MSC clinical studies should report the population doubling time (PDT) of the MSC used in the intervention group. Rate from 1 (strongly disagree) to 9 (strongly agree).

Please provide a rationale for why you responded above as you did: Free text answer.

3.2.4. MSC viability

MSC clinical studies should report MSC viability assessment prior to administration. Rate from 1 (strongly disagree) to 9 (strongly agree).

Please provide a rationale for why you responded above as you did: Free text answer.

MSC clinical studies should report the type of viability assay used. Rate from 1 (strongly disagree) to 9 (strongly agree).

Please provide a rationale for why you responded above as you did: Free text answer.

MSC clinical studies should report the viability assay results. Rate from 1 (strongly disagree) to 9 (strongly agree).

Please provide a rationale for why you responded above as you did: Free text answer.

3.2.5 What have we missed?

Please add any additional item you find relevant to report to describe MSC characteristics in clinical trials using MSC, and provide a rationale to support your answer (free text box).

3.3. MSC culture condition.

For each item below, describe if the item should be reported in MSC clinical trial and elaborate your rating.

3.3.1. Oxygen environment

MSC clinical studies should report the level of oxygen used for MSC culture (eg. 5% vs. 21% of Oxygen). Rate from 1 (strongly disagree) to 9 (strongly agree).

Please provide a rationale for why you responded above as you did: Free text answer.

3.3.2. Cell confluence.

MSC clinical studies using fresh MSC or cryopreserved MSC with culture prior to administration should report the level of cells confluence (in %) used to harvest the cells for administration to the patient. Rate from 1 (strongly disagree) to 9 (strongly agree).

Please provide a rationale for why you responded above as you did: Free text answer.

3.3.3. Culture medium.

MSC clinical studies should report the culture medium used for MSC culture (e.g., DMEM, alpha MEM...etc.). Rate from 1 (strongly disagree) to 9 (strongly agree).

Please provide a rationale for why you responded above as you did: Free text answer.

3.3.4. Use of serum

MSC clinical studies should report the use or not of serum for culture. Rate from 1 (strongly disagree) to 9 (strongly agree).

Please provide a rationale for why you responded above as you did: Free text answer.

MSC clinical studies should report the type of serum used. Rate from 1 (strongly disagree) to 9 (strongly agree).

Please provide a rationale for why you responded above as you did: Free text answer.

MSC clinical studies should report the amount (in % of the total amount of culture medium) of serum used. Rate from 1 (strongly disagree) to 9 (strongly agree).

Please provide a rationale for why you responded above as you did: Free text answer.

3.3.5 Use of Human platelet lysate

MSC clinical studies should report the use or not of Human platelet lysate for culture. Rate from 1 (strongly disagree) to 9 (strongly agree).

Please provide a rationale for why you responded above as you did: Free text answer.

MSC clinical studies should report the amount (in % of the total amount of culture medium) of Human platelet lysate used. Rate from 1 (strongly disagree) to 9 (strongly agree).

Please provide a rationale for why you responded above as you did: Free text answer.

3.3.6. What have we missed?

Please add any additional item you find relevant to report to describe MSC culture condition in clinical trials using MSC, and provide a rationale to support your answer (free text box).
