# Supplementary material for: Delphi-driven consensus definition for mesenchymal stromal cells and clinical reporting guidelines for mesenchymal stromal cell-based therapeutics
Source: Cytotherapy. Author manuscript; Available in PMC 2026 Feb 24. (PMC12931451; doi:10.1016/j.jcyt.2024.10.008)
Supplement: supp material 4 [file NIHMS2053365-supplement-supp_material_4.pdf]

Meeting Handbook:

Establish a consensus definition and clinical reporting guidelines for clinical trials for mesenchymal stromal cell

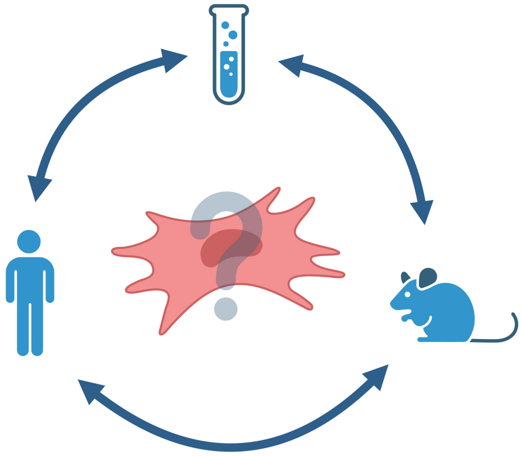

January 09 and 10, 2024

Table of Contents

Goals of the Consensus Meeting 03
Meeting Agenda 04
Summary of Rounds 1 & 2 06
Round 3 Voting explanation 15
Core Group Members 16
Organising Team's Biographies 18
Funding and acknowledgements 19

Goals of the Consensus Meeting

Welcome and thank you for taking the time to participate in this upcoming virtual event!

The key goals of this Delphi round will be to agree on a core set of items to:

1. **Develop a consensus definition for MSC**
2. **Establish reporting guidelines for clinical studies using MSC**

This meeting will represent the final round of a 3-round modified Delphi study to establish a consensus definition and clinical reporting guidelines for clinical trials for mesenchymal stromal cell. Rounds 1 and 2 took the form of an online survey.

We acknowledge this is a controversial topic, and the goal is to move forward as a community to reach a consensus (each participant will not agree 100% with all items discussed).

Our motto for the consensus meeting will be: Be kind and play together.

Everyone will have an opportunity to speak and listen, rather than the loudest person dominating the conversation.

During the 2 day meeting, the focus of our efforts will be to examine 22 items that have not yet achieved consensus (18 for MSC definition and 4 for reporting guidelines).

Thank you for your upcoming participation and collaboration to bring this project to fruition!

Meeting Agenda

January 09 (Day 1): Reporting Guidelines

Time (EST)	Activity	Speaker(s)
14:00 – 14:05	Welcome	Bernard Thébaud
14:05 – 14: 10	Summary of Rounds 1 & 2	Laurent Renesme
14:10 – 14:30	Round 3 voting	Manoj Lalu/ Kelly Cobey
14:30 – 14:45	Implementation	Manoj Lalu/ Kelly Cobey
14:45 – 14:50	Summary Rounds 1 & 2 (definition)	Laurent Renesme
14:50 – 15:55	Round 3 voting (definition)	Manoj Lalu
15:55 – 16:00	Closing remarks	Bernard Thébaud

January 10 (Day 2): MSC Definition

Time (EST)	Activity	Speaker
14:00 – 14:05	Welcome	Bernard Thébaud
14:05 – 14: 10	Summary of Rounds 1 & 2	Laurent Renesme
14:10 – 15:45	Round 3 voting	Manoj Lalu/ Kelly Cobey
15:45 – 15:55	Implementation	Manoj Lalu/ Kelly Cobey
15:55 – 16:00	Closing remarks	Bernard Thébaud

NB: Due to the number of items to vote on for the MSC definition, we will start reviewing those items at the end of day 1.

Summary of Rounds 1 & 2

Our consensus meeting will take place over 2 days, where we will be providing summary results of the Round 1 and 2 survey results.

So far, for the **reporting guidelines**, 29 items have reached consensus for inclusion, and 4 items remain to discuss at our consensus meeting.

For the **MSC definition**, 6 items have reached consensus for inclusion or exclusion (1 item) and 18 items remain to discuss at our consensus meeting.

To reach **consensus, 80% or more of the participants** voting for the same level of agreement (disagree, neutral, agree) is required.

Summary of Rounds 1 & 2 Participants demographics

Participants characteristics		Round 1 N (%)	Round 2 N (%)
Origin			
	Africa	2 (2)	-
	Asia	7 (8)	-
	Australia / Oceania	6 (7)	1 (6)
	Europe	21 (24)	2 (12)
	North America	43 (49)	13 (76)
	South America	8 (9)	1 (6)
Gender			
	Male	55 (63)	13 (76)
	Female	32 (37)	4 (24)
Age			
	< 34	4 (5)	-
	35 - 44	22 (25)	1 (6)
	45 - 54	32 (37)	7 (41)
	55 - 64	24 (28)	8 (47)
	> 65	5 (6)	1 (6)
Career stage			
	Trainee	6 (7)	-
	Less than 5 years	4 (5)	-
	5 to 15 years	30 (34)	4 (25)
	> 15 years	43 (49)	12 (75)
Are you currently conducting research?			
	Yes	82 (94)	16 (94)
Type of research			
	Basic research	56 (31)	9 (53)
	Preclinical	59 (33)	11 (65)
	Clinical	39 (22)	10 (59)
	Methodologist	4 (2)	1 (6)
	Social science	4 (2)	1 (6)
	Regulatory science	8 (4)	2 (12)
Research area			
	Blood – Immune system	31 (11)	6 (35)
	Cancer	22 (8)	4 (23)
	Cardiovascular system	14 (5)	3 (18)
	Cell therapy	51 (18)	9 (53)
	Digestive system	3 (1)	1 (6)
	Ear-Nose and Throat (ENT)	4 (1)	1 (6)
	Endocrinology	5 (2)	1 (6)
	Musculoskeletal system	24 (8)	4 (23)
	Nervous system	13 (4)	1 (6)
	Regulatory	9 (3)	2 (12)
	Respiratory system	17 (6)	5 (29)
	Skin	9 (3)	1 (6)

Summary of Rounds 1 & 2 Reporting guidelines

Reporting guidelines – Items in consensus for inclusion

Mesenchymal Stromal cell (MSC) intervention group and control	
**Item**	**Description**
1	MSC administration route
2	MSC dose in the intervention group
3	MSC product concentration (i.e., concentration of the cell product administered to the patient)
4	The vehicle in which MSC are delivered to the patient
5	MSC solution infusion rate for MSC clinical studies using intra-venous route for MSC administration
6	Use of adjuvants during the preparation or processing of MSC (e.g., use of dimethyl sulfoxide (DMSO) for MSC preparation)
7	Characteristics of the control group when the study design involves a control group
8	The type of control used

Summary of Rounds 1 & 2 Reporting guidelines

Reporting guidelines – Items in consensus for inclusion

MSC characteristics			
**Item**	**Description**	**Item**	**Description**
9	MSC provenance	21	Method used to culture MSC (2D vs. 3D)
10	MSCs' Donor characteristics	22	Level of oxygen used for MSC culture
11	The tissue source of the MSC	23	Level of cell confluence
12	The extraction procedure used to obtain MSC from the tissue source	24	Culture medium used
13	Immune compatibility between MSC and patient	25	Use of serum for MSC culture
14	MSC state prior to administration (e.g., Fresh vs. cryopreserved)	26	Type of serum used
15	MSC conditioning prior to administration (if using cryopreserved MSC)	27	Amount of serum used
16	Functional assay performed on MSC product	28	Use of Human platelet lysate
17	Same batch vs. different batches	29	Amount of Human platelet lysate
18	MSC viability assessment		
19	Type of viability assay		
20	Results of viability assay		

Summary of Rounds 1 & 2 Reporting guidelines

Reporting guidelines – Items to continue to vote on

Item	Description	Responses	Round 1 N (%)	Round 2 N (%)
1	The MSC dose should be reported as a **dose normalized to weight** (number of cells per kilogram of bodyweight).	1-3 (Disagree)<br>4-6 (Neutral)<br>7-9 (Agree)	10 (14)<br>11 (16)<br>50 (70)	3 (19)<br>4 (25)<br>9 (56)
2	For studies using **cryopreserved** MSC, the number of months the cells were frozen prior to patient administration should be described.	1-3 (Disagree)<br>4-6 (Neutral)<br>7-9 (Agree)	14 (20)<br>21 (30)<br>35 (50)	5 (33)<br>3 (20)<br>7 (47)
3	The **population doubling time** (PDT) of the MSC used in the intervention group should be reported.	1-3 (Disagree)<br>4-6 (Neutral)<br>7-9 (Agree)	8 (12)<br>18 (27)<br>40 (61)	5 (33)<br>3 (20)<br>7 (47)
4	The media and reagents catalog numbers should be reported in the method section.	1-3 (Disagree)<br>4-6 (Neutral)<br>7-9 (Agree)	-	2 (12)<br>3 (19)<br>11 (69)

NB.

- Item #4 is a new item proposed by participants during round 1.
- To reach consensus, 80% or more of the participants voting for the same level of agreement (disagree, neutral, agree) is required.

Summary of Rounds 1 & 2 MSC definition

MSC Terminology – Items in consensus for inclusion

Terminology	
**Item**	**Description**
1	Mesenchymal Stromal Cell (MSC) is an appropriate term to maintain

Responses	Round 1		Round 2	
	N	%	N	%
Disagree (1-3)	5	7	2	13
Neutral (4-6)	14	19	0	0
Agree (7-9)	55	74	13	87
Total	74		15	

Summary of Rounds 1 & 2 MSC definition

MSC Definition – Items in consensus for inclusion

MSC characteristics	
**Item**	**Description**
2	A description of MSC positive and negative markers is essential to define them.
3	Positive cell markers : CD73+, CD90+, CD105+
4	Negative cell marker: CD45-
5	A description of where the MSC cells were sourced from is essential to characterize them
6	The following tissues are sources of MSC: bone marrow, Umbilical cord, adipose tissue, placenta, Dental follicle

MSC Definition – Items in consensus for exclusion

MSC characteristics	
**Item**	**Description**
1	Positive cell markers : SSEA-4+, Nestin+

Summary of Rounds 1 & 2
MSC definition

MSC terminology – Items to continue to vote on

Item	Description	Responses	Round 1 N (%)	Round 2 N (%)
1	Mesenchymal Stromal Cell and Mesenchymal Stem Cell are interchangeable terms	1-3 (Disagree)<br>4-6 (Neutral)<br>7-9 (Agree)	40 (50)<br>18 (22)<br>22 (28)	8 (57)<br>1 (7)<br>5 (36)

NB.

- To reach consensus, 80% or more of the participants voting for the same level of agreement (disagree, neutral, agree) is required.
- As the item "Mesenchymal Stromal Cell (MSC) is an appropriate term to maintain" reached consensus for inclusion (87%) , we will not vote on alternative denominations proposed during round 1.

Summary of Rounds 1 & 2 MSC definition

MSC definition– Items to continue <u>to vote on</u>

Item	Description	Responses	Round 1 N (%)	Round 2 N (%)
2	A description of MSC capacity to adhere to a plastic surface when maintained in standard culture condition, is essential to define them.	1-3 (Disagree)<br>4-6 (Neutral)<br>7-9 (Agree)	12 (17)<br>15 (22)<br>42 (61)	9 (70)<br>2 (15)<br>2 (15)
3	For MSC markers expression, the flow cytometry cut-off (% of cells) to consider a cell marker as a positive or a negative marker should be detailed in the Methods section.	1-3 (Disagree)<br>4-6 (Neutral)<br>7-9 (Agree)	5 (8)<br>12 (18)<br>49 (74)	2 (14)<br>4 (29)<br>8 (57)
4	For MSC markers expression, the flow cytometry results with the % of positive cells should be described for each positive and negative marker in the Results section.	1-3 (Disagree)<br>4-6 (Neutral)<br>7-9 (Agree)	9 (13)<br>11 (17)<br>46 (70)	5 (33)<br>4 (27)<br>6 (40)
5	The following <u>positive cell markers</u> essential to define MSC: CD29+, CD44+, CD166+, CD299+,CD10+, CD140+, CD142, CD271+, CD276+, HLA-I+, SSEA-3+			
6	The following <u>negative cell markers</u> essential to define MSC: CD3-, CD11-, CD14-, CD19-, CD31-, CD34-, HLA-DR-, CD11b-			
7	A description of MSC in-vitro differentiation capacity (e.g., differentiation in adipocytes, chondrocytes...etc.) is essential to define them.	1-3 (Disagree)<br>4-6 (Neutral)<br>7-9 (Agree)	21 (31)<br>13 (19)<br>34 (50)	4 (31)<br>6 (46)<br>3 (23)
8	The following differentiation assays are important to define MSC: Tri-lineage, adipocyte, osteoblast, chondroblast or none of those assays			
9	The MSC in-vitro differentiation capacity should be <u>qualitative</u>.	1-3 (Disagree)<br>4-6 (Neutral)<br>7-9 (Agree)	17 (26)<br>21 (32)<br>28 (42)	4 (27)<br>3 (20)<br>8 (53)
10	The MSC in-vitro differentiation capacity should be <u>quantitative</u>.	1-3 (Disagree)<br>4-6 (Neutral)<br>7-9 (Agree)	22 (34)<br>19 (29)<br>24 (37)	8 (53)<br>4 (27)<br>3 (20)

NB. To reach consensus, 80% or more of the participants voting for the same level of agreement (disagree, neutral, agree) is required.

Summary of Rounds 1 & 2 MSC definition

MSC definition– Items to continue to vote on

Item	Description	Responses	Round 1 N (%)	Round 2 N (%)
11	The following tissues are a source of MSC: Umbilical cord blood, synovial, peripheral blood, menstrual blood, iPSC and fetal tissue, Most tissues, amniotic fluid, virtually all the tissues			
12	A description of self-renewal and multilineage differentiation capacities is essential to define MSC.	1-3 (Disagree)<br>4-6 (Neutral)<br>7-9 (Agree)	23 (33)<br>12 (17)<br>35 (50)	5 (33)<br>8 (54)<br>2 (13)
13	The description of the specific method used to assess MSC stemness in-vitro is essential to define MSC.	1-3 (Disagree)<br>4-6 (Neutral)<br>7-9 (Agree)	15 (21)<br>16 (23)<br>39 (56)	4 (25)<br>6 (37.5)<br>6 (37.5)
14	A description of in-vitro functional assays (using quantitative RNA analysis of selected genes, proteins analysis of MSC secretome...etc.) to assess MSCs' potency and properties (e.g., trophic factors secretion, immunomodulation...etc.) is essential to characterize MSC.	1-3 (Disagree)<br>4-6 (Neutral)<br>7-9 (Agree)	10 (15)<br>17 (25)<br>41 (60)	4 (25)<br>2 (12)<br>10 (63)
15	MSC licensing, i.e. preconditioned in-vitro by pro-inflammatory cytokines exposure to mimic in vivo inflammatory environment, is essential to characterize MSC.	1-3 (Disagree)<br>4-6 (Neutral)<br>7-9 (Agree)	25 (42)<br>18 (31)<br>16 (27)	8 (53)<br>5 (33)<br>2 (14)
16	Molecules used for licensing should be described when defining MSC.	1-3 (Disagree)<br>4-6 (Neutral)<br>7-9 (Agree)	8 (14)<br>11 (18)<br>41 (68)	2 (13)<br>3 (20)<br>10 (67)
17	Resting (non-licensed) MSC should be used as an internal control when defining MSC.	1-3 (Disagree)<br>4-6 (Neutral)<br>7-9 (Agree)	11 (20)<br>11 (20)<br>34 (60)	1 (7)<br>3 (21)<br>10 (72)
18	Additional characteristics that are essential to define or characterize MSC: transcriptome analysis, secretome analysis, exosomes, immunomodulatory and MLR assays, angiogenic assays, transcription factors expression, DNA methylation profile.			

NB. To reach consensus, 80% or more of the participants voting for the same level of agreement (disagree, neutral, agree) is required.

Round 3 Voting explanation

In the upcoming voting, a team member will present each item and a summary of Round 1 and Round 2 Delphi study voting results.

There will be an opportunity for discussion to consider: "**What's essential now**?"

Following the discussion, all participants will be asked to re-vote as ‘Yes’, ‘No’, or ‘Abstain’ on each item.

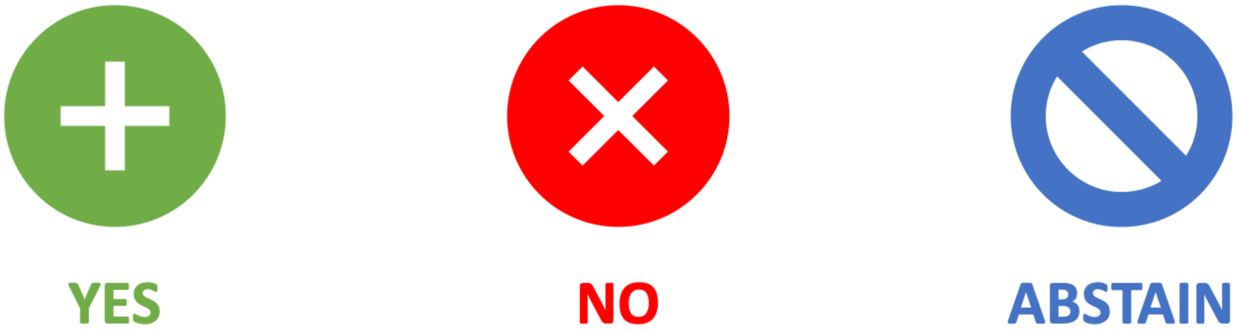

Core Group Members

Name	Affiliation
Dr Gerri BAER	U.S Food & Drug Administration (USA)
Dr Tania BUBELA	Simon Fraser University<br>Burnaby, British Colombia (Canada)
Dr John DE VOS	Institute for Regenerative Medicine and Biotherapy<br>Université de Montpellier, INSERM, CHU de Montpellier<br>Montpellier (France)
Dr Massimo DOMINICI	Laboratory of Cellular Therapy<br>University Hospital of Modena and Reggio Emilia<br>Modena (Italy)
Dr Rod DUNBAR	Maurice Wilkins Centre for Molecular Biodiscovery<br>University of Auckland<br>Auckland (New Zealand)
Dr Dean FERGUSON	Clinical Epidemiology Program<br>Ottawa Hospital Research Institute (OHRI)<br>Ottawa, Ontario (Canada)
Dr Daniel FREUND	Centre for Molecular and Cellular bioengineering<br>Technissche Universität Dresden Dresden (Germany)
Dr Jacques GALIPEAU	School of Medicine and Public Health<br>University of Wisconsin<br>Madison, Wisconsin (USA)
Dr Edwin M. HORWITZ	Marcus Center for Pediatric Advanced Cellular Therapy<br>Children's Healthcare of Atlanta<br>School of Medicine and Laney Graduate School<br>Emory University<br>Atlanta, Georgia (USA)
Dr Michael MATTHAY	School of Medicine<br>University of California San Francisco<br>San Francisco, California (USA)
Dr David MOHER	Centre for Practice Changing Research<br>Ottawa Hospital Research Institute (OHRI)<br>Ottawa, Ontario (Canada)

Core Group Members

Name	Affiliation
Dr Jan NOLTA	Institutes for Regenerative Cures<br>Davis Health System<br>University of California<br>Sacramento, California (USA)
Dr Graham C. PARKER	Integrative Health Science Facility Core<br>Wayne State University<br>Detroit, Michigan (USA)
Dr Donald G. PHINNEY	The Herbert Wertheim UF Scripps Institute for Biomedical Innovation & Technology<br>Jupiter, Florida (USA)
Dr Mahendra S. RAO	NIH Center of Regenerative Medicine<br>Labororatory of Stem Cell Biology<br>Bethesda, Maryland (USA)
Dr John RASKO	The Centenary Institute<br>Royal Prince Alfred Hospital<br>Sydney (Australia)
Dr Patricia R. M. ROCCO	Carlos Chagas Filho Institute of Biophysics (IBCCF)<br>Federal University of Rio de Janeiro<br>Rio de Janeiro (Brazil)
Dr Fabio ROSSI	Department of Medical Genetics<br>University of British Columbia<br>Vancouver, British Columbia (Canada)
Dr Michael ROSU MYLES	Regulatory Research Division<br>Centre for Biologics Evaluation, Biologics and Genetic Therapies<br>Health Canada<br>Ottawa, Ontario (Canada)
Dr Sowmya VISWANATHAN	Krembil Research Institute<br>University Health Network<br>Toronto, Ontario (Canada)

Organising Team's Biographies

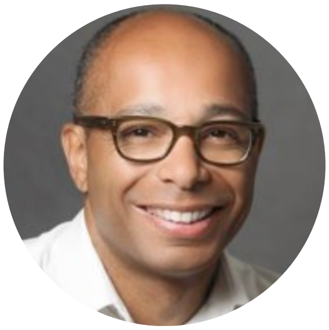

Dr. Bernard Thébaud is a clinician-scientist with a focus on the clinical translation of stem cell-based therapies for lung diseases. Dr. Thébaud is a senior scientist with the Ottawa Hospital Research Institute (OHRI) and Children's Hospital of Eastern Ontario Research Institute (CHEO RI), and a neonatologist with the Children's Hospital of Eastern Ontario (CHEO), where he provides care to critically ill newborns. He is a Professor of Pediatrics at the University of Ottawa and holds the uOttawa Partnership Research Chair in Regenerative Medicine.

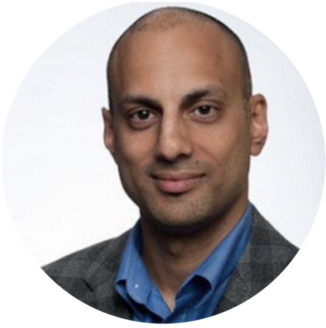

Dr. Manoj Lalu is an Anesthesiologist at The Ottawa Hospital and an Associate Scientist the OHRI (Clinical Epidemiology and Regenerative Medicine Programs). He is an Associate Professor in uOttawa's Department of Anesthesiology and Pain Medicine with a cross-appointment in the Department of Cellular and Molecular Medicine.
His current interests revolve around improving the preclinical to clinical translational pathway. This includes conducting preclinical and clinical systematic reviews, as well as meta-research on the conduct and reporting of laboratory-based studies.

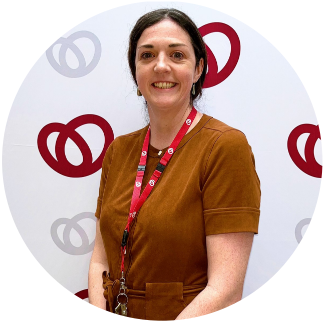

Dr. Kelly Cobey is a Scientist and the Director of the Metaresearch and Open Science Program at the University of Ottawa Heart Institute. She is also an Associate Professor in the School of Epidemiology and Public Health at uOttawa. As a trained Social Psychologist she bring mixed-methods expertise in quantitative and qualitative research practices, including survey design and focus group research. She is the co-chair of DORA (Declaration on Research Assessment) and her research interests focus on reproducibility, data management and sharing, and research reporting transparency.

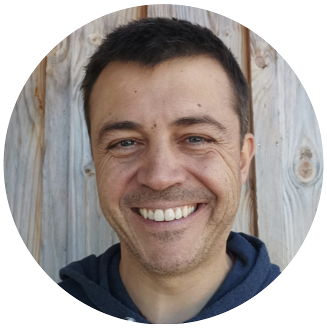

Dr. Laurent Renesme is a Neonatologist at the Children's Hospital of Eastern Ontario (CHEO) and the Ottawa Hospital (TOH) and an Assistant Professor at the University of Ottawa.He completed a PhD in Dr Thébaud lab working on strategies to improve and support clinical translation in neonatal lung research (including the use of organotypic culture of human fetal lung, study of human fetal lung development using single cell transcriptomic and the MSC Delphi project).

Funding and acknowledgement

STEM CELL NETWORK

Ottawa Hospital Research Institute
Institut de recherche de l'Hôpital d'Ottawa

XPLORE
Experimental Lung Organogenesis and Repair

BLUEPRINT
TRANSLATIONAL RESEARCH GROUP

UNIVERSITY OF OTTAWA HEART INSTITUTE
INSTITUT DE CARDIOLOGIE DE L'UNIVERSITÉ D'OTTAWA
